# Tolerogenic antigen-specific vaccine induces VISTA-enriched regulatory T cells and protects against arthritis in DRB1∗04:01 mice

**DOI:** 10.1016/j.ymthe.2025.04.034

**Published:** 2025-04-24

**Authors:** Laura Romero-Castillo, Rajan Kumar Pandey, Bingze Xu, Christian M. Beusch, Ana Oliveira-Coelho, Kejsi Zeqiraj, Carolin Svensson, Zhongwei Xu, Huqiao Luo, Outi Sareila, Pierre Sabatier, Changrong Ge, Lei Cheng, Vilma Urbonaviciute, Alexander Krämer, Cecilia Lindgren, Sabrina Haag, Johan Viljanen, Roman A. Zubarev, Jan Kihlberg, Anna Linusson, Harald Burkhardt, Rikard Holmdahl

**Affiliations:** 1 Medical Inflammation Research, Division of Immunology, Department of Medical Biochemistry and Biophysics, Karolinska Institute, 17176 Stockholm, Sweden; 2 Medical Inflammation Research, MediCity Research Laboratory, University of Turku, 20520 Turku, Finland; 3 Division of Physiological Chemistry I, Department of Medical Biochemistry and Biophysics, Karolinska Institute, 17176 Stockholm, Sweden; 4 Department of Chemistry-BMC, Uppsala University, 75237 Uppsala, Sweden; 5 Department of Chemistry, Umeå University, 90187 Umeå, Sweden; 6 Department of Pharmacological & Technological Chemistry, I. M. Sechenov First Moscow State Medical University, 119146 Moscow, Russia; 7 Fraunhofer Institute for Translational Medicine and Pharmacology ITMP, & Fraunhofer Cluster of Excellence for Immune-Mediated Diseases CIMD, Theodor-Stern-Kai 7, 60596 Frankfurt am Main, Germany; 8 Division of Rheumatology, University Hospital Frankfurt, Goethe University, 60596 Frankfurt am Main, Germany; 9 School of Medicine, Shanghai University, Shanghai 200444, China

**Keywords:** T cell tolerance, COL2, rheumatoid arthritis, regulatory T cells, VISTA, MHCII, tolerogenic, vaccine, immune checkpoints

## Abstract

Rheumatoid arthritis (RA) is a chronic autoimmune disease characterized by joint inflammation, cartilage damage, and bone erosion. Despite improvements with the introduction of biological disease-modifying anti-rheumatic drugs (DMARDs), RA remains an incurable life-long disease. Advancements in peptide-based vaccination may open new avenues for treating autoimmune diseases, including RA, by inducing immune tolerance while maintaining normal immune function. We have already demonstrated the efficacy of a potent vaccine against RA, consisting of the mouse major histocompatibility complex class II (A^q^) protein bound to the immunodominant type II collagen peptide COL2_259-273_, which needed to be galactosylated at position 264. To translate the vaccine to humans and to further enhance vaccine efficacy, we modified the glycine residue at position 265 and conjugated it with the human DRB1∗04:01 molecule. Remarkably, this modified vaccine (named DR4-AL179) provided robust effectiveness in suppressing arthritis in DRB1∗04:01-expressing mice without the need for galactosylation at position 264. DR4-AL179 vaccination induces tolerance involving multiple immunoregulatory pathways, including the activation of V-type immunoglobulin domain-containing suppressor of T cell activation (VISTA)-positive nonconventional regulatory T cells, which contribute to a potent suppressive response preventing arthritis development in mice. This modified RA vaccine offers a novel therapeutic potential for human autoimmune diseases.

## Graphical abstract

**Figure:**
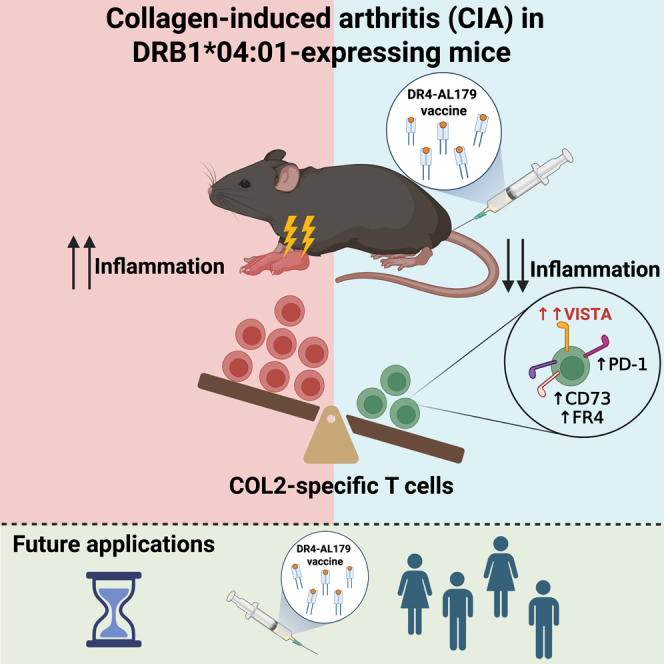

## Teaser

Romero-Castillo and colleagues developed a novel tolerogenic vaccine against rheumatoid arthritis using a modified collagen peptide bound to HLA-DRB1∗04:01. This vaccine induces VISTA-positive regulatory T cells that protect against arthritis in humanized mice without requiring complex galactosylation, offering a promising approach for treating autoimmune diseases.

## Introduction

Autoimmune diseases pose a growing health burden, affecting approximately 8% of the population worldwide.^1^^,^^2^^,^^3^ Despite their increasing prevalence, effective preventive and curative treatments for autoimmune conditions remain poorly explored. Among these autoimmune diseases, rheumatoid arthritis (RA) stands out as a common, severe, chronic inflammatory autoimmune disorder characterized by synovial joint inflammation, articular cartilage, and subchondral bone destruction.^4^^,^^5^ RA affects between 0.5% and 1% of the worldwide population, where women are affected 2–3 times more frequently than men.^6^^,^^7^ Unfortunately, current therapeutic interventions for RA cannot prevent or cure the disease while potentially increasing patients’ susceptibility to infection complications and other comorbidities.^8^

RA is genetically associated with the major histocompatibility complex class II (MHCII) region, particularly including the HLA-DRB1∗04:01 haplotype, indicating a key role for autoreactive T cells driving an immune response.^9^^,^^10^ Despite extensive research, RA’s primary autoantigen responsible for T cell activation remains unidentified. However, type II collagen (COL2), a major component of hyaline cartilage, has emerged as one potential autoantigen. This notion is supported by the frequent observation of both potentially pathogenic and protective B cells as well as anti-COL2 antibodies in RA patients,^11^^,^^12^ along with the susceptibility of experimental animals to collagen-induced arthritis (CIA).^13^^,^^14^^,^^15^ Notably, a COL2 peptide is presented to T cells in mice expressing the MHCII A^q^ molecule, similar to how it is presented in individuals expressing RA-associated HLA-DRB1∗04:01 molecules.^14^^,^^16^^,^^17^ These findings underscore the potential role of COL2-specific immune responses in the pathogenesis of RA.

Considering these findings, we propose to induce immune tolerance by using a tolerogenic vaccine to rebalance the immune homeostasis and potentially cure RA. Tolerogenic vaccination seeks to establish peripheral tolerance against self-antigens while preserving protective immune responses.^18^ The discovery of autoreactive T cells specific for the COL2_259-273_ peptide, with or without glycosylation in RA patients carrying the HLA-DRB1∗04:01 allele,^19^^,^^20^ allows an opportunity to modify these cells in a regulatory direction. By inducing a regulatory T cell-dependent response, such a vaccine could alleviate self-reactive immune cells and mitigate autoimmune pathology.

Several strategies have been explored to develop tolerogenic interventions, including whole-antigen-based tolerance,^21^^,^^22^ antigen-loaded particles,^23^^,^^24^ and soluble MHCII-peptide complexes.^25^^,^^26^ Additionally, the adoptive transfer of conventional regulatory T cells (Foxp3+ Tregs)^27^ or tolerogenic dendritic cells^28^^,^^29^ has shown promise in inducing immune tolerance and alleviating autoimmune disorders in preclinical models. However, controlling the immune response remains challenging. Some strategies involving antigen-presenting cells (APCs) may inadvertently lead to undesired immune activation. This potential risk arises when certain co-stimulatory signals shift the T cell response toward a pro-inflammatory or aggressive phenotype, rather than fostering regulatory T cell induction, thereby exacerbating rather than ameliorating immune-mediated pathology.

It has become clear that the development of tolerogenic vaccines requires a detailed understanding of both the vaccine’s characteristics and the *in vivo* mechanisms driving immune tolerance. These insights are crucial for achieving precise control over the immune response and ensuring consistent and reliable induction of tolerance.

Our group has previously demonstrated that soluble MHCII (A^q^) molecules complexed with the galactosylated COL2_259-273_ peptide are effective in treating CIA in mice interacting with the COL2-specific T cell receptor (TCR).^25^^,^^26^ Moving forward, we aim to translate these findings into viable therapeutic interventions for human RA patients using DRB1∗04:01 combined with a COL2 peptide. To validate the vaccine, our laboratory has developed unique knockin humanized mouse models expressing physiological levels of human MHCII molecules, providing a more accurate platform for evaluating potential RA treatments^14^ than MHCII transgenics mouse models previously used. In this study, we identified a novel chemical modification at position 265 of the non-galactosylated COL2_259-273_ peptide, bound to recombinant human MHCII (DRB1∗04:01), which resulted in an enhanced tolerogenic vaccination effect. Similar to the galactosylated variant, the new vaccine incorporating the modified ligand (DR4-AL179), which lacks galactosylation, exerts its effect through a distinct set of regulatory T cells. These cells express V-type immunoglobulin (Ig) domain-containing suppressor of T cell activation (VISTA),^30^ also known as programmed death-1H (PD-1H) and encoded by the *VSIR* gene.

## Results

### Screening of modified COL2-peptides as potential vaccine candidates for RA

**Figure 1:**
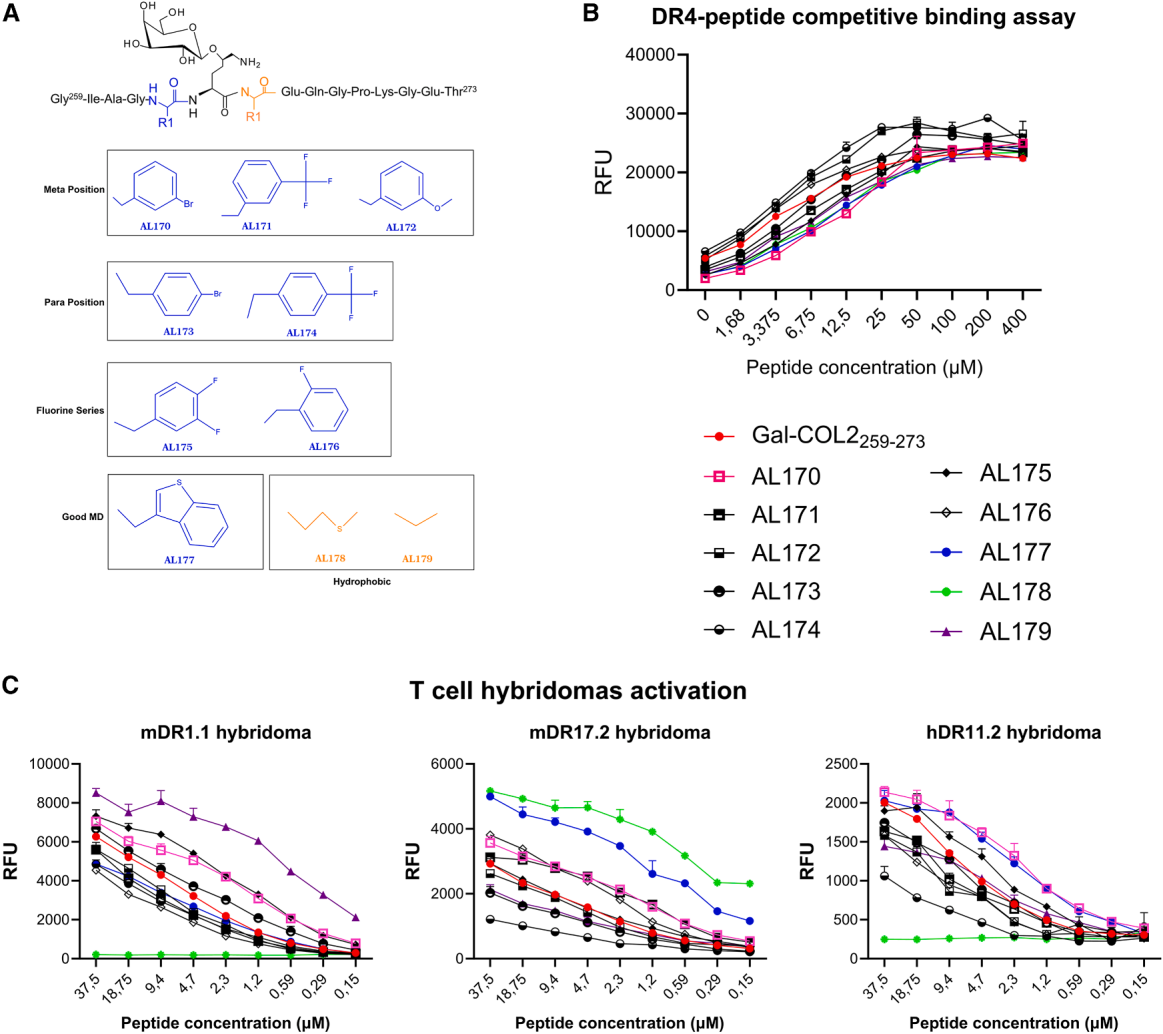
Design and evaluation of modified COL2_259-273_ glycopeptides as vaccine candidates (A) Schematic representation of synthesized COL2 glycopeptide modifications. The parent peptide gal-COL2_259-273_ (galactosylated at lysine 264, galCOL2_259-273_) was modified at either phenylalanine 263 (blue) or glycine 265 (orange). Phenylalanine modifications included meta-position substituents (AL170, AL171, and AL172), para-position substituents (AL173 and AL174), fluorine derivatives (AL175 and AL176), and molecular-dynamics-optimized modification (AL177). Glycine was modified with hydrophobic substituents (AL178 and AL179). (B) DRB1∗04:01 (DR4) competitive-binding assay comparing modified peptides to parent gal-COL2_259-273_. (C) T cell-activation assay using three DR4-gal-COL2-restricted T cell hybridoma clones (mDR1.1, mDR17.2, and hDR11.2). T cell activation was assessed by measuring IL-2 secretion into the medium by ELISA. RFU, relative fluorescence units.

To develop improved vaccine candidates, we conducted *in vitro* testing of a range of modified glycopeptides derived from the parent peptide COL2_259-273_, galactosylated at lysine 264 (galCOL2_259-273_) (Figure 1A). Our design strategy focused on two key residues essential for TCR recognition: phenylalanine 263 and glycine 265. We systematically evaluated each modified peptide against the parent galCOL2_259-273_, assessing both MHCII-binding capacity (Figure 1B) and T cell activation potential using glycosylated epitope-specific T cell hybridoma clones (Figure 1C). The DR4-binding evaluation revealed that most modified peptides retained binding affinities comparable to the parent gal-COL2_259-273_ peptide (Figure 1B). In T cell activation assays, AL170 exhibited an enhancement in T cell response across all hybridoma clones tested. AL177 and AL178 selectively increased activation of the mDR17.2 clone, while AL179 significantly stimulated the mDR1.1 clone (Figure 1C). Through this comprehensive screening, four peptides emerged as lead candidates (AL170, AL177, AL178, and AL179) distinguished by their enhanced MHCII binding and alterations in T cell contact points.

**Figure 2:**
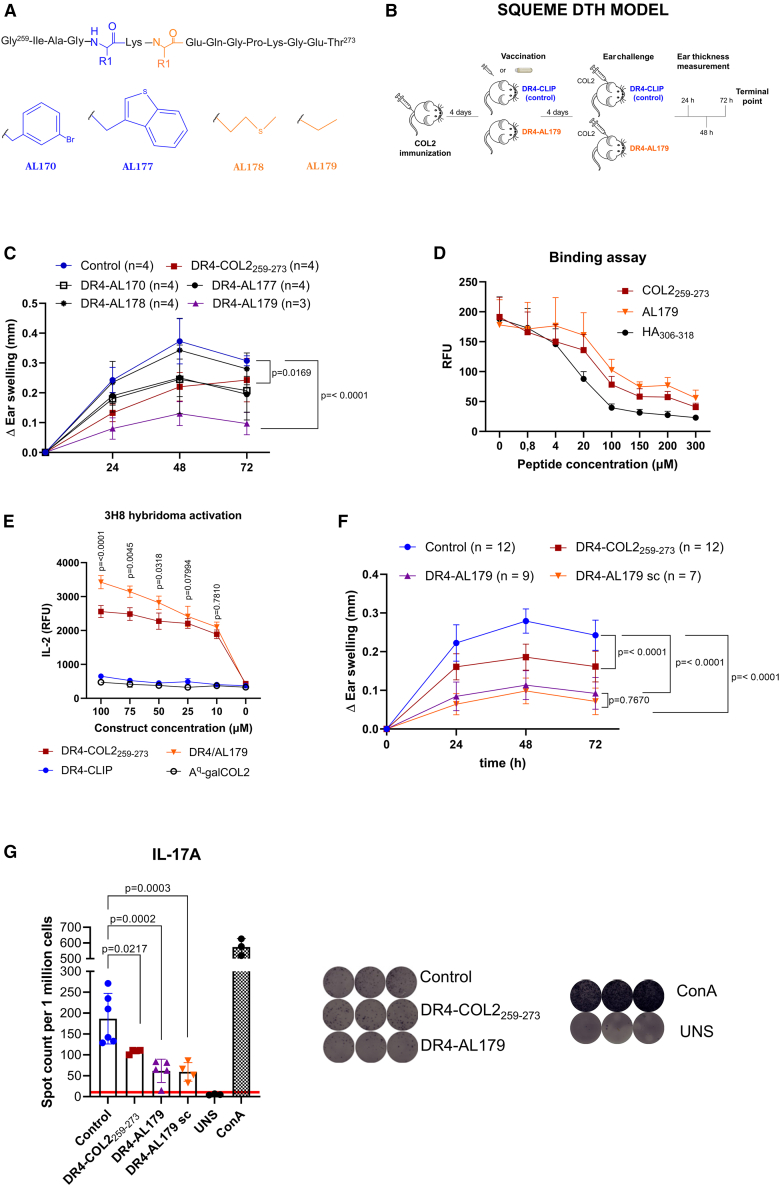
The synthetic modified-COL2 peptide AL179 stands out as the best vaccine candidate (A) Schematic design of the synthesized non-galactosylated COL2-peptides. In blue are peptide modifications on the phenylalanine residue at position 263 and, in orange, those on glycine residue at position 265. (B) Experimental procedure of the DTH model. Parker mice were immunized with COL2/CFA i.d. and 4 days later, mice were vaccinated s.c. with DR4-peptide complexes using either osmotic pumps or three injections. 8 DPI, mice were challenged i.d. in the ear with 10 μg of bCOL2 or 10 μg of acetic acid. Ear thickness was measured using a caliper at 24, 48, and 72 h post challenge. Mice were terminated 72 h post challenge. (C) DTH reactions (ear swelling) following treatment with DR4-COL2 complexes using osmotic pumps. Number of mice per group are indicated in parentheses. (D) Binding affinity of AL179 and COL2_259-273_ peptides with the DRB1∗04:01 molecule. Influenza hemagglutinin (HA) peptide (HA_306-318_) was used as a positive control. Data are representative of three experiments. (E) Native-COL2-specific T-hybridoma (3H8) activation by DR4-COL2 complexes. T cell activation was assessed by measurement of the IL-2 secreted into the medium by ELISA. DR4-CLIP and A^q^-galCOL2 complexes were used as negative controls. Data are representative of three experiments. (F) DTH reactions (ear swelling) following treatment with DR4-COL2 complexes using osmotic pumps or s.c. injections. Numbers of mice per group are indicated in parentheses. (G) Measurement of antigen-specific IL-17A T cell response against the COL2 protein assessed by ELISpot in inguinal lymph nodes 72 h after the DTH model. ConA was used as a positive control (signal exceeds 600 SFC/10^6^ cells) while unstimulated cells (UNS) incubated in complete medium alone served as a negative control. The red line indicates the positive threshold determined using the distribution-free resampling (DFR(2×)) test, a standard method for ELISpot analysis. Representative ELISpot wells show antigen-specific IL-17A production by lymph node cells from vaccinated mice. (C–G) Results are expressed as mean ± SEM. Statistics were determined using a two-way ANOVA with Sidak’s multiple-comparisons test. Control, DR4-CLIP.

Due to the presence in humans of autoreactive T cells specific for non-glycosylated COL2,^31^ we opted for the development of a non-galactosylated vaccine. Additionally, the complexity of synthesizing glycosylated vaccines presents significant challenges for scalability and further development. The selected peptides are depicted in Figure 2A.

To screen their functional effect, we used the delayed-type hypersensitivity (DTH) model mediated by interferon (IFN)-γ-producing type 1 T helper (Th1) cells, a test predicting an arthritis protective effect.^26^ Mice were vaccinated using osmotic pumps utilizing various complexes comprising human MHCII (DRB1∗04:01, named DR4) combined with either the unmodified COL2_259-273_ peptide or modified peptides (AL170, AL177, AL178, and AL179). A DR4 complex containing a mutated CLIP (class II-associated invariant chain peptide) served as the control. The experimental scheme is depicted in Figure 2B. Among the peptides tested, AL179 exhibited the most efficient reduction in ear swelling compared to the control group (Figure 2C).

AL179 bound well to MHCII and demonstrated enhanced activation of COL2-specific T cell hybridoma cells (3H8) in comparison with DR4-COL2_259-273_ (Figures 2D and 2E). In parallel, docking analysis of DR4-COL2_259-273_ and DR4-AL179 with the TCR revealed an increased number of hydrogen bonds reflecting a stronger binding affinity and stabilization of the complex formation between DR4-AL179 and TCR compared to DR4-COL2_259-273_ (Figure S1). Furthermore, recognizing the need for a more applicable administration route for human translation, we compared osmotic pump delivery with three subcutaneous (s.c.) administrations every 4 h. Our findings revealed no discernible differences in ear swelling or interleukin (IL)-17A secretion (Figures 2F and 2G, respectively), prompting the selection of the latter route for subsequent experiments.

### DR4-AL179 vaccine alleviates autoimmune arthritis

**Figure 3:**
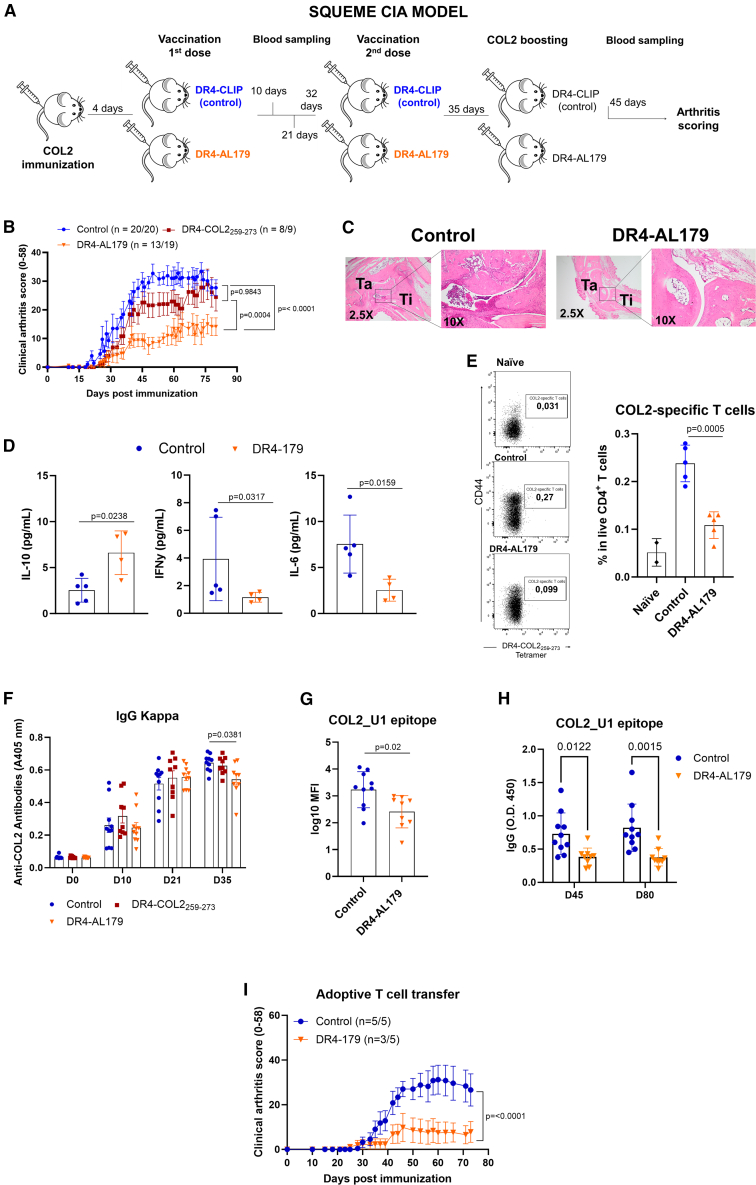
DR4-AL179 vaccination reduces arthritis severity and modulates immune responses in the CIA model (A) Experimental procedure of the CIA model. Parker mice were immunized with COL2/CFA i.d. and at 4 and 32 DPI mice were vaccinated s.c. with DR4-peptide complexes. At 35 DPI, the mice were boosted with 50 μg of bCOL2 in IFA i.d. Blood samples were collected at 10, 21, 35, and 45 DPI and at the terminal point. (B) Arthritis severity was scored throughout the experiment. CIA in vaccinated mice as assessed by mean clinical score of arthritis severity and incidence of arthritis (number of arthritic mice/total number of mice). Data from two pooled experiments. (C) Representative histological examination by H&E staining of the tibia (Ti) and talus (Ta) from control and DR4-AL179-vaccinated mice. Cell infiltration and cartilage/bone destruction are higher in the control group. (D) Serum cytokine levels of IL-10, IFN-γ, and IL-6 after 20 DPI. (E) Vaccination with DR4-AL179 complexes reduces frequencies of COL2-specific T cells (CD44+ DR4-COL2_259-273_ tetramer+) compared to DR4-CLIP (control) vaccinated mice at day 80 (terminal point) of the CIA model (B). Naive Parker mice (non-COL2 immunized) were used as a negative control. Representative gates are shown. (F) Anti-COL2 antibody levels of total IgG (anti-kappa) from vaccinated mice at different time points along CIA model measured by ELISA. (G) IgG antibodies binding to the triple-helical COL2 epitope U1 in sera collected from vaccinated mice at 45 DPI measured by bead-based flow immunoassay and quantified as log10-transformed mean fluorescence intensity (MFI). (H) IgG antibodies binding to the triple-helical COL2 epitope U1 in sera collected from vaccinated mice at 45 and 80 DPI measured by ELISA. (I) Clinical scoring of arthritis severity following the transfer of T cells from DR4-CLIP-vaccinated and DR4-AL179-vaccinated Parker mice into COL2-immunized recipient Parker mice. The numbers of arthritic mice/total number of mice are represented. Results are expressed as mean ± SEM. Each dot represents an individual mouse. Statistics in (B), (E), (F), and (H) experiments represented were determined using two-way ANOVA with Sidak’s multiple-comparisons test, and in (D), (G), and (I) were determined with a two-tailed Mann-Whitney test. Control, DR4-CLIP; OD, optical density.

Next, we assessed the efficacy of the DR4-AL179 vaccine in ameliorating arthritis in the CIA model. The experimental scheme is depicted in Figure 3A. Treatment with DR4-AL179 significantly reduced both the incidence and the severity of arthritis (Figure 3B). In contrast, the native non-glycosylated DR4-COL2_259-273_ vaccine did not affect arthritis (Figure 3B) despite previously showing a reduction in ear swelling in the DTH model (Figures 2C and 2F). The therapeutic effect of DR4-AL179 in the CIA model was further supported by histological analysis of paw sections, revealing reduced immune cell infiltration and bone erosion (Figures 3C and S2A) along with enhanced preservation of collagen fiber structure (Figure S2B) compared to the control group. Additionally, serum cytokine analysis on day 20 post immunization revealed a marked increase in the anti-inflammatory IL-10 cytokine and a reduction in the pro-inflammatory cytokines IFN-γ and IL-6 (Figure 3D) after vaccination with DR4-AL179 complex. Notably, spleen analysis at the terminal point (day 80) showed a significant reduction in COL2-specific T cells in the DR4-AL179-vaccinated group (Figure 3E). The gating strategy of COL2-specific T cells at CIA terminal point is depicted in Figure S3.

Moreover, vaccination with DR4-AL179 resulted in a notable decrease in anti-COL2 IgG antibody levels in comparison to the control group vaccinated with DR4-CLIP (Figure 3F). This finding was corroborated by a bead-based immunoassay that detected epitope-specific anti-COL2 peptide IgG antibodies, revealing both epitopes spreading over time and a marked reduction in antibody levels in the DR4-AL179 vaccination group, particularly against most COL2 peptides at day 45 post immunization (Figure S4). Remarkably, DR4-AL179 vaccination significantly reduced the arthritogenic U1 antibody^32^ levels during both the acute phase of disease at day 45 post immunization (Figure 3G) and at the terminal point (Figure 3H).Finally, we demonstrated that the suppressive effect of T cells from DR4-AL179-vaccinated mice could be transferred, protecting recipient mice from CIA development (Figure 3I).

### Vaccination with DR4-AL179 expands antigen-specific CD4+ tolerogenic T cells

**Figure 4:**
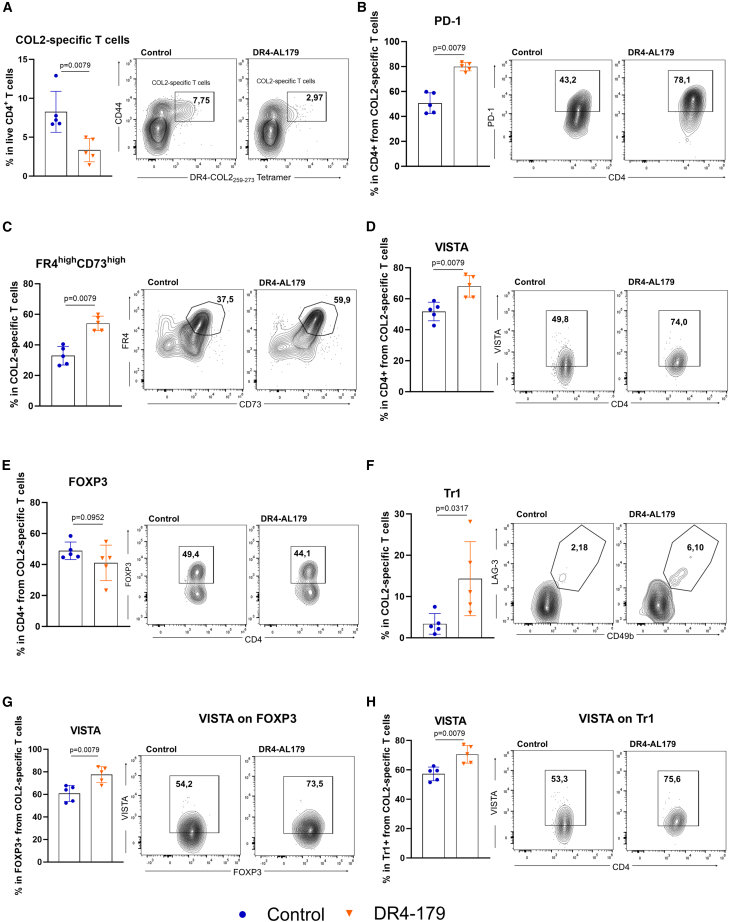
DR4-AL179 vaccination enhances a tolerogenic phenotype in COL2-specific T cells (A) Vaccination with DR4-AL179 complexes reduces frequencies of COL2-specific T (CD44+ DR4-COL2_259-273_ tetramer+) cells after 15 DPI. Parker mice were immunized with COL2/CFA, and at 4 DPI mice were vaccinated with DR4–peptide complexes by three s.c. injections. 15 DPI splenocytes and dLN cells were recovered, stained with the PE-labeled DR4-COL2_259-273_ tetramer, and enriched using anti-PE magnetic beads for immunophenotyping by flow cytometry. Representative flow cytometry plots depicting the gating strategy for DR4-COL2_259-273_+/CD44+ (COL2-specific) cells in live CD4+ T cells. (B–D) Vaccination with the DR4-AL179 complexes increases the frequency of PD-1, CD73, FR4, and VISTA in COL2-specific T cells. Representative flow cytometry plots depicting the gating strategy for PD-1, CD73, FR4, and VISTA in COL2-specific T cells (CD44+ DR4-COL2_259-273_ tetramer+). (E) Frequency of FOXP3+ in CD4+ from COL2-specific T cells. Representative flow cytometry plots depicting the gating strategy for FOXP3 in CD4+/CD44+/DR4-COL2_259-273_T cells. (F) Frequency of Tr1 (LAG-3+/CD49+) in COL2-specific T cells. Representative flow cytometry plots depicting the gating strategy for LAG-3+/CD49+ (Tr1) in CD4+/CD44+/DR4-COL2_259-273_T cells. (G) Frequency of VISTA+ in FOXP3+ from COL2-specific T cells. Representative flow cytometry plots depicting the gating strategy for VISTA+ in FOXP3+/DR4-COL2_259-273_+/CD44+/CD4+ T cells. (H) Frequency of VISTA+ in Tr1 from COL2-specific T cells. Representative flow cytometry plots depicting the gating strategy for VISTA+ in LAG-3+/CD49+/DR4-COL2_259-273_+/CD44+/CD4+ T cells. Results are expressed as mean ± SEM. Each dot represents an individual mouse. Statistics were determined with a two-tailed Mann-Whitney test. Control, DR4-CLIP.

We found that the administration of DR4-AL179 to Parker mice led to a reduction in the number of COL2-specific T cells (Figure 4A). The COL2-specific T cells from DR4-AL179-vaccinated mice were investigated for phenotypes earlier selected to be of importance for A^q^-COL2 vaccination.^26^ Clearly, the T cells from the DR4-AL179-vaccinated mice had an increased expression of cell markers associated with a tolerogenic phenotype as compared with the DR4-CLIP control group. We observed higher PD-1 expression after DR4-AL179 vaccination (Figure 4B). Similarly, CD73, folate receptor 4 (FR4) (Figure 4C) and VISTA (Figure 4D) were upregulated after vaccination with DR4-AL179. It has been reported that CD73^high^FR4^high^ T cells have the capacity to differentiate into either FOXP3-expressing Tregs or IL-10-producing CD49+LAG-3+ unconventional regulatory T cells (Tr1 cells) depending on the specific physiological context.^33^^,^^34^^,^^35^ However, in this sense, we observed that vaccination with DR4-AL179 did not increase the FOXP3 conventional regulatory T cells (Figure 4E) but rather increased the non-classical regulatory T cells, the Tr1 population (Figure 4F).

VISTA, a member of the B7 family of immune inhibitory molecules, is known to negatively regulate immune responses, maintain peripheral tolerance, and control autoimmunity.^36^ Our previous work in tolerogenic vaccination hypothesized that VISTA plays a crucial role in vaccination efficacy in A^q^ mice.^26^ Literature suggests a higher expression of VISTA on regulatory T cells.^37^ Similarly, we observed a higher VISTA expression on FOXP3 Tregs (Figure 4G) and in Tr1 T cells (Figure 4H), although no difference in FOXP3 expression after vaccination with DR4-AL179 was observed (Figure 4E). The gating strategy and a summary table of the flow cytometry analyses shown in Figure 4 are presented in Figure S5.

Although vaccination with DR4-COL2_259-273_ failed to significantly ameliorate autoimmune arthritis in the CIA model (Figure 3B), it induced elevated expression of PD-1 and VISTA on COL2-specific T cells and increased the frequency of CD73^high^FR4^high^ T cells (Figure S6). Similar changes were observed following DR4-AL179 vaccination. However, the increase in VISTA expression and frequency of CD73^high^FR4^high^ T cells was less pronounced in DR4-COL2_259-273_-vaccinated mice compared to those receiving DR4-AL179.

### The role of VISTA in shaping vaccine efficacy

**Figure 5:**
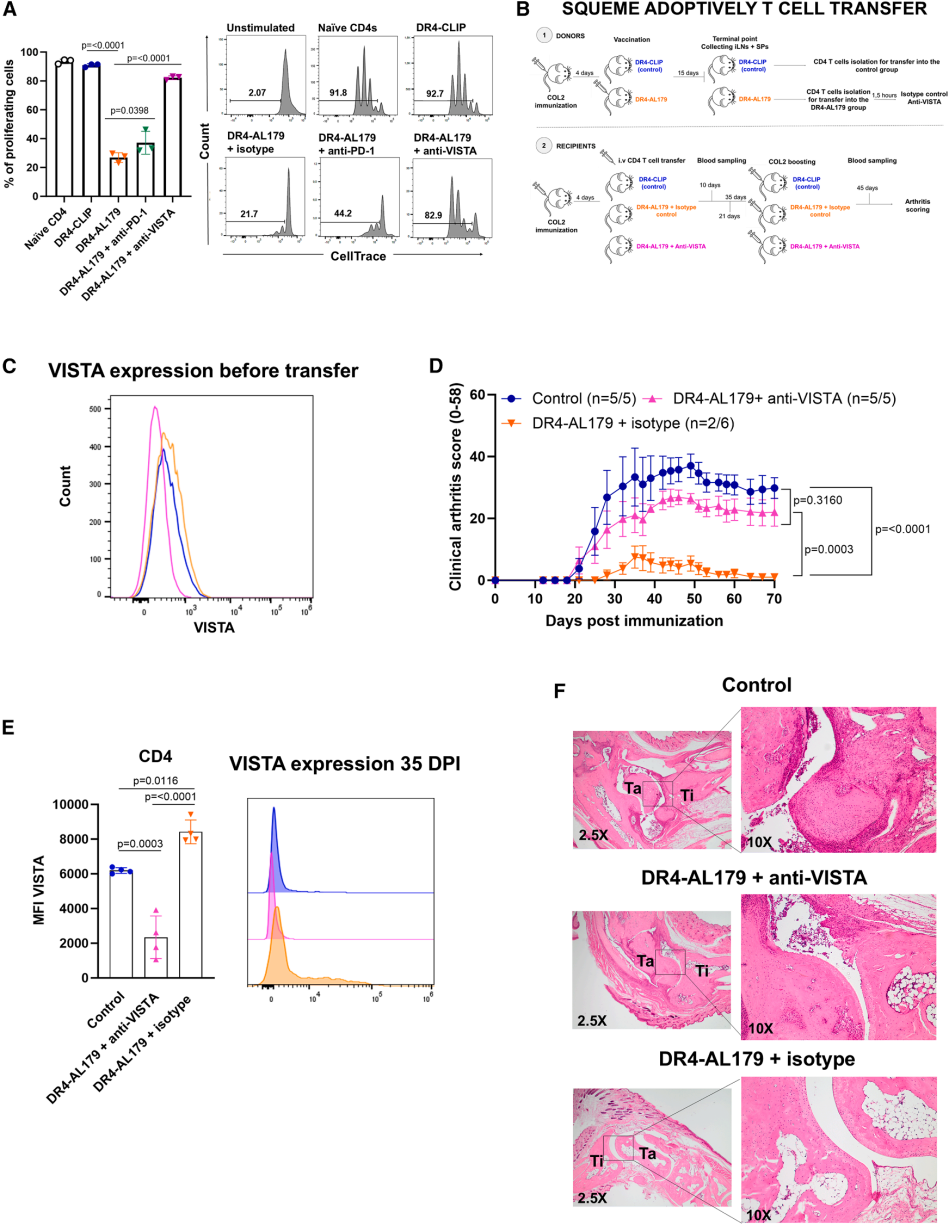
Impact of VISTA on the vaccine effectiveness (A) CD4+ T cells from DR4-AL179-vaccinated Parker mice suppress anti-CD3/CD28-induced polyclonal T cell proliferation. Parker mice were immunized with COL2/CFA, and at 4 DPI mice were vaccinated with DR4–peptide complexes (DR4-CLIP or DR4-AL179) by 3 s.c. injections. 15 DPI splenocytes and dLN cells were recovered and CD4+ T cells were purified and co-cultured with purified violet CellTrace-labeled CD4+ T cells from naive (non-COL2-immunized) Parker mice. CD4+ T cells from DR4-AL179-vaccinated mice were incubated for 1 h at 4°C with 100 μg/mL anti-VISTA, anti-PD-1, or isotype control antibodies. Afterward, cells were washed and added to the naive Parker T cells. Flow cytometry was performed 3 days after stimulation with anti-CD3/CD28 antibodies. Quantification (left) and representative gating (right) are shown. (B) Experimental procedure of the adoptive T cell transfer. Donor Parker mice were immunized with COL2/CFA i.d. and, 4 days later, the mice were vaccinated s.c. with DR4-peptide complexes (DR4-CLIP or DR4-AL179). At 15 DPI, donor mice were terminated, and their dLNs and spleens were collected. CD4+ T cells were isolated using MACS. CD4+ T cells from DR4-AL179-vaccinated mice were divided into two groups and incubated for 1.5 h with either 100 μg/mL of isotype control or anti-VISTA antibodies. These CD4+ T cells were then transferred i.v. into the recipient mice, which had been immunized i.d. with 100 μg of bCOL2 in CFA 4 days prior. At 35 DPI, the recipient mice were boosted with 50 μg of bCOL2 in IFA i.d. Blood samples were collected at 10, 21, 35, and 45 DPI and at the terminal point. Arthritis severity was scored throughout the experiment. (C–F) Adoptive transfer of CD4+ T cells from DR4-AL179-vaccinated mice transferred into COL2-immunized recipient Parker mice. (C) The expression of VISTA in CD4+ T cells within the different groups was assessed by flow cytometry before transfer. (D) Arthritis clinical score in recipient mice after T cell transfer. The numbers of arthritic mice/total number of mice are shown. (E) The expression of VISTA in CD4+ T cells within the different groups was assessed by flow cytometry after 35 DPI in peripheral blood. (F) Representative histological examination by H&E staining of the tibia (Ti) and talus (Ta) assessment in recipient mice. Results are expressed as mean ± SEM. Each dot represents an individual mouse. Statistics were determined by using two-way ANOVA with Sidak’s multiple-comparisons test. Control, DR4-CLIP.

Building upon our findings in A^q^ mice, we extended our investigation of VISTA’s role in vaccination efficacy to our humanized mouse model by conducting an *ex vivo* T cell suppression assay. Ten days post immunization, CD4+ T cells were enriched from the spleens of vaccinated mice and co-cultured with purified, CellTrace-labeled CD4+ T cells from naive Parker mice, which were activated with anti-CD3 and anti-CD28 antibodies. The CD4+ T cells from vaccinated mice were pretreated with anti-VISTA, anti-PD-1, or isotype control antibodies. Blocking VISTA resulted in a reduced suppressive effect compared to the isotype control, whereas blocking PD-1 had no significant impact (Figure 5A). The gating strategy of the suppression assay is presented in Figure S7.

Following the findings in the suppression assay, we further investigated VISTA’s influence on vaccination efficacy by performing an adoptive transfer experiment. CD4+ T cells from vaccinated mice were pretreated with either anti-VISTA or isotype control antibodies and transferred into immunized recipient Parker mice. A control group received T cells from unvaccinated mice. The experimental scheme is depicted in Figure 5B. VISTA expression levels in different groups were assessed by flow cytometry before transfer (Figure 5C). Anti-VISTA treatment impaired the therapeutic capacity of T cells to ameliorate autoimmune arthritis, while T cells treated with the isotype control effectively suppressed disease progression (Figure 5D), consistent with findings from the *ex vivo* T cell-suppression assay (Figure 5A). Moreover, in peripheral blood 35 days post immunization (DPI), VISTA expression remained upregulated in the DR4-AL179 + isotype group compared to controls, whereas it was significantly downregulated in the DR4-AL179 + anti-VISTA group (Figure 5E). Histological analysis of joint sections confirmed disease severity, revealing immune cell infiltration and bone erosion (Figures 5E and S8A) in both DR4-CLIP and DR4-AL179 + anti-VISTA groups, accompanied by significant degradation of collagen fiber architecture (Figure S8B) compared to the DR4-AL179 + isotype control group.

### Proteomic changes in T and B cells following adoptive T cell transfer from DR4-AL179 or DR4-CLIP-vaccinated mice

In the context of the adoptive transfer experiment, we analyzed the proteomic landscape of both CD4+ T cells and B cells isolated from the dLNs and spleens of recipient mice at the terminal time point. The objective was to identify specific proteomic shifts to characterize the downstream pathways.

**Figure 6:**
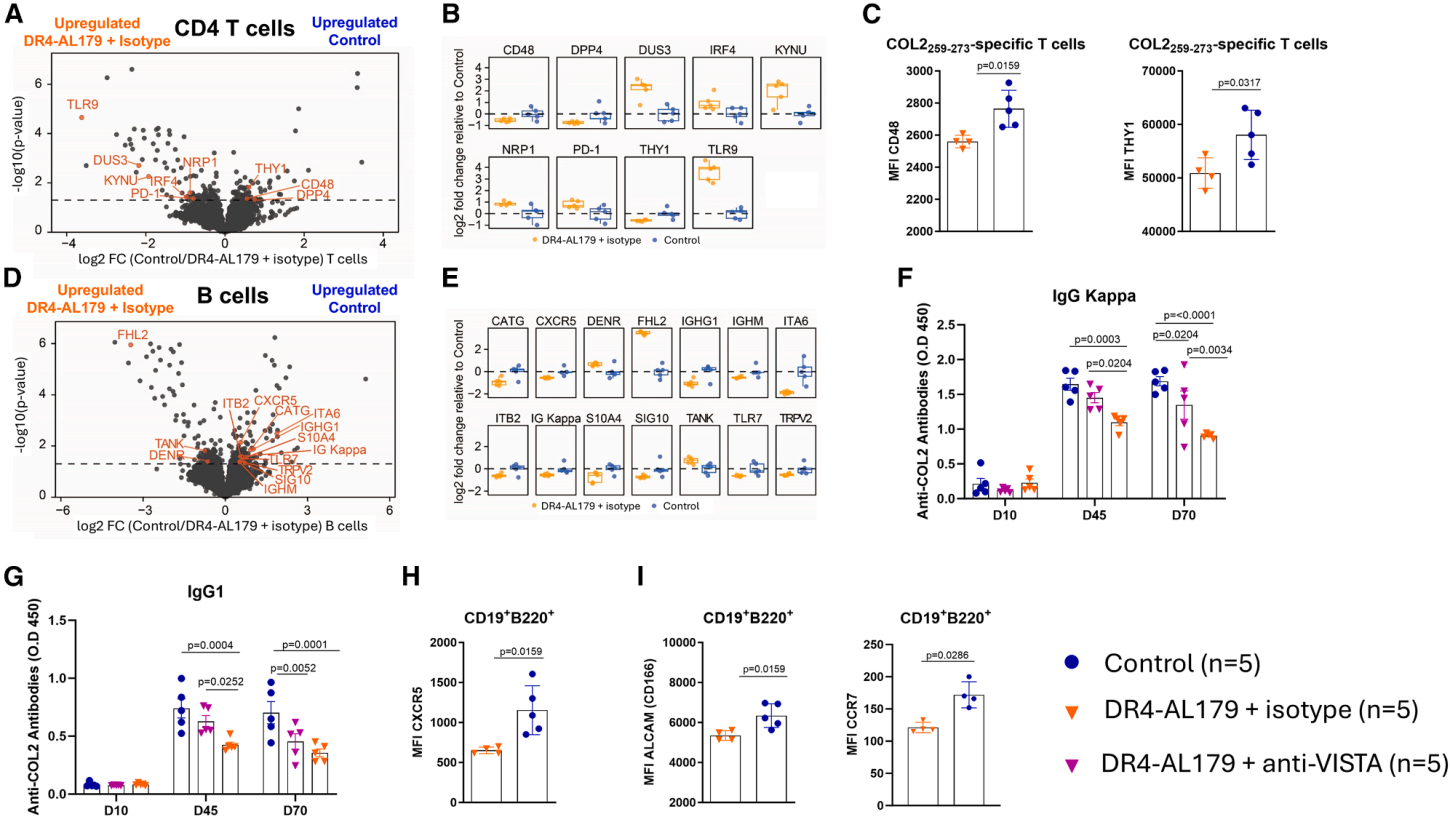
Impact of tolerogenic vaccination on T and B cell proteomic landscape (A) Volcano plot comparing differently expressed proteins on sorted CD4+ T cells between DR4-AL179 isotype (left, *n* = 5) and DR4-CLIP (right, *n* = 5) adoptively transferred mice. (B) Boxplots showing selected differentially expressed proteins comparing sorted CD4+ T cells from DR4-AL179 isotype and DR4-CLIP (*n* = 5 each) adoptively transferred mice. The horizontal line in the boxplots represents the median, 25^th^, and 75^th^ percentiles and the whiskers represent measurements to the 5^th^ and 95^th^ percentiles. (C) CD48 and THY1 MFI on COL2-specific T cells 15 DPI in COL2-immunized Parker mice. (D) Volcano plot comparing differently expressed proteins on B cells between DR4-AL179 isotype (left, *n* = 5) and DR4-CLIP (right, blue, *n* = 5) adoptively transferred mice. (E) Boxplots showing selected differentially expressed proteins comparing sorted B cells from DR4-AL179 isotype and DR4-CLIP (*n* = 5 each) adoptively transferred mice. The horizontal line in the boxplots represents the median, 25^th^, and 75^th^ percentiles and the whiskers represent measurements to the 5^th^ and 95^th^ percentiles. (F- and G) Anti-COL2 antibody levels of total IgG (anti-kappa) and of IgG1, respectively, from adoptively transferred mice at different time points measured by ELISA. (H) CXCR5 MFI on B cells 15 DPI in COL2-immunized Parker mice. (I) ALCAM and CCR7 MFI on B cells 15 DPI in COL2-immunized Parker mice. Results are expressed as mean ± SEM. Each dot represents an individual mouse. Statistics in (C), (H), and (I) were determined with a two-tailed Mann-Whitney test and in (F) and (G) were determined by using two-way ANOVA with Sidak’s multiple-comparisons test. Control, DR4-CLIP.

An upregulation of PD-1 protein was observed in fluorescence-activated cell sorting (FACS)-sorted CD4+ T cells from DR4-AL179 + isotype-treated mice (Figures 6A and 6B). This finding is consistent with our previous observations in COL2-specific T cells analyzed by flow cytometry (Figure 4B).

To ensure the results in Figures 6A and 6B are attributed to COL2-specific T cells and not driven by nonspecific infection tolerance, we analyzed PD-1 expression in non-COL2 T cells (tetramer-negative fraction) using flow cytometry (Figure S9A).

In addition, in DR4-AL179 + isotype-treated mice, T cells displayed a downregulation of proteins indicating reduced T cell activation^38^^,^^39^^,^^40^ but an upregulation of proteins involved in regulatory pathways^41^^,^^42^^,^^43^^,^^44^ (Figures 6A and 6B). We validated the reduced expression of CD48 and THY1, proteins associated with T cell activation, by flow cytometry on COL2-specific T cells in DR4-AL179-vaccinated mice 15 DPI (Figure 6C). Notably, comparing the CD4+ T cell proteomic landscape between DR4-AL179 + isotype and DR4-AL179 + anti-VISTA-treated mice (Figures S9B and S9C) showed that VISTA blockage resulted in a proteomic profile similar to the control group (DR4-CLIP). Gene Ontology (GO) enrichment analysis of differentially expressed proteins from the proteomics data on CD4 T cells revealed that VISTA plays a significant role in most identified pathways (Figure S9D).

To understand whether transferred T cells affect B cell activation, we conducted mass spectrometry-based proteomics on FACS-sorted B cells from DR4-AL179 isotype-treated mice compared to controls. B cells in DR4-AL179 isotype-treated mice showed a downregulation of proteins related to B cell activation,^45^^,^^46^^,^^47^ migration,^48^^,^^49^ and antibody response, particularly IgG, IgM, and IgG1 isotypes. Conversely, proteins associated with tissue damage resolution in inflammatory arthritis^50^ and negative regulation of nuclear factor (NF)-κB activation^51^ were upregulated in DR4-AL179 isotype-treated mice compared to controls (Figures 6D and 6E). We validated the reduced secretion of anti-COL2 IgG and anti-COL2 IgG1 by ELISA from serum at different time points during the transfer experiment (Figures 6F and 6G). Reduced expression of CXCR5 was confirmed by flow cytometry in DR4-AL179-vaccinated mice 15 DPI compared to DR4-CLIP-vaccinated mice (Figure 6H). Additionally, proteins involved in B cell migration, such as ALCAM (CD166)^52^ and C-C chemokine receptor type 7 (CCR7),^53^ were also downregulated in DR4-AL179-vaccinated mice 15 DPI (Figure 6I). Finally, differences in the B cell proteomic landscape between DR4-AL179 + anti-VISTA and DR4-AL179 + isotype control mice (Figures S9E and S9F) showed upregulation of proteins related to B cell activation and migration, similar to the differences observed between DR4-AL179 + isotype-treated mice and the control group.

## Discussion

We have developed a novel tolerogenic vaccine for the prevention of autoimmune arthritis. Unlike previous formulations, this new vaccine does not require galactosylation, a key structure that was crucial for the efficacy of the described COL2_259-273_ peptide when bound to relevant MHCII molecules.^25^^,^^26^ The new vaccine is based on a chemical modification of the glycine at position 265 and it induces protection against arthritis through activation of VISTA-positive-non-conventional regulatory T cells, similar to our previously described galactosylated COL2_259-273_ vaccine in A^q^ mice.

Reactive T cells against the immunodominant COL2_259-273_ peptide are present in the peripheral blood and synovial fluid of RA patients.^19^^,^^20^^,^^54^ Similar to the A^q^ or DRB1∗04:01-expressing mice, most of these T cells recognize the hydroxylation or galactosylation of the lysine residues within the peptide.^55^ Interestingly, MHCII-COL2 complexes lacking galactosylation fail to reduce arthritis severity in mice, underscoring the importance of post-translational modifications in therapeutic efficacy.^25^ In this study, we present a novel vaccine comprising a complex of a chemically modified COL2_259-273_ peptide (AL179) with the RA-associated MHCII allele DRB1∗04:01. By modifying the glycine residue at position 265, a key P3 position for TCR recognition,^56^^,^^57^ we created a peptide that induced tolerance even without galactosylation. This substitution enhanced the peptide interaction with the TCR without compromising MHCII binding. These findings suggest that the tolerogenic capacity of naturally occurring regulatory peptides can be enhanced through targeted synthetic modifications.

To facilitate the translation of this vaccine to human clinical use, we evaluated its efficacy using our novel MHCII humanized mouse model. The model was specifically developed to overcome the artifacts observed in traditional MHCII transgenic mice, which arise due to defective or deficient expression of MHCII molecules. The vaccine was tested in DRB1∗04:01-expressing mice, which have been shown to physiologically express the human MHCII molecule and exhibited allele-restricted immune responses and arthritis association, consistent with human RA.^14^

One of the critical challenges in MHCII-peptide-based therapies is the rapid clearance of these complexes from the body. In previous studies, this issue was mitigated using osmotic pumps implanted s.c. for continuous delivery.^26^ In this study, we demonstrate that three s.c. administrations alone are equally effective, offering a more practical and scalable alternative to prepare the therapy for human application.

The AL179 peptide, when complexed with the RA-associated MHCII allele DRB1∗04:01, significantly alleviated autoimmune arthritis and induced a robust tolerogenic response, reducing COL2-specific T cells and activating regulatory T cell populations. The therapeutic efficacy of our DR4-AL179 vaccine likely involves multiple complementary tolerogenic mechanisms. While our data strongly support the role of VISTA-expressing regulatory T cells, the observed upregulation of PD-1 and increase in CD73^high^FR4^high^ populations among COL2-specific T cells suggest potential contributions from T cell exhaustion and anergy pathways. These mechanisms may work in concert to establish robust immune tolerance, with VISTA enrichment serving as a key component of this multi-faceted response.

In our previous work, we hypothesized that VISTA plays a crucial role in vaccination efficacy in A^q^ mice by performing some *ex vivo* experiments.^26^ Building on this finding, we confirmed its significant contribution through an adoptive T cell-transfer experiment. Our study demonstrated that blocking VISTA substantially impaired the capacity of transferred T cells to ameliorate autoimmune arthritis, highlighting it as a crucial pathway in the vaccine’s mechanism of action. VISTA possesses distinct characteristics as a potent negative regulator of T cell function.^58^^,^^59^ Its role as an immune checkpoint is highlighted by observations that *Vsir*-knockout mice exhibit exacerbated T cell-mediated immune pathology across various disease models, including graft-versus-host disease (GVHD), experimental autoimmune encephalomyelitis (EAE), and RA,^36^^,^^60^ and developed spontaneous autoimmunity resembling systemic lupus erythematosus (SLE).^61^ Consequently, VISTA deficiency led to the breakdown of self-tolerance and the emergence of inflammatory T cell responses.^30^ Therefore, engaging VISTA post vaccination enhances an immunosuppressive phenotype crucial for augmenting T cell tolerance. This VISTA-mediated immunosuppressive phenotype may work synergistically with other tolerance mechanisms, including the induction of T cell anergy and deletion of autoreactive cells, to establish robust and lasting protection against autoimmune responses. Interestingly, the identification and characterization of this phenotype could allow us to follow and monitor the vaccination effect in humans. In conclusion, our study demonstrates that the DR4-AL179 vaccine, with a unique synthetic modification of the non-glycosylated COL2 peptide, represents a promising therapeutic approach for treating autoimmune arthritis. The vaccine’s ability to induce tolerance through multiple mechanisms, particularly the enrichment of VISTA-expressing regulatory T cells, suggests its potential for robust therapeutic efficacy. These findings suggest that precise synthetic modifications can augment the tolerogenic capacity of naturally occurring regulatory peptides. Since galactosylation of the peptide is no longer required, scaling up production for human therapy becomes more feasible, as it eliminates the challenges associated with ensuring the correct degree of galactosylation and purity. By deepening our knowledge of this strategy, we aim to develop more effective therapeutic approaches, potentially resulting in better patient outcomes for autoimmune diseases.

## Materials and Methods

### Mice

Humanized mice (B6N.DRA.DRB1∗04:01.hIi.*Cia9i*) with a knockin of the human MHCII allele DRB1∗04:01 (Parker, in short), were generated as previously described,^14^ and founders were provided by Vacara (Vacara.se)

In our experiments, 8- to 10-week-old male and female mice were used for phenotype and DTH experiments, while 12- to 14-week-old male and female mice were used for the CIA model. All experiments were performed with age- and sex-matched mice in a blinded fashion. Mice were kept under specific-pathogen-free (SPF) conditions in the animal facility of the Section for Medical Inflammation Research, Karolinska Institute, Stockholm, Sweden. Animals were housed in individually ventilated cages with wood shavings in a climate-controlled environment with a 14-h light-dark cycle and provided with standard chow and water *ad libitum*. All mice were healthy, with no effects on basic physiological parameters. The sample size for each experiment was determined following the reduce, refine, replace (3R) principles framework, ensuring the minimum required for statistical power while prioritizing ethical considerations. Experimental procedures were approved by the ethical committees in Stockholm, Sweden. Ethical permit numbers were 12923/18 and N134/13 (genotyping and serotyping), N35/16, and 2660/2021 (DTH, CIA).

### Synthesis of COL2-peptides with and without glycosylation

The 10 glycopeptides and their counterparts without glycosylation were designed based on previous results^57^ and synthesized and purified following the solid-phase methodology described elsewhere.^57^^,^^62^ The purity of the glycopeptides and their counterparts was determined to be >95% according to analytical reversed-phase high-performance liquid chromatography (HPLC), and their identities were confirmed by MALDI-TOF mass spectrometry.

### Vaccine constructs synthesis and purification

The sequence of the DRB1∗04:01/hCLIPmut construct is shown in Figure S10. The native leader sequences of the alpha and beta chains were replaced by a *Drosophila* BiP protein signal sequence. The extracellular domains of DRB1∗04:01 were truncated and the transmembrane regions were replaced by an acidic and basic zipper dimerization motif, respectively. At the C terminus of the alpha chain, a 15-amino acid AviTag peptide for site-specific biotinylation was inserted after the basic zipper, whereas a polyhistidine tag was inserted after the acidic zipper at the C terminus of the beta chain. For the DRB1∗04:01/hCLIPmut construct, a low-affinity peptide hCLIPmut (PVSKARMATGALAQA) followed by a thrombin cleavage site was added to the N terminus of the beta chain.

The genes were synthesized at Eurofins with KpnI and XhoI restriction sites at the 5′ and 3′ ends. The synthesized genes were restriction enzymes digested using FastDigest enzymes (Thermo Fisher Scientific). The digested DNA fragments were cloned into a mammalian expression vector pcDNA3.4 (Life Technologies) that was digested using the same restriction enzymes. After sequence verification, the recombinant plasmids were co-transfected into Expi293F cells (Life Technologies) with FectoPRO DNA transfection reagent (Polyplus transfection). The supernatants were harvested 6 days post transfection. The recombinant protein was first captured using a 5-mL HisTrap Excel (Cytiva) affinity column followed by size-exclusion chromatography on Superdex 200 pg (Cytiva). The recombinant protein was purified as a single peak, concentrated and diafiltrated into PBS to be used as an active vaccine component.

For peptide replacement, thrombin (Novagen, 1 unit of thrombin to 1 mg of purified protein) and the desired COL2 peptide in excess was added to replace the covalently linked hCLIPmut peptide. The reaction mixture was incubated at ambient temperature overnight with slow end-to-end rotation followed by incubation at 4°C for 2 days. Cleaved peptide and additional excess desired peptide were removed by size-exclusion chromatography on a Superdex 200 pg column.

### Peptide competitive-binding assay

For peptide competitive-binding assay, a 0.2 μM solution of purified DRB1∗04:01-hCLIPmut complex and 6 μM Biotin-hCLIP peptide was incubated at room temperature (RT) for 48 h. Various concentrations of COL2 peptides and influenza hemagglutinin peptide (HA_306-318_) as a positive control were added as competitors to Biotin-Ahx-hCLIP peptide. Bound peptides were separated from free peptides by immobilizing the DRB1∗04:01 molecules on microtiter plates coated with the anti-human HLA-DR (L243, in-house) antibody and subsequently washed with PBS. The L243 antibody was adhered to the plate by an overnight incubation of a 2 μg/mL solution at 4°C. Bound biotinylated peptides were detected by incubations with streptavidin-europium followed by a chelating enhancement solution. Fluorescence was quantitated using a Synergy 2 plate reader (BioTek Instrument), and data are expressed as relative fluorescence units (RFU) measured. Each binding assay was performed in duplicate, and the data are representative of three experiments.

### *In vitro* evaluation of T cell activation by glycopeptides

Spleen cells (APCs) prepared from naive Parker mice were resuspended in complete Dulbecco’s modified Eagle’s medium (DMEM) containing DMEM + GlutaMAX (Gibco), 5% heat-inactivated fetal bovine serum (FBS) (Gibco), 10 μM HEPES (Sigma), 100 μg/mL streptomycin sulfate (Sigma), 100 IU/mL penicillin C (Sigma), and 50 μM β-mercaptoethanol (Gibco). Splenocytes (5 × 10^5^) were co-cultured with three different clones (mDR1.1, mDR17.2, and hDR11.2) of DR4-restricted T cell hybridoma cells specific for the galactosylated COL2_259-273_ epitope (5 × 10^4^) in a total volume of 200 μL in U-bottom 96-well plates. The different glycopeptides were titrated (0.15–37.5 μM), while the amounts of T-cell hybridoma and APCs were kept constant. As negative controls, T-cell hybridomas incubated with APCs in only medium were used. After 24 h cultured at 37°C, supernatants were collected to assess antigen presentation by measuring the IL-2 levels by ELISA. Establishment of T cell hybridoma clones and determination of antigen specificity was described previously.^19^

### *In vitro* evaluation of T cell activation by MHCII-peptide complexes

Stimulation experiments of T-hybridoma cells were performed in 96-well plates with precoating of MHCII-peptide complexes at different concentrations of between 10 and 100 μg/mL in PBS overnight. The coated wells were then washed twice with sterile PBS. Subsequently, 1 × 10^5^ murine T-hybridoma cells (3H8) specifically recognizing native COL2 in a DRB1∗04:01-restricted manner were added in 200 μL of DMEM supplemented with 5% fetal calf serum (FCS), 100 IU/mL penicillin, and 100 μg/mL streptomycin. T cell activation by the MHCII-peptide complexes was assessed by IL-2 release into the medium (Sandwich IL-2 ELISA, BioLegend).

### IL-2 ELISA

Flat 96-well plates (Maxisorp, Nunc) were coated overnight at 4°C with the IL-2 capture antibody (5 μg/mL JES6-IA12, in-house produced) in PBS. Supernatants from cell culture were added and plates were incubated for 2 h at RT before washing (PBS-Tween 0.05%) and adding the biotinylated detection antibody (2 μg/mL JES6-5H4, in-house produced) for 1 h at RT. Plates were washed and incubated for 30 min at RT with Eu-labeled streptavidin (PerkinElmer; 1:1,000) in buffer E (50 μM Tris-HCl, 0.9% [wt/vol] NaCl, 0.5% [wt/vol] BSA, 0.1% Tween 20, 2 mM EDTA). After washing, DELFIA Enhancement Solution (PerkinElmer) was added, and fluorescence was read at 620 nm (Synergy 2, BioTek).

### Vaccination protocol

DR4-peptide complexes (100 μg) were administered using one of two methods: via micro-osmotic pumps (DURECT Corporation; ALZET Model 2001D, delivering 8 μL/h for a 1-day duration, reservoir capacity 200 μL) or through three s.c. injections administered every 4 h. For the placement of s.c. pumps, a minor incision was made to create an s.c. pocket where the micro-pumps were inserted with the flow moderator oriented away from the incision site. Post-surgical analgesia was provided by s.c. injection of buprenorphine (e.g., Temgesic; Indivior Europe) at a dosage of 0.2 mg/kg. Mice received vaccinations on both day 4 and day 32 post immunization.

### DTH

Parker mice were sensitized by intradermal (i.d.) injection of 100 μg of bovine COL2^63^ in 100 μL of a 1:1 emulsion with complete Freund’s adjuvant (CFA; BD, Difco) and 10 mM acetic acid at the base of the tail. Day 8 after sensitization, the right ear was injected i.d. with 10 μL of COL2 in acetic acid (1 mg/mL) while the control left ear was injected with 10 μL of acetic acid in PBS. Ear swelling response was measured during 0, 24, 48, and 72 h after the challenge using a caliper. Ear swelling was calculated by subtracting the thickness of the acetic acid-injected ear from the thickness of the COL2-injected ear normalized to day 0 thickness.^64^

### CIA

Parker mice were immunized with 100 μg of bovine COL2 in 100 μL of a 1:1 emulsion with CFA and 10 mM acetic acid (mixed by an emulsifier machine, BTB POWER-Kit)^65^ injected i.d. at the base of the tail. Mice were challenged on day 35 with 50 μg of COL2 in 50 μL of incomplete Freund’s adjuvant (IFA) (BD, Difco) emulsion injected i.d. The progression of arthritis was assessed by visually inspecting the paws employing a macroscopic scoring system. In this scoring system (0–58), visibly inflamed ankles or wrists were assigned five points each, while inflamed toes or fingers were accorded one point each. During the CIA disease course, blood samples were collected through the cheek-bleeding technique at different time points.

### Adoptive T cell transfer

The transfer experiment depicted in Figure 3I was conducted using DR4-CLIP- and DR4-AL179-treated Parker donor male mice, which were sacrificed 15 DPI. Untouched CD4+ T cells were isolated from the spleens and lymph nodes using magnetic-activated cell sorting (MACS) in accordance with the manufacturer’s instructions (Miltenyi Biotec, catalog no. 130-104-45). A total of 5 × 10^6^ CD4+ T cells were adoptively transferred intravenously (i.v.) into COL2-immunized Parker mice 4 DPI. The transfer experiment shown in Figures 5C–5F, included a secondary step where CD4+ T cells from DR4-AL179-vaccinated donor mice were divided into two groups. These cells were incubated for 1.5 h with either 100 μg/mL of neutralizing anti-VISTA antibody (clone 13F3) or an anti-isotype control (all from Bio X Cell) before being transferred i.v. into COL2-immunized Parker mice 4 days post immunization.

The purity of the CD4+ T cells, as determined by flow cytometry, was greater than 85%.

### ELISA and bead-based multiplex flow immunoassay for determination of anti-COL2 antibody titers and epitope specificity

Serum samples were used for the analysis of the COL2-specific IgG antibody response by ELISA previously described in Romero-Castillo et al.^14^ or for the analysis of epitope specificity of the IgG antibody response using peptides derived from COL2 assayed on multiplex bead-based flow immunoassay on the Luminex platform, as described previously.^66^

### Detection of anti-COL2 U1 epitope IgG antibodies by ELISA

Flat 96-well plates (Maxisorp, Nunc) were coated with Neutravidin (Thermo Fisher Scientific; 5 μg/mL in PBS) overnight at 4°C. The following day, plates were washed with PBS containing 0.05% Tween 20 and incubated with biotin-conjugated triple-helical COL2 peptide containing the U1 epitope (1 μg/mL in PBS)^67^ for 30 min at RT. After blocking with 1% BSA in PBS for 1 h at RT, diluted serum samples (1:2,000) were added and incubated for 2 h at RT. Plates were then washed with PBS-Tween 0.05% and incubated with HRP-conjugated anti-mouse IgG detection antibody (1:4,000 dilution; Southern Biotech) for 1 h at RT. Following a final wash step, 50 μL of 3,3′,5,5′-tetramethylbenzidine (TMB) substrate (Sigma) was added to each well. The reaction was stopped by adding 50 μL of 0.3 M H_2_SO_4_, and absorbance was measured at 450 nm using a Synergy 2 plate reader (BioTek).

### Cytokine and chemokine assay

Serum cytokines and chemokines within the CIA model were detected 15 days post immunization using a Mouse Cytokine Array/Chemokine Array 44-Plex (Eve Technologies, Calgary, AB, Canada).

### Preparation of cell suspension from lymphoid organs

Spleens or inguinal lymph nodes were mashed using a 1-mL syringe plunger on a 40-μm cell strainer (Falcon). The cell suspension was washed once in PBS and centrifuged at 350 × *g* for 5 min at RT. For spleen, red blood cells (RBCs) were lysed in 1 mL of RBC lysis buffer for 1–2 min at RT (0.155 M NH_4_Cl, 120 mM NaHCO_3_, 100 μM EDTA). Thereafter, cells were washed in PBS and carefully transferred to a new 15-mL tube to get rid of debris. Cells were centrifuged and resuspended in 1–3 mL of DMEM (Gibco, Invitrogen) for counting in a Sysmex KX-21N cell counter.

### *Ex vivo* T cell-suppression assays

Flat-bottom 96-well plates were pre-coated with anti-CD3 antibody (2 μg/mL, 500A2, BD Pharmingen) in PBS overnight at 4°C. CD4+ T cells were isolated from the spleens and lymph nodes of DR4-CLIP, DR4-AL179-vaccinated Parker mice and naive Parker mice using negative selection (Dynal Mouse CD4 Negative Isolation Kit, Thermo Fisher Scientific). The cells were resuspended in complete RPMI 1640 with GlutaMAX (Thermo Scientific) containing 50 μg/mL streptomycin sulfate (Sigma), 60 μg/mL penicillin C (Sigma), 10% heat-inactivated FBS (Thermo Scientific), 50 μM β-mercaptoethanol (Thermo Scientific), and 10 μM HEPES (Sigma).

The isolated CD4+ T cells from naive Parker mice were washed and labeled with the fluorescent dye CellTrace violet (Invitrogen) according to the manufacturer’s protocol. Violet-labeled CD4+ T cells (1 × 10^5^) from naive mice and 3 × 10^5^ CD4+ T cells from treated mice were added to the pre-coated plates. The soluble form of hamster anti-mouse CD28 antibody (0.5 μg/mL, 37.51, BD) was also added to each well. Cells were incubated at 37°C in a 5% CO_2_ atmosphere.

To investigate the role of immune checkpoint molecules in vaccine suppression, we blocked specific molecules by incubating CD4+ T cells from vaccinated mice for 1.5 h with 100 μg/mL of neutralizing anti-VISTA (clone 13F3), anti-PD-1 (clone RMP1-14), or an isotype control antibody (all from Bio X Cell) before being co-cultured with violet-labeled CD4+ T from naive mice. The dilution of CellTrace in responder cells was assessed 72 h later using flow cytometry.

### ELISpot

For T cell recall assays, 1 × 10^6^ lymph node cells per/well were plated in anti-IL17A (TC11-18H10.1, 5 μL, Mabtech)-coated plates (Merck Millipore, #MSIPS4W10), stimulated with denatured bCOL2 (30 μg/mL), and incubated for 48 h at 37°C. Positive controls consisted of lymph node cells stimulated with Concanavalin A (ConA; 1 μg/mL, Sigma), while negative controls comprised unstimulated cells in complete medium only. Bound IL-17A was detected with anti-IL17A (TC11-8H4, 1 μg/mL, Mabtech) followed by streptavidin-conjugated alkaline phosphatase. The spots were developed using the substrate BCIP/Nitroblue Tetrazolium (Sigma). Scanned wells (ImmunoScan) were analyzed with ImmunoSpot software (Cellular Technology).

### Synthesis of DRB1∗04:01-COL2_259-273_ tetramer complexes for flow cytometry

For preparation of the DRB1∗04:01-COL2_259-273_ tetramer complexes, the proteins were transferred to a biotinylation buffer (20 mM Tris-HCl, 50 mM NaCl, pH 8.0) using a centrifuge device with molecular weight cut off (MWCO) of 10 kDa (Cytiva AB). Biotinylation using biotin-protein ligase was performed according to the manufacturer’s instructions (Avidity, Denver, CO) and the reaction was carried out at 30°C for 2 h. Following biotinylation, thrombin (Novagen, 1 unit of thrombin to 1 mg of purified protein) and the COL2_259-273_ peptide in excess were added to replace the covalently linked hCLIPmut peptide. The reaction mixture was incubated at ambient temperature overnight with slow end-to-end rotation followed by incubation at 4°C for 2 days. Free biotin, cleaved peptide, and additional excess desired peptide were removed by size-exclusion chromatography on a Superdex 200 pg column.

### Flow cytometry and staining and enrichment of DR4-COL2_259-273_ specific cells

Single-cell suspensions from spleens and inguinal lymph nodes were obtained as previously described above. All centrifugation steps were carried out at 350 × *g* for 5 min at RT. To block Fc receptors, the cells were incubated in 25 μL of FACS buffer (2% FCS in PBS) containing 10 μg of anti-CD16/CD32 monoclonal antibody (mAb) (2.4G2; in-house produced) in 96-well plates for 15 min at 4°C. Samples were washed with 150 μL of PBS and subsequently stained with the indicated antibodies diluted 1:200 or 1:300 in 50 μL of FACS buffer at 4°C for 20 min in the dark. Cells were washed once, fixed, and permeabilized for intracellular staining using BD Cytofix/Cytoperm (BD Biosciences) according to the manufacturer's instructions and stained with antibodies diluted at 1:200 in 50 μL of permeabilization buffer (BD Biosciences) for 30 min at RT.

DR4-COL2_259-273_ tetramer was prepared by mixing the biotinylated MHCII-peptide complexes with streptavidin R-phycoerythrin (Thermo Fisher Scientific, Eugene, OR, USA) at 4:1 molar ratio.^68^ To detect DR4-COL2_259-273_-specific cells, splenocytes and inguinal lymph node cells were first incubated with an anti-CD16/CD32 mAb and 50 nM dasatinib (Santa Cruz Biotechnology, Santa Cruz, CA, USA) for 30 min at 37°C. Then the cells were stained with DR4-COL2_259-273_ tetramer (20 μg/mL) in DMEM containing 5% FBS (Gibco, Invitrogen) in the presence of 50 nM dasatinib for 1 h at 37°C. Enrichment of DR4-COL2_259-273_ tetramer-specific cells was performed using anti-R-Phycoerythrin magnetic beads (Miltenyi Biotec) according to the previously described protocol.^69^ Subsequent surface and intranuclear staining were carried out as indicated above.

### Antibodies

All the antibodies used in this study were purchased from BD Bioscience, BioLegend, Miltenyi, or Invitrogen. Anti-mouse antibodies and clones used were the following: anti-CD4 (RM4-5), anti-CD44 (IM7), anti-PD-1 (RMP1-30), anti-FR4 (eBio12A5), anti-CD73 (eBioTY/11.8), anti-VISTA (MIH64), anti-FOXP3 (FJK-16s), anti-CD49 (DX5), anti-LAG-3 (C9B7W), anti-B220 (RA3-6B2), anti-CD11b (M1/70), anti-IA/IE (M5/114.15.2), anti-CD19 (6D5), anti-CD48 (HM48-1), anti-THY1 (S20007C), anti-NRP1 (3E12), anti-CCR7 (4B12), anti-ALCAM (RRA270), anti-4RA (I015F8), anti-CXCR5 (L138D7), anti-TLR9 (S18025A), and anti-TLR7 (A94B10). Anti-human HLA-DR (L243) was also used. Live/dead fixable dyes for discrimination of dead cells were purchased from Thermo Fisher and three different formats were used: green, near-infrared, and violet.

Samples were acquired with Attune NxT flow cytometer (Thermo Fisher Scientific) and analyzed with FlowJo version 10.7.2.

### Histology

At the end of the CIA and transfer experiments, mice were euthanized, and their skinless hind paws were collected. The hind paws were fixed in 10% phosphate-buffered formaldehyde for 15 days. Following fixation, the tissues were decalcified for 4–5 weeks in a decalcification buffer consisting of 10% EDTA, 7.5% polyvinylpyrrolidone, and 0.1 M Tris-HCl (pH 6.95).

After decalcification, the tissues were dehydrated in 70% ethanol and then embedded in paraffin. Tissue sections were subsequently stained with hematoxylin and eosin (H&E). The sectioning and H&E staining were performed by Histocore at the Karolinska Institutet, Sweden.

### Proteomic sample preparation

At the terminal point of the transfer experiment, where CD4+ T cells from DR4-AL179 treated mice were incubated with either anti-VISTA or an isotype control before transfer into COL2-immunized recipient mice, both spleens and draining lymph nodes (dLNs) were subjected to FACS of both CD4+ T and B cells. Following FACS, the sorted cells were pelleted and stored at −20°C until proteomic sample preparation. Subsequently, sorted T and B cells were resuspended in 50 mM Tris pH 8.5 and 1% sodium deoxycholate, and the proteins were denatured at 95°C for 10 min. Subsequently, the protein amount was determined by bicinchoninic acid assay (BCA) (Thermo Fisher Scientific) following the manufacturer’s protocol. Individual samples were reduced with 5 mM DTT for 1 h followed by alkylation with 15 mM iodoacetamide in the dark for another hour. Samples were then digested with LysC endopeptidase (an enzyme-to-protein ratio of 1:100 w/w) for 2 h, followed by digestion using the same amount of trypsin overnight. Samples were desalted by StageTip (Thermo Fisher Scientific) following the manufacturer’s protocol. Cleaned samples were dried in a speed vac and stored at −80°C.

### Mass spectrometry analysis

Samples were resuspended in buffer A (2% ACN, 0.1% FA) and injected into an UltiMate 3000 UPLC autosampler (Thermo Fisher Scientific), which was connected to an Orbitrap Fusion Lumos Tribrid mass spectrometer (Thermo Fisher Scientific). The peptides were loaded on a trap column (Acclaim PepMap 100 C18, 100 μm × 2 cm) and subsequently separated on a 50-cm-long C18 Easy spray column (Thermo Fisher Scientific). Chromatographic separation of the peptides was achieved by the following 105-min gradient: 4%–20% of solvent B (98% ACN and 0.1% FA) in 80 min, to 26% in 10 min, to 95% in 3 min where it was kept for another 3 min, before going to 4% in 1 min and held for 8 min. During the entire gradient, the mass spectrometer was operating in positive polarity. All mass spectra were acquired in profile mode using the Orbitrap analyzer. An acquisition cycle consisted of one survey mass spectrum acquired at a mass resolution of 120,000 from *m/z* 395 to 1,005, with an automated gain control (AGC) target of 1,000,000 charges and a maximal injection time of 150 ms. This was followed by 40 data independent acquisition (DIA) MS/MS events with variable isolation windows. The individual mass spectra were recorded at a resolution of 30,000 with 28% NCE; stepped normalized collision energy (NCE) was set as 2%, and maximal injection time was 54 ms. A library of MS/MS spectra was created with a pooled sample. The data acquisition was the same as for the individual samples with the exception that the DIA MS/MS spectra were recorded over a mass range of 120 *m/z* at a resolution of 60,000 with a maximum injection time of 54 ms and an overlap between MS/MS windows of 4 *m/z* units.

### Bioinformatics and proteomic data analysis

Acquired.raw files were converted to mzML format by MSConvert (version 3.0.21258)^70^ applying peak picking in the mass spectra with the vendor-provided algorithm (Thermo Fisher Scientific). The database search was performed in FragPipe (v18.0) as described in Yu et al.^71^ using the mouse Swissprot database (17,248 entries). Trypsin with up to one missed cleavage was set as a digestion enzyme, and oxidation of methionine and acetylation of the N terminus were set as variable modifications. Carbamidomethylation of cysteine residues was set as a fixed modification. Peptide length was restricted to 7–50 amino acids, and molecular mass from 500 to 5000 Da. The resulting peptide-spectrum matches were adjusted to a 1% false discovery rate with Percolator^72^ as part of the Philosopher toolkit (v4.4)^73^ and converted to an MS/MS spectra library. DIA files were analyzed by DIA-NN 1.8.1.^74^

All further data processing was performed in R (version 4.2.2). Known contaminants and decoy proteins were excluded from further analysis. Only proteins quantified in three out of the five replicates in at least one sample group were considered. Protein abundances were normalized by variance stabilization normalization approach and missing values at random were imputed by k-nearest neighbor method, whereas missing values not at random were imputed by drawing values from a downshifted normal distribution.^75^ All statistical comparisons were performed based on a two-tailed Student’s t test with equal variances; the differences between the comparisons were reported as log2-scaled fold changes.

## Data availability

The data supporting the findings of this study are available within the paper (supplemental information). The mass spectrometry proteomics data have been deposited to the ProteomeXchange Consortium (http://proteomecentral.proteomexchange.org) via the PRIDE partner repository^76^ with the dataset identifier PXD056818. All other data and the unique reagents used in this study are available from the corresponding author upon reasonable request.

## Acknowledgments

This work was supported by grants from 10.13039/501100004359Vetenskapsrådet (2024-02575), 10.13039/501100004191Novo Nordisk Fund (NNF24OC0090035), the 10.13039/501100004063Knut and Alice Wallenberg Foundation (2019.0059), and the Swedish Association against Rheumatism. L.R.-C. received support from the 10.13039/501100004047Karolinska Institutet foundation for Rheumatology Research (2020-02541, 2021-01145, 2022-02857, 2023-02645, 2024-03851), the 10.13039/501100009806Ulla and Gustaf af Uggla Foundation (2021-01015), the 10.13039/100007435Åke Wiberg Stiftelse (M21-0148; M24-0077), the Lars Hiertas Minne Foundation (F02023-0284), the Reumatikerförbundet Association (R995628), and the Konung Gustaf V:s (SGI-2023-0993) and got the Margarita Salas grant from the Spanish Ministry of Universities through the 10.13039/501100000780European Union (NextGeneration EU). The humanized mice were provided by Vacara AB. We would like to thank the Histological Core Facility in Biomedicum, especially Emma Mondoc, for performing the histology shown in this manuscript. Finally, we would like to thank all KM-A technicians, especially Carlos Palestro and Martina Andersson, for taking care of the animals.

## Author contributions

All authors were involved in drafting the article or revising it critically for important intellectual content, and all authors approved the final version to be published.

L.R.-C., V.U., O.S., H.B., J.K., R.A.Z., J.K., A.L., and R.H., designed research. L.R.-C., R.K.P., B.X., C.M.B., A.O.-C., K.Z., C.S., Z.X., H.L., P.S., L.C., A.K., C.L., and S.H. performed research. L.R.-C., R.K.P., C.M.B., C.G., and S.H. analyzed data. L.R.-C. and R.H. wrote the paper.

## Declaration of interests

R.H. is the founder of Vacara AB. Z.X. and O.S. are employees of Vacara AB.

## Supplemental information

Document S1. Figures S1–S10 and supplemental materials and methods

Document S2. Article plus supplemental information
