# Supplementary material for: Tolerogenic antigen-specific vaccine induces VISTA-enriched regulatory T cells and protects against arthritis in DRB1∗04:01 mice
Source: Mol Ther. 2025 Apr 24;33(8):3528–45. doi: 10.1016/j.ymthe.2025.04.034 (PMC12461628; doi:10.1016/j.ymthe.2025.04.034)
Supplement: Supplementary file 1 [file mmc1.pdf]

YMTHE, Volume *33*

Supplemental Information

Tolerogenic antigen-specific vaccine induces VISTA-enriched regulatory T cells and protects against arthritis in DRB1*04:01 mice

Laura Romero-Castillo, Rajan Kumar Pandey, Bingze Xu, Christian M. Beusch, Ana Oliveira-Coelho, Kejsi Zeqiraj, Carolin Svensson, Zhongwei Xu, Huqiao Luo, Outi Sareila, Pierre Sabatier, Changrong Ge, Lei Cheng, Vilma Urbonaviciute, Alexander Krämer, Cecilia Lindgren, Sabrina Haag, Johan Viljanen, Roman A. Zubarev, Jan Kihlberg, Anna Linusson, Harald Burkhardt, and Rikard Holmdahl

FIGURES

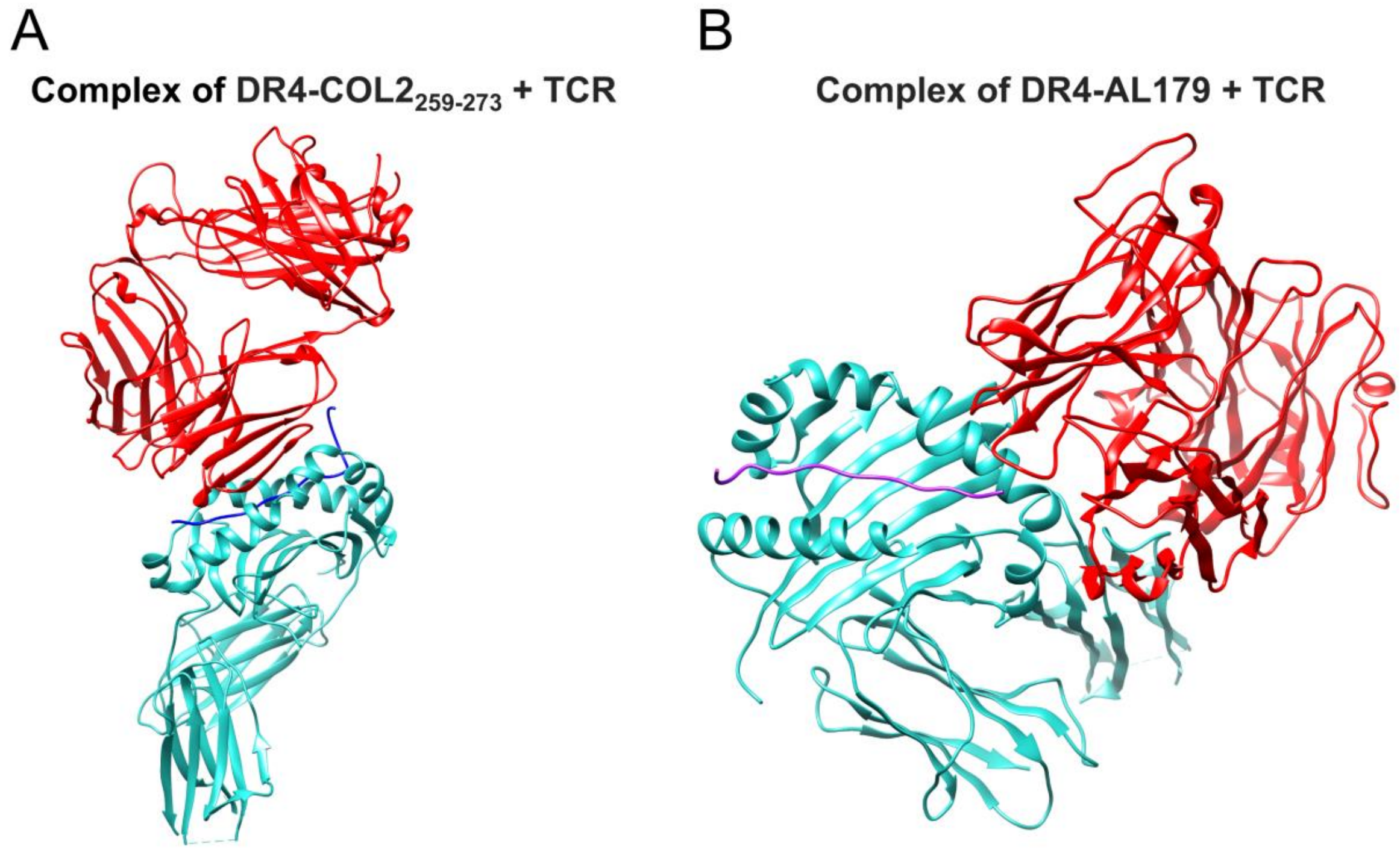

C

DR4-COL2$_{259-273}$ and TCR		DR4-AL179 and TCR	
COL2$_{259-273}$ and TCR	DR4-COL2$_{259-273}$ and TCR	AL179 and TCR	DR4-AL179 and TCR
Ala261-αSer52<br>Phe263-αThr54<br>Gly265- αThr54	βGln70-αSer61<br>αGlu55-αTyr49<br>αGln57-βGlu101	Ile2-βLeu97	βAla73-βPhe75<br>βThr77-βAsn31<br>αArg50-αThr54<br>αArg50-αLys55<br>βGlu87-βThr3<br>βTyr83-αTyr49<br>βTyr96-αSer78<br>βSer179-αGln79<br>βThr181-αGlu16<br>βThr181-αLys170<br>βLys184-αMet16

Fig. S1. Molecular docking analysis of protein-protein interactions between MHC class II-peptide complexes and T cell receptor. The crystal structures of DRB1*04:01 and T-cell receptor were retrieved from the Protein Data Bank (PDB). PyMOL mutagenesis tool was used to mutate the amino acid residues and create DRB1*04:01-COL2$_{259-273}$ and DRB1*04:01-AL179. The HDOCK docking server was used for protein-protein docking and to determine the binding interactions. (**A**) Three-dimensional representation of the docked complex between DR4-COL2$_{259-273}$ (shown in cyan and blue, respectively) and TCR (PDB ID:4UDT; shown in red). (**B**)

Three-dimensional representation of the docked complex between DR4-AL179 (shown in cyan and purple, respectively) and TCR (shown in red). (**C**) Detailed analysis of molecular interactions showing the specific amino acid residues involved in hydrogen bond formation between different MHCII-peptide complexes and TCR. α and β denote the respective chains of either MHCII (left column) or TCR (right column). Interacting residues forming the H-bonds were determined using PyMOL. The higher number of hydrogen bonds observed in the DR4-AL179/TCR complex indicates stronger binding affinity compared to the DR4-COL2$_{259\text{-}273}$/TCR complex.

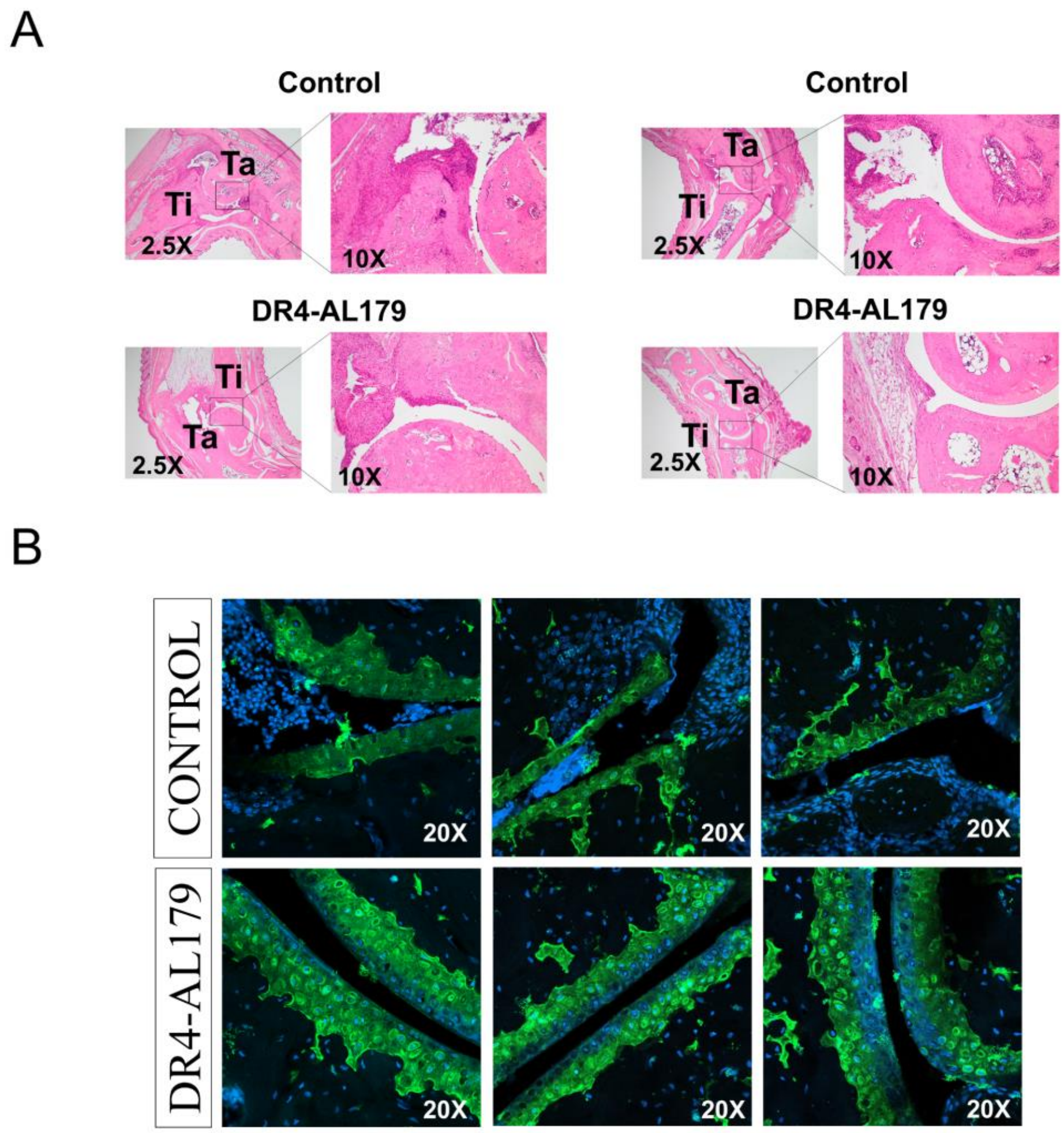

Fig. S2. Histological staining of joint tissues from DR4-CLIP (Control) and DR4-AL179 vaccinated mice in the CIA model corresponding to Figure 3E. (**A**) Hematoxylin and eosin (H&E) staining of the tibia (Ti) and talus (Ta). Control mice exhibited increased inflammatory cell infiltration and cartilage/bone erosion compared to DR4-AL179 vaccinated mice. (**B**) Immunofluorescence staining of COL2 in articular cartilage. DR4-AL179 vaccinated mice demonstrated enhanced preservation of COL2 fibrillar structure compared to the control group.

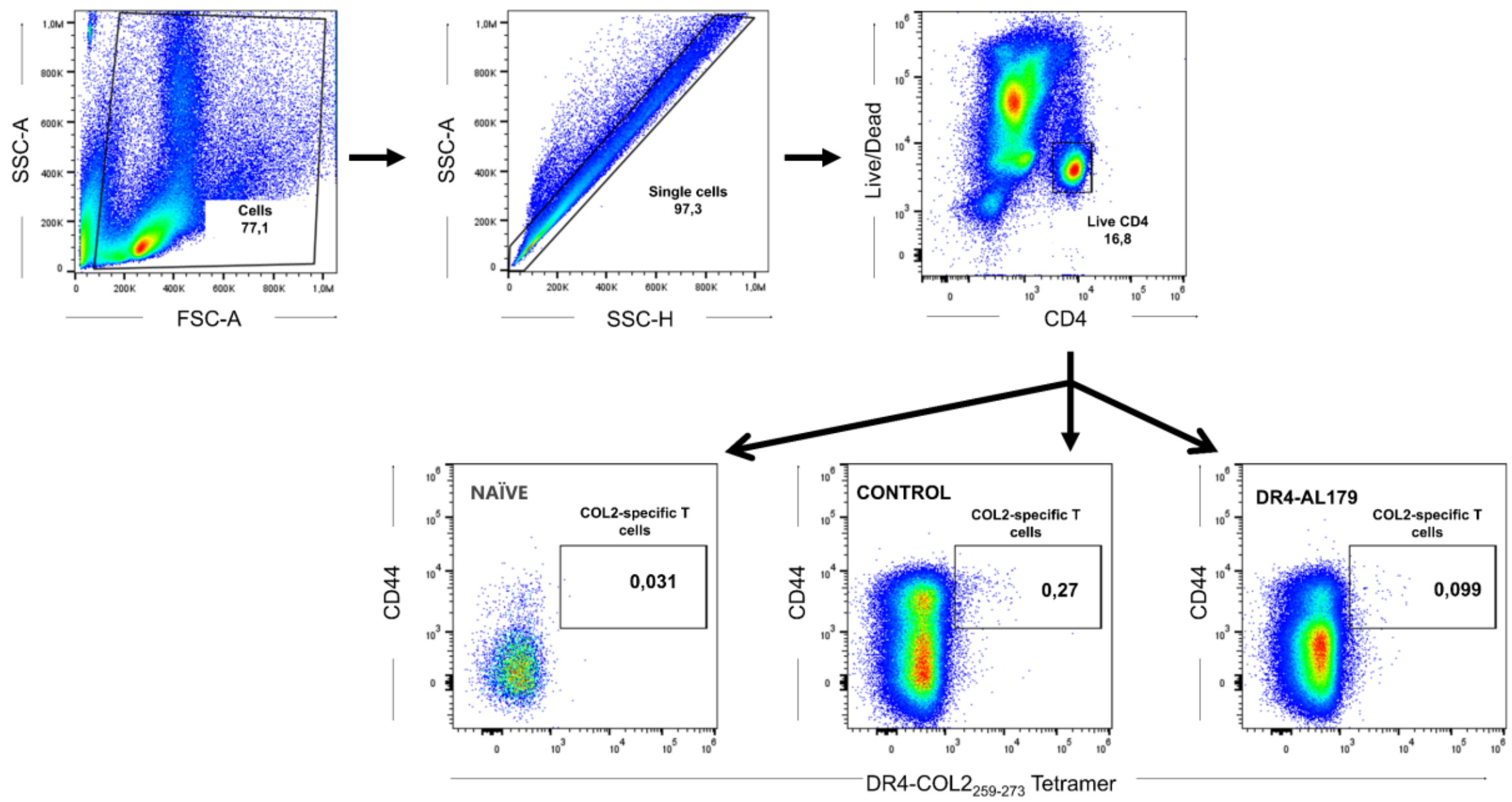

Fig. S3. Flow cytometry gating strategy for identification of COL2-specific T cells corresponding to Figure 3E. Representative gating scheme showing the sequential identification of COL2-specific T cells (CD44+ DR4-COL2$_{259\text{-}273}$ tetramer+) in spleen at day 80 of CIA model. Gates are shown for three experimental groups: mice vaccinated with DR4-CLIP (Control), mice vaccinated with DR4-AL179, and naïve (non-immunized) Parker mice. These gating strategies correspond to the data presented in Figure 3E.

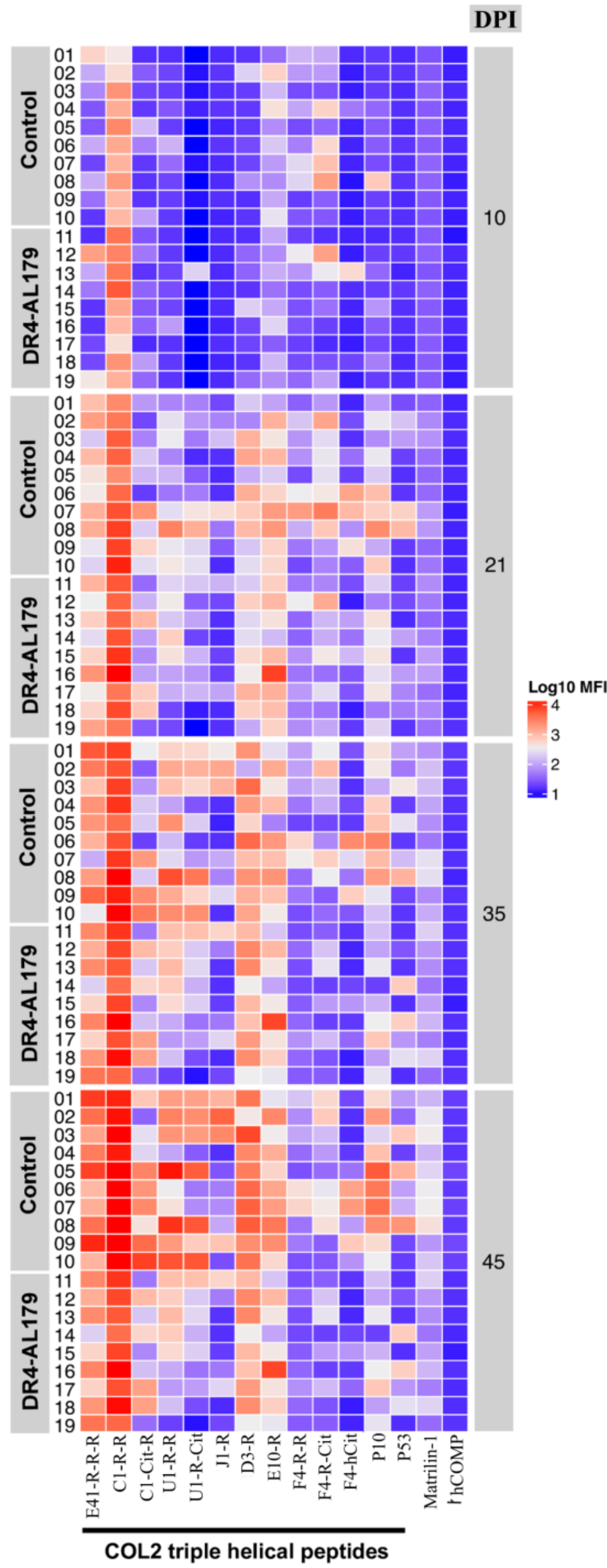

Fig. S4: Serum IgG antibody response to triple helical COL2 peptides in vaccinated mice. Parker mice were immunized with 100 µg of bCOL2 on day 0, treated with either DR4-CLIP (Control) or DR4-AL179 on days 4 and 32 postimmunization, and boosted on day 35 with 50 µg of bCOL2. Sera were collected at different days post immunization (DPI) during the CIA experiment. The antibody response to triple helical COL2 peptides or to matrilin-1 and hCOMP proteins (human cartilage oligomeric matrix protein) was detected by

bead-based multiplex flow immunoassay. Log10-transformed MFI values are presented as a heatmap. R: arginine. Cit: citrulline. hCit: homocitrulline. MFI: median fluorescence intensity.

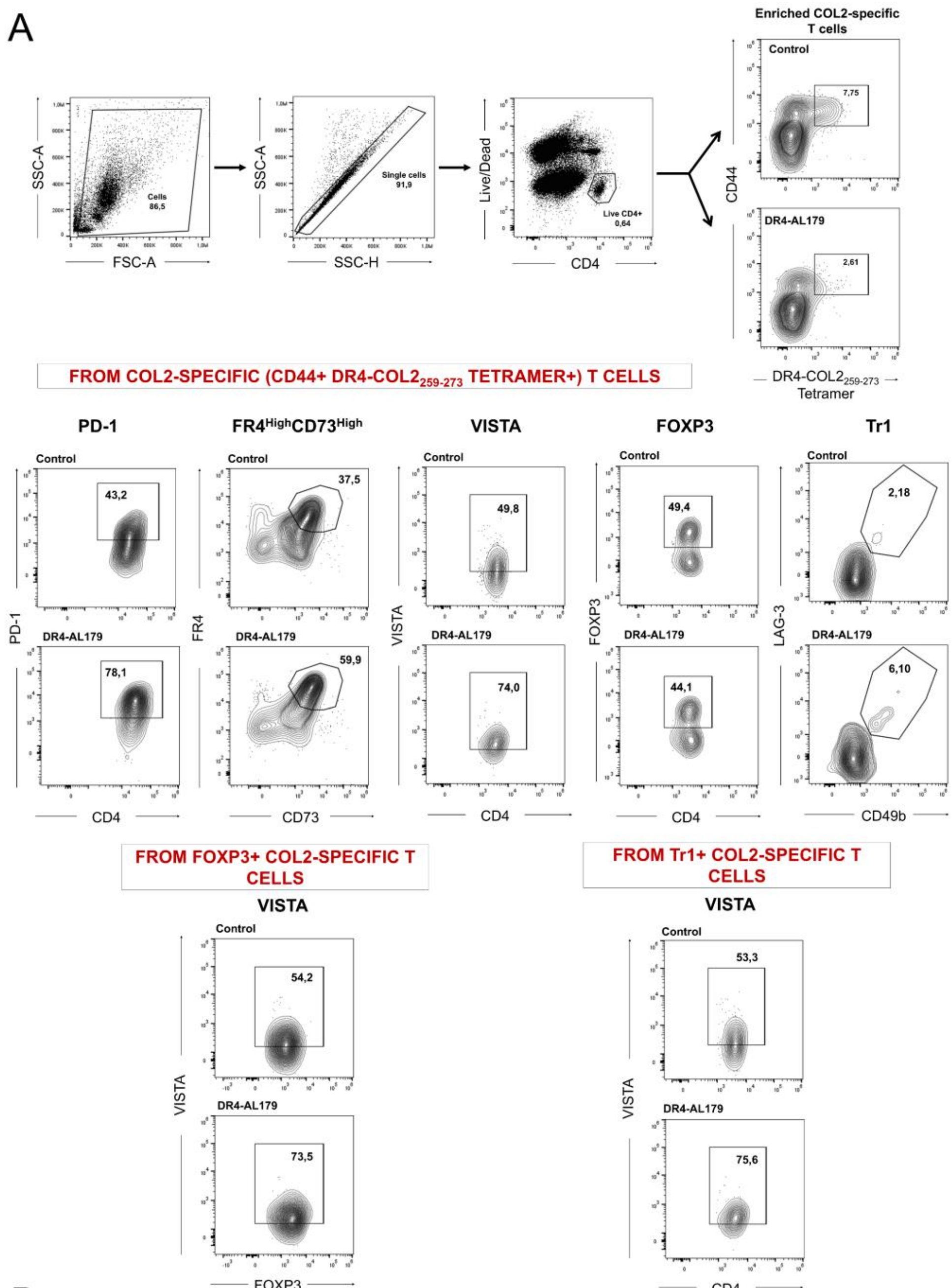

B

			Live CD4+ T cells	COL2-specific cells						
Mouse	Treatment group	Live CD4 (%)	COL2-specific T cells (%)	PD-1 (%)	CD73highFR4high T cells (%)	VISTA (%)	FOXP3 (%)	Tr1 (CD49+ LAG-3+) cells (%)	VISTA (%) in FOXP3+ cells	VISTA (%) in Tr1 cells
1	DR4-CLIP	0,64	12,9	58,9	37,5	42,7	49,7	7,52	37,2	51,5
2	DR4-CLIP	0,143	7,75	43,2	32,6	49,8	45,1	2,18	41,4	53,3
3	DR4-CLIP	0,81	6,76	42,5	26,5	54,7	58,4	0,9	48,4	59,3
4	DR4-CLIP	0,97	6,68	49	40,5	53	44,6	3,53	47,2	59,5
5	DR4-CLIP	1,24	7,27	59,8	26,6	58,7	46,7	2,78	52,1	62,6
6	DR4-AL179	0,62	4,98	78,1	50,9	60,9	54,7	28,2	53,9	64,9
7	DR4-AL179	0,67	2,97	75,1	49,3	68,9	44,1	6,1	69	70,7
8	DR4-AL179	1,22	1,43	82,1	57,4	74	38,8	8,2	69,9	75,6
9	DR4-AL179	1,03	2,61	83,5	49,4	60,8	44,3	8,1	56,4	64,2
10	DR4-AL179	0,45	4,42	80,4	54,5	75,6	23,3	11,1	70,3	77,2

Fig. S5. Flow cytometry gating strategy and quantitative analysis of immune cell markers corresponding to Figure 4. (**A**) Detailed gating strategy showing the analysis of the enriched COL2-specific T cells from

Control (DR4-CLIP) and DR4-AL179 vaccinated Parker mice. The sequential gating shows: lymphocytes from total cells, single cells, live CD4+ cells and COL2-specific T cells (defined by CD44+ DR4-COL2$_{259\text{-}273}$ tetramer). Within the COL2-specific T cell population, subsequent analyses show gating strategies for: PD-1+, FR4^{high}CD73^{high} T cells, VISTA+, FOXP3+ cells, and Tr1 cells. Additional analyses show VISTA+ on both FOXP3+ and Tr1 cells within the COL2-specific population. (**B**) Comprehensive table summarizing the percentages of each analyzed cell population per individual mouse as presented in Figure 4.

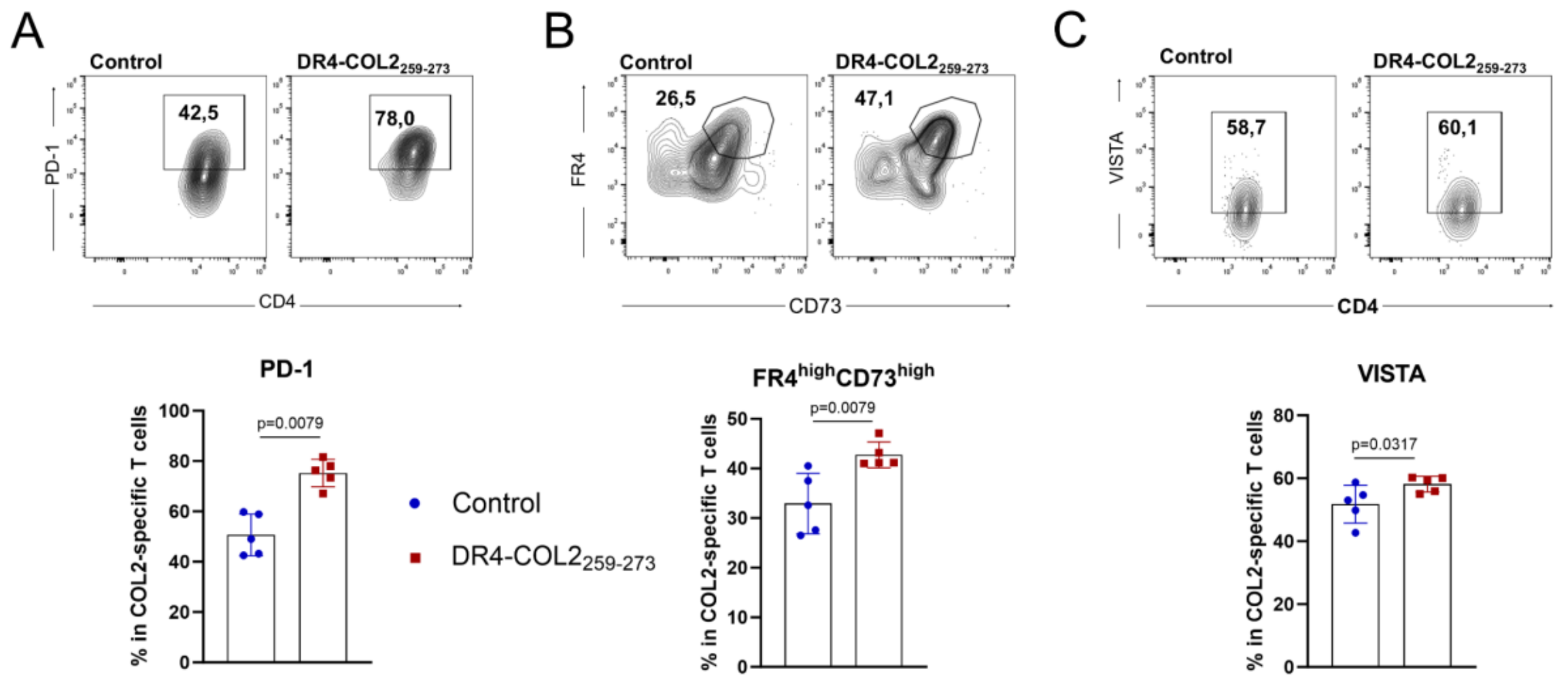

Fig. S6: Tolerogenic phenotype on COL2-specific T cells after DR4-COL2$_{259\text{-}273}$ treatment. Parker mice were immunized with COL2/CFA, at 4 DPI mice were vaccinated with either DR4-CLIP or DR4-COL2$_{259\text{-}273}$ complexes by 3 s.c injections. 15 DPI splenocytes and dLN cells were recovered, stained with the PE-labeled COL2-DR4 tetramer and enriched using anti-PE magnetic beads for immunophenotyping. (**A** to **C**) Vaccination with the DR4-COL2$_{259\text{-}273}$ complexes increases the frequency of PD-1, CD73, FR4, and VISTA among COL2-specific (DR4-COL2$_{259\text{-}273}$+/CD44+) CD4+ T cells. Representative flow cytometry plots depicting the gating strategy for PD-1, CD73, FR4 and VISTA in DR4-COL2$_{259\text{-}273}$+/CD44+/CD4+ T cells. Results are expressed as mean ± SEM. Each dot represents an individual mouse. Statistics were determined with a two-tailed Mann-Whitney test. (Control: DR4-CLIP; DPI: days post-immunization; s.c.: subcutaneously; dLN: draining lymph nodes).

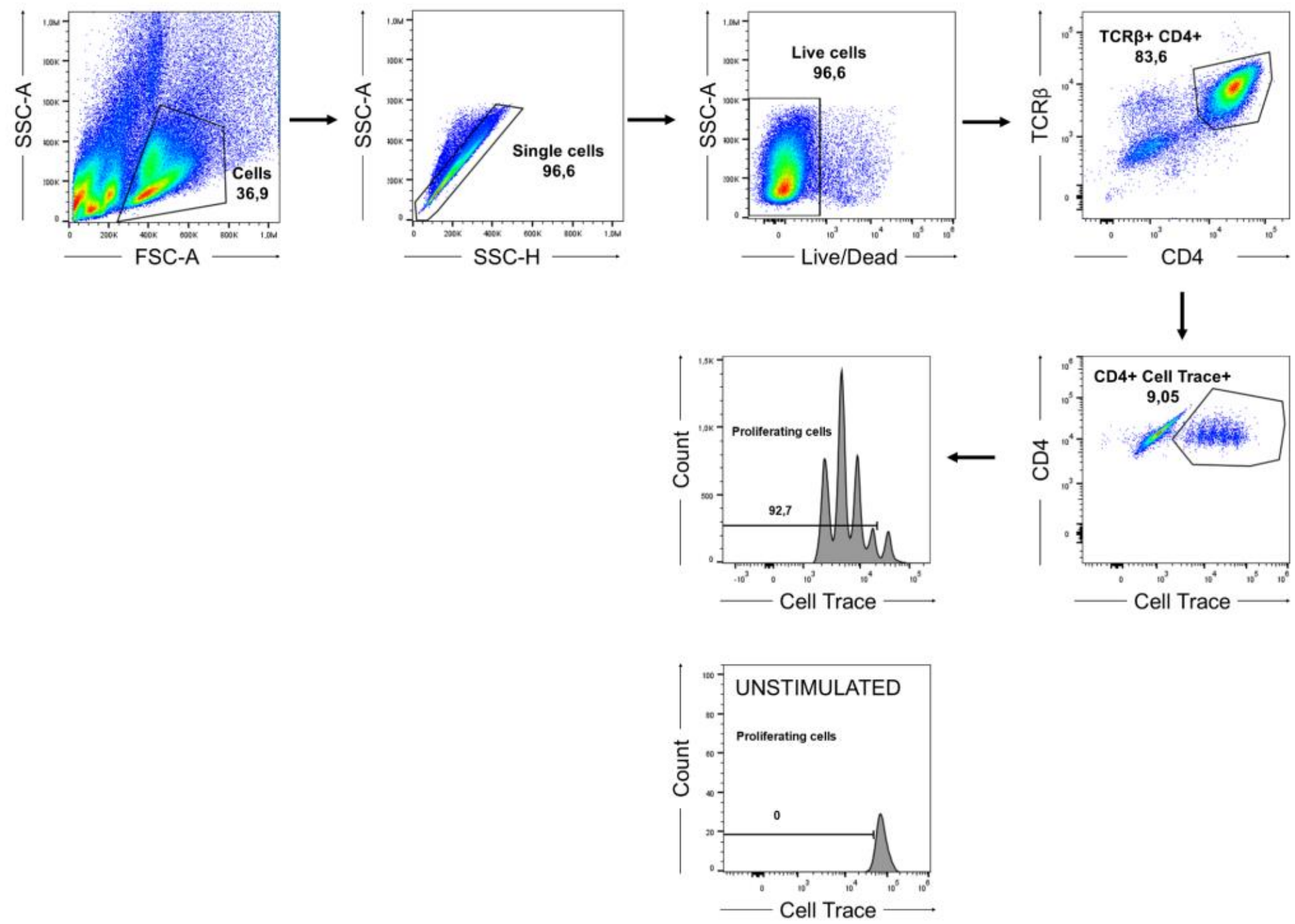

Fig. S7. Gating strategy for analysing proliferation of cell trace-labelled CD4+ T cells from naïve Parker mice in co-culture with CD4 T cells from vaccinated Parker mice corresponding to Figure 5A. Representative gating scheme showing the sequential identification of: lymphocytes, single cells, live cells, TCRβ+ CD4+ T cells, discrimination between cell trace-labelled CD4+ T cells (from naïve Parker mice) and unlabelled CD4+ T cells (from DR4-CLIP or DR4-AL179 vaccinated mice), and proliferating cells within the cell trace-positive population using unstimulated controls as reference.

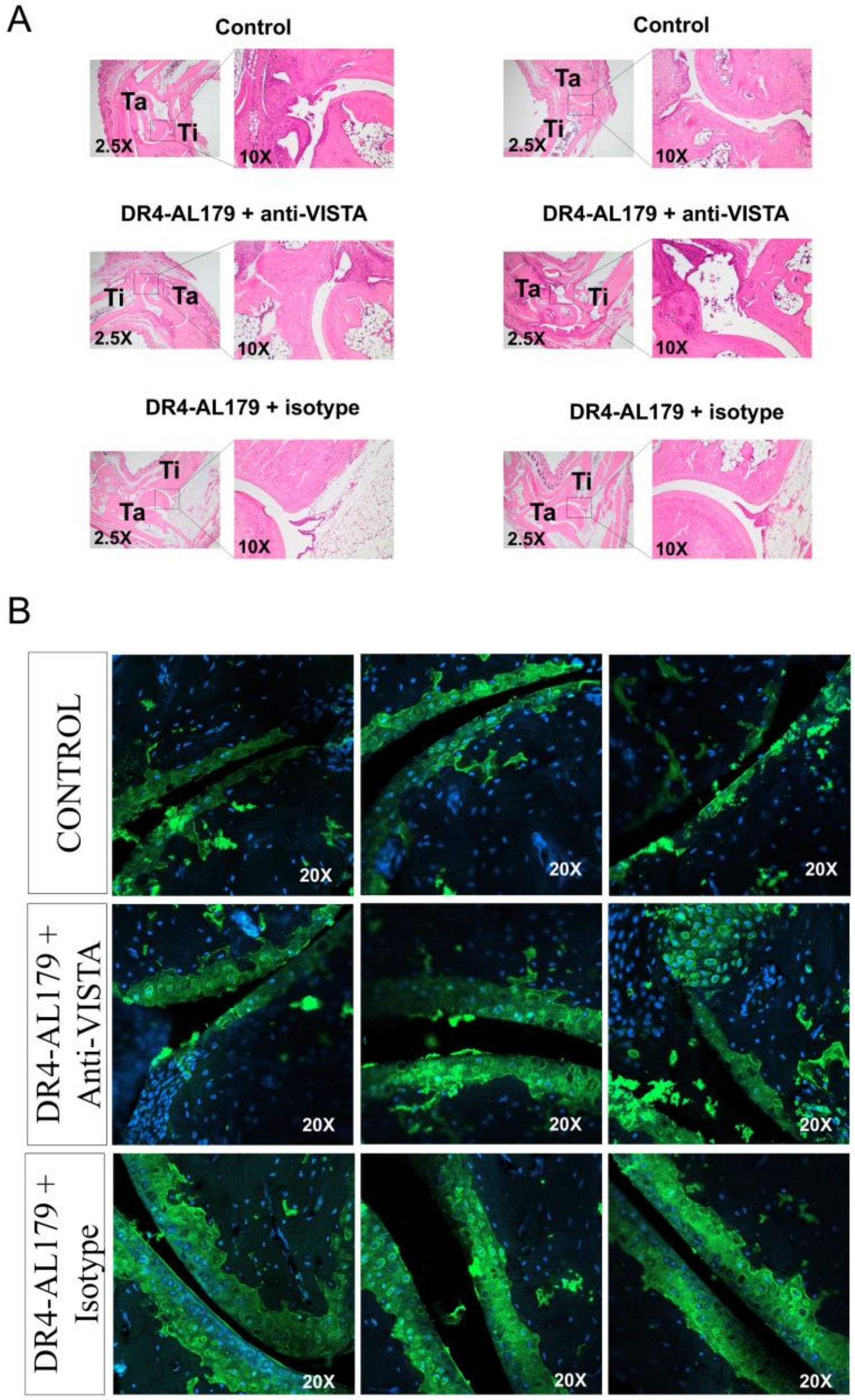

Fig. S8. Histological staining of joint tissues from DR4-CLIP (control), DR4-AL179 + Anti-VISTA and DR4-AL179 + isotype control-treated mice from the adoptive T cell transfer experiment corresponding to Figure 5D. (**A**) Hematoxylin and eosin (H&E) staining of the tibia (Ti) and talus (Ta). Control and DR4-179 + Anti-VISTA groups exhibited increased inflammatory cell infiltration and cartilage/bone erosion compared to DR4-AL179 + isotype control treated mice. (**B**) Immunofluorescence staining COL2 in articular cartilage. DR4-AL179 + isotype control treated mice demonstrated enhanced preservation of COL2 fibrillar structure compared to the rest of the groups.

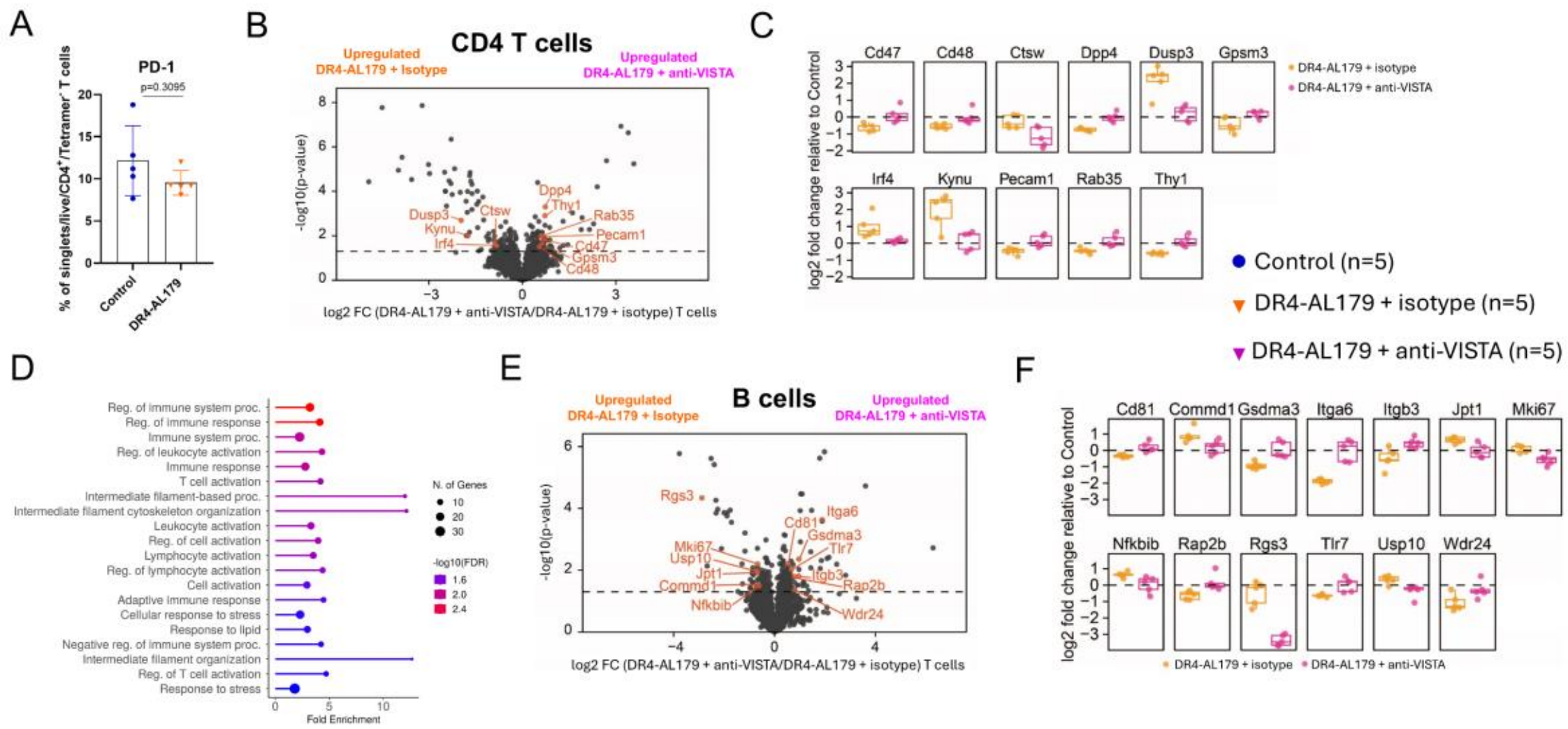

Fig. S9: T and B cell proteomic landscape. (**A**) PD-1 expression by flow cytometry after vaccination with DR4-CLIP or DR4-179 complexes. Gated in live CD4+ DR4-COL2$_{259\text{-}273}$ tetramer- cells. (**B**) Volcano plot comparing differently expressed proteins on FACS sorted CD4+ T cells between DR4-AL179 + isotype (left, orange, n = 5) and DR4-179 + anti-VISTA (right, pink, n=5) adoptively transferred mice. (**C**) Boxplots showing selected differentially expressed proteins comparing sorted CD4+ T cells from DR4-AL179 + isotype and DR4-179 + anti-VISTA (n=5 each) adoptively transferred mice. The horizontal line in the boxplots represents the median, 25th, and 75th percentiles and the whiskers represent measurements to the 5th and 95th percentiles. (**D**) Barplot illustrates the gene enrichment analysis. Each bar represents an enriched GO term. The length of the bar indicates the fold enrichment, while the associated p-value and the number of enriched genes is displayed for each term terms with an FDR < 0.05 were considered significantly enriched. (**E**) Volcano plot comparing differently expressed proteins on FACS sorted B cells between DR4-AL179 + isotype (left, orange, n = 5) and DR4-179 + anti-VISTA (right, pink, n=5) adoptively transferred mice. (**F**) Boxplots showing selected differentially expressed proteins comparing sorted B cells from DR4-AL179 + isotype and DR4-179 + anti-VISTA (n=5 each) adoptively transferred mice. The horizontal line in the boxplots represents the median, 25th, and 75th percentiles and the whiskers represent measurements to the 5th and 95th percentiles. FDR-False discovery rate. (Control: DR4-CLIP).

A

DRA_0401_BirA

MKLCILLAVVAFVGLSLGIKEEHVIIQAEFYLNPDQSGEFMFDFDGDEIFHVDMAKKETVWRLEEFGRFASFEAQGALANIAVDKANLEIMTK
RSNYTPITNVPPEVTVLTNSPVELREPNVLICFIDKFTPPVVNVTWLRNGKPVTTGVSETVFLPREDHLFRKFHYLPFLPSTEDVYDCRVEH
WGLDEPLLKHWEFDASGGGENLYFQGGGGSLTDTLQAETDQLEDEKSALQTEIANLLKEKEKLEFILAAHGGGGSGLNDIFEAQKIEWHE

DRB1_0401_hCLIPmut

MKLCILLAVVAFVGLSLGSAWSHPQFEK**PVSKARMATGALAQA**SGGGS**LVPRGS**GGGGSGDTRPRFLEQVKHECHFFNGTERVRFLDRY
FYHQEEYVRFDSDVGEYRAVTELGRPDAEYWNSQKDLLEQKRAAVDTYCRHNYGVGESFTVQRRVYPEVTVYPAKTQPLQHHNLLVCSV
NGFYPGSIEVRWFRNGQEEKTGVVSTGLIQNGDWTFQTLVMLETVPRSGEVYTCQVEHPSLTSPLTVEWRARSGGGENLYFQGGGGSRI
ARLEEKVKTLKAQNSELASTANMLREQVAQLKQKVMNHHHHHHH

B

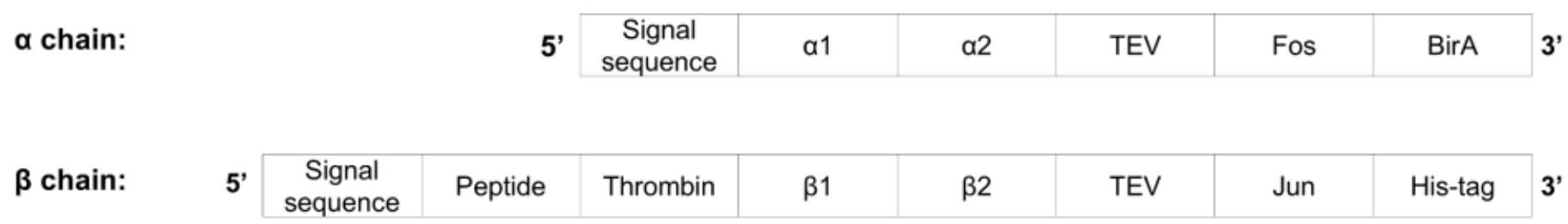

Fig. S10: Design of the DR4-peptide constructs. (**A**) Amino acid sequences of the α chain and the β chain, respectively. The text highlighted in yellow is the signal peptide; the red text indicates the covalently linked mutated human CLIP peptide; the LVPRGS text highlighted in green is a thrombin cleavage site for cleaving off the covalently linked peptide; the text highlighted in magenta indicates the dimerization motif Fos and Jun. (**B**) Features of the construct: signal peptide; C-terminal His-tag for purification; BirA tag for *in vitro* biotinylation; leucine zipper dimerization motif to form alpha and beta chains dimerization; tobacco etch virus (TEV) protease cleavage site for crystallization purposes; thrombin cleavage site (TCS) for exchange to desired peptide.

MATERIAL AND METHODS.

Molecular docking and analyzing protein-protein interactions

The crystal structures of DRB1*04:01 in complex with mutated $COL2_{259\text{-}273}$ (Sequence: AGFAGEQGPAGEP; PDB ID: 7NZF) and T-cell receptor (PDB ID: 4UDT) were retrieved from Protein Data Bank (https://www.rcsb.org/)[1,2] and the PyMOL mutagenesis tool was used to mutate the amino acid residues and create DRB1*04:01-$COL2_{259\text{-}273}$ and DRB1*04:01-AL179, ensuring structural and functional relevance. The mutated files were exported in PDB file format, maintaining conformational fidelity.

The HDOCK server[3] was used to perform protein-protein docking between DRB1*04:01-$COL2_{259\text{-}273}$ and T-cell receptor, as well as DRB1*04:01-AL179 and T cell receptor. The HDOCK server uses a computational algorithm employing a robust scoring function to predict complex formation with high accuracy. We conducted an in-depth analysis of docking scores, utilizing visualization criteria to select the most suitable model thus optimizing the predictive reliability of the docking outcomes. The PyMOL precise measurement tool was used to quantify the spatial distance between key residues involved in protein-protein interactions, ensuring meticulous characterization of binding interfaces.

Immunofluorescence staining of COL2 in joint tissue.

Paraffin-embedded joint sections were baked at 60 °C for 30 min, dewaxed using xylene, and rehydrated using gradient ethanol and finally distilled water. Masked antigens were then retrieved using 1 g pepsin (P7000, Sigma-Aldrich) dissolved in 250 ml 0.01 N HCl at 37 °C for 10 min. After antigen retrieval, slides were incubated with the primary antibody M2139

(IgG2b, Vacara AB, 10 μg/mL) at 4 °C overnight. Finally, AF488-conjugated goat anti-mouse IgG2b antibody (A-21141, ThermoFisher Scientific, 1:100, 37 °C, 1 h) was used to stain the bound IgG2b. Nuclear staining was performed using Hoechst 33342 (Invitrogen) for 10 min at room temperature. Images were acquired using an LSM800 confocal microscope (Zeiss).

Gene Enrichment Analysis

To identify biological pathways and processes associated with the differentially expressed genes, we performed gene enrichment analysis using the ShinyGO[4]. The 68 upregulated and 38 downregulated genes from the differential proteomics analysis were first converted to Ensembl gene IDs using the GRCm38 mouse genome build.

Gene Ontology (GO) Biological Process terms were used as the gene set database for the enrichment analysis. To assess whether the *Vsir* gene partners with other differentially expressed genes in certain pathways, we included *Vsir* in the gene list for the enrichment analysis.

Enrichment p-values were calculated using the hypergeometric test. To correct for multiple hypothesis testing, the Benjamini-Hochberg method was used to calculate false discovery rates (FDR). The fold enrichment for each GO term was defined as the ratio of the percentage of genes associated with that term in the query gene list compared to the percentage among all background genes.

Results of molecular docking

The protein model was obtained from PDB and mutated to create both DR4-COL2$_{259\text{-}273}$ and DR4-AL179. A total of two amino acids (GI) were added at the start of mutated COL2$_{259\text{-}273}$. Additionally, Ala61, Ala64, and Ala70 were replaced with lysine (K) to make DR4-COL2$_{259\text{-}273}$ (GIAGFKGEQGPKGEP). Later this protein model was modified at G265 to create DR4-AL179.

The docking of DR4-COL2$_{259\text{-}273}$ and TCR came out with docked models showing various H-bonds and one salt bridge. The H-bond between COL2$_{259\text{-}273}$ and TCR was Ala261-αSer52, Phe263-αThr54, and Gly265- αThr54. Moreover, the H-bond between MHCII- COL2$_{259\text{-}273}$ and TCR was βGln70-αSer61, αGlu55-αTyr49, αGln57-βGlu101. While the salt bridge was formed between αGln57-βTyr103 (Fig. S1a).

The H-bonds and the salt bridge between DR4-AL179 and TCR were also determined. The H-bond between AL179 and TCR was Ile2-βLeu97. The bonding between MHCII-AL179 and TCR were βAla73-βPhe75, βThr77-βAsn31, αArg50-αThr54, αArg50-αLys55, βGlu87-βThr3, βTyr83-αTyr49, βTyr96-αSer78, βSer179-αGln79, βThr181-αGlu16, βThr181-αLys170, βLys184-αMet167. While the salt bridge formed is β176Glu-αArg165 (Fig. S1b).

The higher number of hydrogen bonds formation between DR4-AL179 with TCR (Fig. S1c) reflects the higher binding affinity and stabilization of this complex formation compared to DR4-COL2$_{259\text{-}273}$.

REFERENCES

1. Rodstrom, K.E., Regenthal, P., and Lindkvist-Petersson, K. (2015). Structure of Staphylococcal Enterotoxin E in Complex with TCR Defines the Role of TCR Loop

Positioning in Superantigen Recognition. PLoS One *10*, e0131988. 10.1371/journal.pone.0131988.

2. Ge, C., Weisse, S., Xu, B., Dobritzsch, D., Viljanen, J., Kihlberg, J., Do, N.N., Schneider, N., Lanig, H., Holmdahl, R., and Burkhardt, H. (2022). Key interactions in the trimolecular complex consisting of the rheumatoid arthritis-associated DRB1*04:01 molecule, the major glycosylated collagen II peptide and the T-cell receptor. Ann Rheum Dis *81*, 480-489. 10.1136/annrheumdis-2021-220500.

3. Yan, Y., Tao, H., He, J., and Huang, S.Y. (2020). The HDOCK server for integrated protein-protein docking. Nat Protoc *15*, 1829-1852. 10.1038/s41596-020-0312-x.

4. Ge, S.X., Jung, D., and Yao, R. (2020). ShinyGO: a graphical gene-set enrichment tool for animals and plants. Bioinformatics *36*, 2628-2629. 10.1093/bioinformatics/btz931.
