# Supplementary material for: Tolerogenic antigen-specific vaccine induces VISTA-enriched regulatory T cells and protects against arthritis in DRB1∗04:01 mice
Source: Mol Ther. 2025 Apr 24;33(8):3528–45. doi: 10.1016/j.ymthe.2025.04.034 (PMC12461628; doi:10.1016/j.ymthe.2025.04.034)
Supplement: Supplementary file 2 [file mmc2.pdf]

Molecular Therapy
Original Article

Tolerogenic antigen-specific vaccine induces VISTA-enriched regulatory T cells and protects against arthritis in DRB1*04:01 mice

Laura Romero-Castillo,[1] Rajan Kumar Pandey,[1] Bingze Xu,[1] Christian M. Beusch,[3] Ana Oliveira-Coelho,[1] Kejsi Zeqiraj,[1] Carolin Svensson,[1] Zhongwei Xu,[1] Huqiao Luo,[1,9] Outi Sareila,[1,2] Pierre Sabatier,[3] Changrong Ge,[1] Lei Cheng,[1] Vilma Urbonaviciute,[1] Alexander Krämer,[1] Cecilia Lindgren,[5] Sabrina Haag,[1] Johan Viljanen,[4] Roman A. Zubarev,[3,6] Jan Kihlberg,[4] Anna Linusson,[5] Harald Burkhardt,[7,8] and Rikard Holmdahl[1,2]

[1]Medical Inflammation Research, Division of Immunology, Department of Medical Biochemistry and Biophysics, Karolinska Institute, 17176 Stockholm, Sweden; [2]Medical Inflammation Research, MediCity Research Laboratory, University of Turku, 20520 Turku, Finland; [3]Division of Physiological Chemistry I, Department of Medical Biochemistry and Biophysics, Karolinska Institute, 17176 Stockholm, Sweden; [4]Department of Chemistry-BMC, Uppsala University, 75237 Uppsala, Sweden; [5]Department of Chemistry, Umeå University, 90187 Umeå, Sweden; [6]Department of Pharmacological & Technological Chemistry, I. M. Sechenov First Moscow State Medical University, 119146 Moscow, Russia; [7]Fraunhofer Institute for Translational Medicine and Pharmacology ITMP, & Fraunhofer Cluster of Excellence for Immune-Mediated Diseases CIMD, Theodor-Stern-Kai 7, 60596 Frankfurt am Main, Germany; [8]Division of Rheumatology, University Hospital Frankfurt, Goethe University, 60596 Frankfurt am Main, Germany; [9]School of Medicine, Shanghai University, Shanghai 200444, China

Rheumatoid arthritis (RA) is a chronic autoimmune disease characterized by joint inflammation, cartilage damage, and bone erosion. Despite improvements with the introduction of biological disease-modifying anti-rheumatic drugs (DMARDs), RA remains an incurable life-long disease. Advancements in peptide-based vaccination may open new avenues for treating autoimmune diseases, including RA, by inducing immune tolerance while maintaining normal immune function. We have already demonstrated the efficacy of a potent vaccine against RA, consisting of the mouse major histocompatibility complex class II (A^q) protein bound to the immunodominant type II collagen peptide $COL2_{259-273}$, which needed to be galactosylated at position 264. To translate the vaccine to humans and to further enhance vaccine efficacy, we modified the glycine residue at position 265 and conjugated it with the human DRB1*04:01 molecule. Remarkably, this modified vaccine (named DR4-AL179) provided robust effectiveness in suppressing arthritis in DRB1*04:01-expressing mice without the need for galactosylation at position 264. DR4-AL179 vaccination induces tolerance involving multiple immunoregulatory pathways, including the activation of V-type immunoglobulin domain-containing suppressor of T cell activation (VISTA)-positive nonconventional regulatory T cells, which contribute to a potent suppressive response preventing arthritis development in mice. This modified RA vaccine offers a novel therapeutic potential for human autoimmune diseases.

INTRODUCTION

Autoimmune diseases pose a growing health burden, affecting approximately 8% of the population worldwide.[1–3] Despite their increasing prevalence, effective preventive and curative treatments for autoimmune conditions remain poorly explored. Among these autoimmune diseases, rheumatoid arthritis (RA) stands out as a common, severe, chronic inflammatory autoimmune disorder characterized by synovial joint inflammation, articular cartilage, and subchondral bone destruction.[4,5] RA affects between 0.5% and 1% of the worldwide population, where women are affected 2–3 times more frequently than men.[6,7] Unfortunately, current therapeutic interventions for RA cannot prevent or cure the disease while potentially increasing patients' susceptibility to infection complications and other comorbidities.[8]

RA is genetically associated with the major histocompatibility complex class II (MHCII) region, particularly including the HLA-DRB1*04:01 haplotype, indicating a key role for autoreactive T cells driving an immune response.[9,10] Despite extensive research, RA's primary autoantigen responsible for T cell activation remains unidentified. However, type II collagen (COL2), a major component of hyaline cartilage, has emerged as one potential autoantigen. This notion is supported by the frequent observation of both potentially pathogenic and protective B cells as well as anti-COL2 antibodies in RA patients,[11,12] along with the susceptibility of experimental

Received 16 December 2024; accepted 22 April 2025;
https://doi.org/10.1016/j.ymthe.2025.04.034.

Correspondence: Laura Romero-Castillo, Medical Inflammation Research, Division of Immunology, Department of Medical Biochemistry and Biophysics, Karolinska Institute, 17176 Stockholm, Sweden.
E-mail: laura.romero.castillo@ki.se
Correspondence: Rikard Holmdahl, Medical Inflammation Research, Division of Immunology, Department of Medical Biochemistry and Biophysics, Karolinska Institute, 17176 Stockholm, Sweden.
E-mail: rikard.holmdahl@ki.se

 © 2025 The Author(s).
Published by Elsevier Inc. on behalf of The American Society of Gene and Cell Therapy.
This is an open access article under the CC BY license (http://creativecommons.org/licenses/by/4.0/).

animals to collagen-induced arthritis (CIA).[13–15] Notably, a COL2 peptide is presented to T cells in mice expressing the MHCII A^q molecule, similar to how it is presented in individuals expressing RA-associated HLA-DRB1*04:01 molecules.[14,16,17] These findings underscore the potential role of COL2-specific immune responses in the pathogenesis of RA.

Considering these findings, we propose to induce immune tolerance by using a tolerogenic vaccine to rebalance the immune homeostasis and potentially cure RA. Tolerogenic vaccination seeks to establish peripheral tolerance against self-antigens while preserving protective immune responses.[18] The discovery of autoreactive T cells specific for the $COL2_{259-273}$ peptide, with or without glycosylation in RA patients carrying the HLA-DRB1*04:01 allele,[19,20] allows an opportunity to modify these cells in a regulatory direction. By inducing a regulatory T cell-dependent response, such a vaccine could alleviate self-reactive immune cells and mitigate autoimmune pathology.

Several strategies have been explored to develop tolerogenic interventions, including whole-antigen-based tolerance,[21,22] antigen-loaded particles,[23,24] and soluble MHCII-peptide complexes.[25,26] Additionally, the adoptive transfer of conventional regulatory T cells (Foxp3+ Tregs)[27] or tolerogenic dendritic cells[28,29] has shown promise in inducing immune tolerance and alleviating autoimmune disorders in preclinical models. However, controlling the immune response remains challenging. Some strategies involving antigen-presenting cells (APCs) may inadvertently lead to undesired immune activation. This potential risk arises when certain co-stimulatory signals shift the T cell response toward a pro-inflammatory or aggressive phenotype, rather than fostering regulatory T cell induction, thereby exacerbating rather than ameliorating immune-mediated pathology.

It has become clear that the development of tolerogenic vaccines requires a detailed understanding of both the vaccine's characteristics and the *in vivo* mechanisms driving immune tolerance. These insights are crucial for achieving precise control over the immune response and ensuring consistent and reliable induction of tolerance.

Our group has previously demonstrated that soluble MHCII (A^q) molecules complexed with the galactosylated $COL2_{259-273}$ peptide are effective in treating CIA in mice interacting with the COL2-specific T cell receptor (TCR).[25,26] Moving forward, we aim to translate these findings into viable therapeutic interventions for human RA patients using DRB1*04:01 combined with a COL2 peptide. To validate the vaccine, our laboratory has developed unique knockin humanized mouse models expressing physiological levels of human MHCII molecules, providing a more accurate platform for evaluating potential RA treatments[14] than MHCII transgenics mouse models previously used. In this study, we identified a novel chemical modification at position 265 of the non-galactosylated $COL2_{259-273}$ peptide, bound to recombinant human MHCII (DRB1*04:01), which resulted in an enhanced tolerogenic vaccination effect. Similar to the galactosylated variant, the new vaccine incorporating the modified ligand (DR4-AL179), which lacks galactosylation, exerts its effect through a distinct set of regulatory T cells. These cells express V-type immunoglobulin (Ig) domain-containing suppressor of T cell activation (VISTA),[30] also known as programmed death-1H (PD-1H) and encoded by the *VSIR* gene.

RESULTS

Screening of modified COL2-peptides as potential vaccine candidates for RA

To develop improved vaccine candidates, we conducted *in vitro* testing of a range of modified glycopeptides derived from the parent peptide $COL2_{259-273}$, galactosylated at lysine 264 (gal$COL2_{259-273}$) (Figure 1A). Our design strategy focused on two key residues essential for TCR recognition: phenylalanine 263 and glycine 265. We systematically evaluated each modified peptide against the parent gal-$COL2_{259-273}$, assessing both MHCII-binding capacity (Figure 1B) and T cell activation potential using glycosylated epitope-specific T cell hybridoma clones (Figure 1C). The DR4-binding evaluation revealed that most modified peptides retained binding affinities comparable to the parent gal-$COL2_{259-273}$ peptide (Figure 1B). In T cell activation assays, AL170 exhibited an enhancement in T cell response across all hybridoma clones tested. AL177 and AL178 selectively increased activation of the mDR17.2 clone, while AL179 significantly stimulated the mDR1.1 clone (Figure 1C). Through this comprehensive screening, four peptides emerged as lead candidates (AL170, AL177, AL178, and AL179) distinguished by their enhanced MHCII binding and alterations in T cell contact points.

Due to the presence in humans of autoreactive T cells specific for non-glycosylated COL2,[31] we opted for the development of a non-galactosylated vaccine. Additionally, the complexity of synthesizing glycosylated vaccines presents significant challenges for scalability and further development. The selected peptides are depicted in Figure 2A.

To screen their functional effect, we used the delayed-type hypersensitivity (DTH) model mediated by interferon (IFN)-γ-producing type 1 T helper (Th1) cells, a test predicting an arthritis protective effect.[26] Mice were vaccinated using osmotic pumps utilizing various complexes comprising human MHCII (DRB1*04:01, named DR4) combined with either the unmodified $COL2_{259-273}$ peptide or modified peptides (AL170, AL177, AL178, and AL179). A DR4 complex containing a mutated CLIP (class II-associated invariant chain peptide) served as the control. The experimental scheme is depicted in Figure 2B. Among the peptides tested, AL179 exhibited the most efficient reduction in ear swelling compared to the control group (Figure 2C).

AL179 bound well to MHCII and demonstrated enhanced activation of COL2-specific T cell hybridoma cells (3H8) in comparison with DR4-$COL2_{259-273}$ (Figures 2D and 2E). In parallel, docking analysis of DR4-$COL2_{259-273}$ and DR4-AL179 with the TCR revealed an increased number of hydrogen bonds reflecting a stronger binding affinity and stabilization of the complex formation between

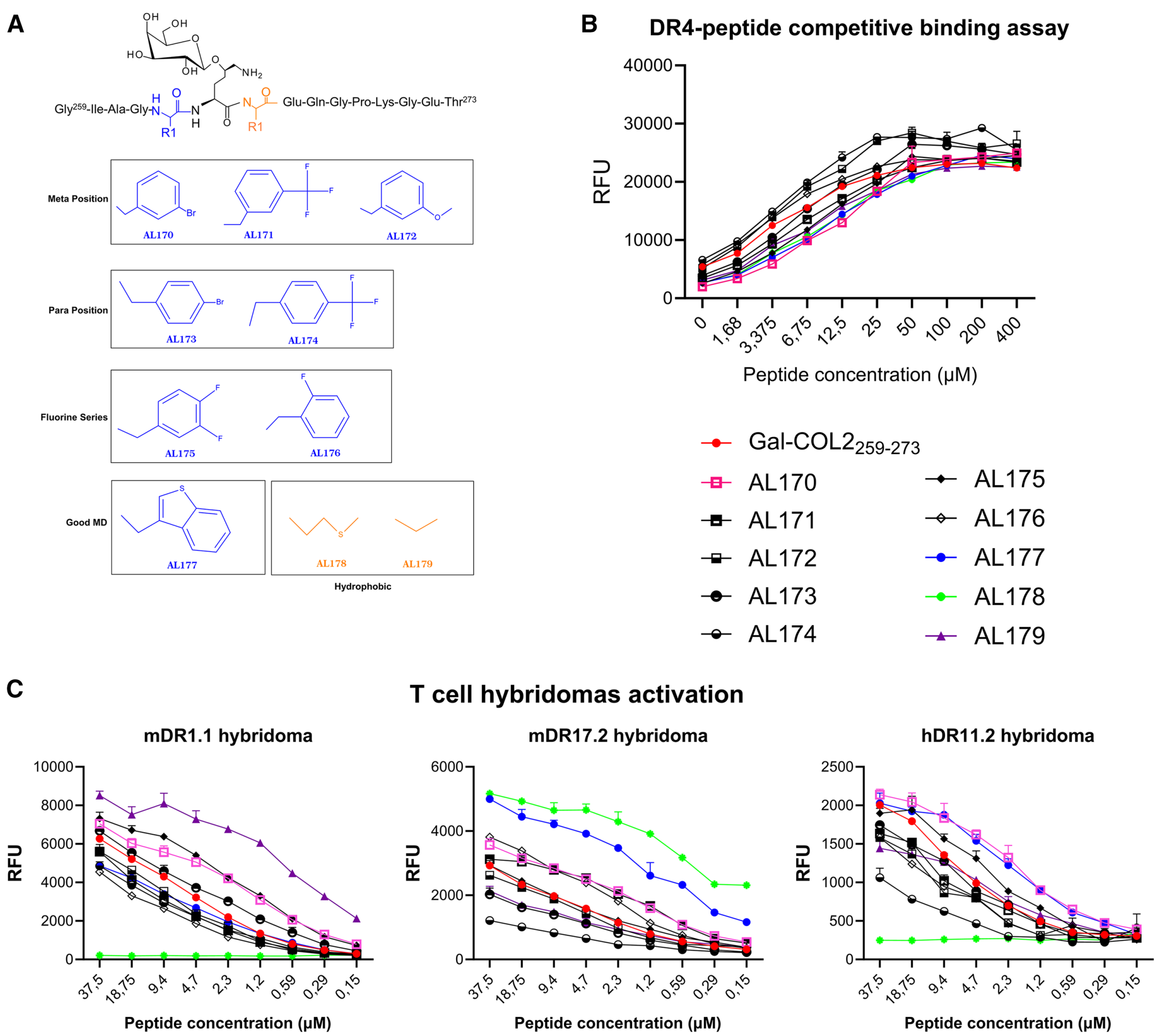

Figure 1. Design and evaluation of modified COL2$_{259-273}$ glycopeptides as vaccine candidates

(A) Schematic representation of synthesized COL2 glycopeptide modifications. The parent peptide gal-COL2$_{259-273}$ (galactosylated at lysine 264, galCOL2$_{259-273}$) was modified at either phenylalanine 263 (blue) or glycine 265 (orange). Phenylalanine modifications included meta-position substituents (AL170, AL171, and AL172), para-position substituents (AL173 and AL174), fluorine derivatives (AL175 and AL176), and molecular-dynamics-optimized modification (AL177). Glycine was modified with hydrophobic substituents (AL178 and AL179). (B) DRB1*04:01 (DR4) competitive-binding assay comparing modified peptides to parent gal-COL2$_{259-273}$. (C) T cell-activation assay using three DR4-gal-COL2-restricted T cell hybridoma clones (mDR1.1, mDR17.2, and hDR11.2). T cell activation was assessed by measuring IL-2 secretion into the medium by ELISA. RFU, relative fluorescence units.

DR4-AL179 and TCR compared to DR4-COL2$_{259-273}$ (Figure S1). Furthermore, recognizing the need for a more applicable administration route for human translation, we compared osmotic pump delivery with three subcutaneous (s.c.) administrations every 4 h. Our findings revealed no discernible differences in ear swelling or interleukin (IL)-17A secretion (Figures 2F and 2G, respectively), prompting the selection of the latter route for subsequent experiments.

DR4-AL179 vaccine alleviates autoimmune arthritis

Next, we assessed the efficacy of the DR4-AL179 vaccine in ameliorating arthritis in the CIA model. The experimental scheme is depicted in Figure 3A. Treatment with DR4-AL179 significantly reduced both the incidence and the severity of arthritis (Figure 3B). In contrast, the native non-glycosylated DR4-COL2$_{259-273}$ vaccine did not affect arthritis (Figure 3B) despite previously showing a reduction in ear swelling in the DTH model (Figures 2C and 2F). The therapeutic effect of DR4-AL179 in the CIA model was further supported by histological analysis of paw sections, revealing reduced immune cell infiltration and bone erosion (Figures 3C and S2A) along with enhanced preservation of collagen fiber structure (Figure S2B) compared to the control group. Additionally, serum cytokine analysis on day 20 post immunization revealed a marked increase in the anti-inflammatory IL-10 cytokine and a reduction in the pro-inflammatory cytokines IFN-γ and IL-6 (Figure 3D) after vaccination with DR4-AL179 complex. Notably, spleen analysis at the terminal point (day 80) showed a significant

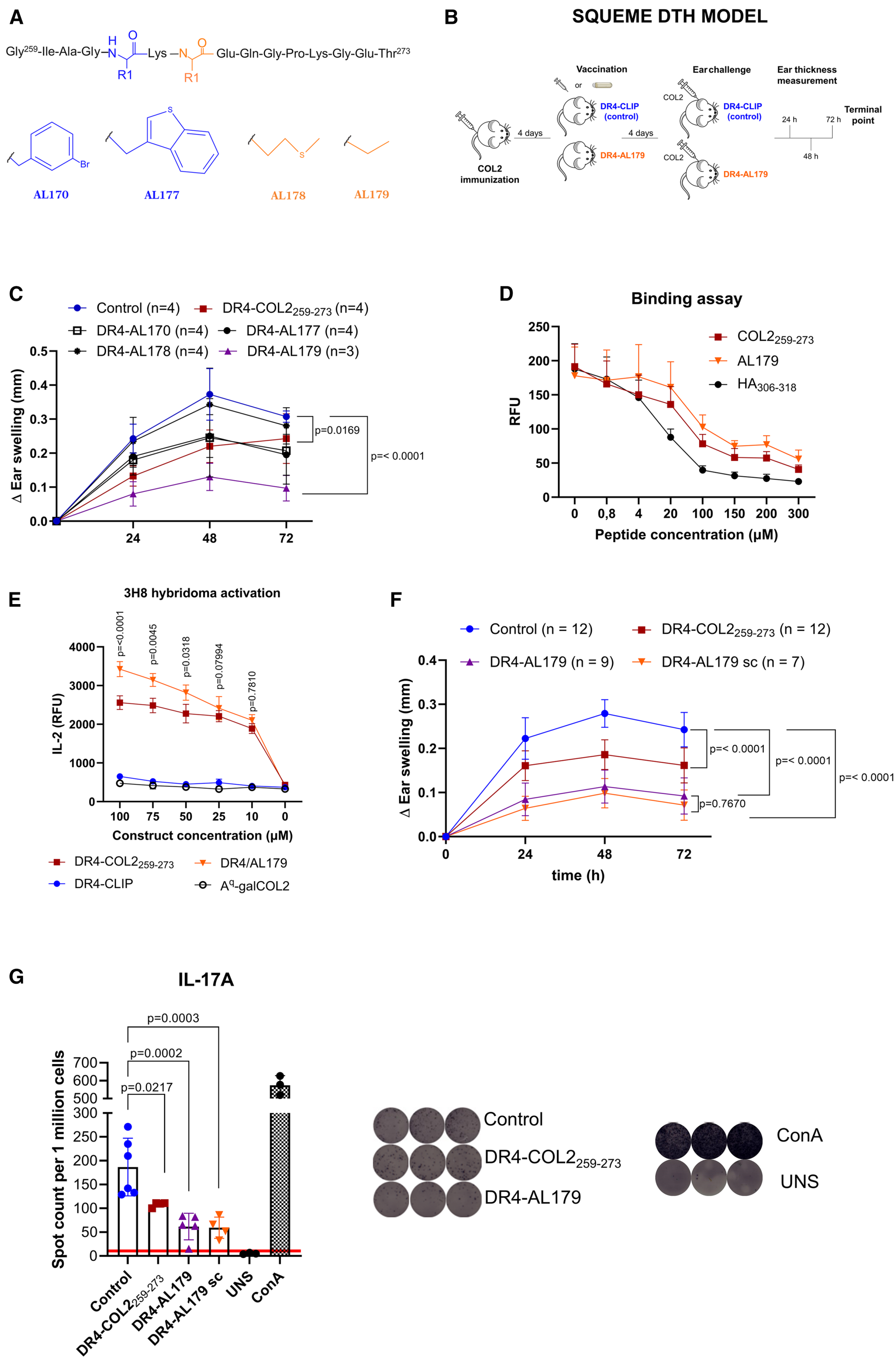

(legend on next page)

reduction in COL2-specific T cells in the DR4-AL179-vaccinated group (Figure 3E). The gating strategy of COL2-specific T cells at CIA terminal point is depicted in Figure S3.

Moreover, vaccination with DR4-AL179 resulted in a notable decrease in anti-COL2 IgG antibody levels in comparison to the control group vaccinated with DR4-CLIP (Figure 3F). This finding was corroborated by a bead-based immunoassay that detected epitope-specific anti-COL2 peptide IgG antibodies, revealing both epitopes spreading over time and a marked reduction in antibody levels in the DR4-AL179 vaccination group, particularly against most COL2 peptides at day 45 post immunization (Figure S4). Remarkably, DR4-AL179 vaccination significantly reduced the arthritogenic U1 antibody[32] levels during both the acute phase of disease at day 45 post immunization (Figure 3G) and at the terminal point (Figure 3H).Finally, we demonstrated that the suppressive effect of T cells from DR4-AL179-vaccinated mice could be transferred, protecting recipient mice from CIA development (Figure 3I).

Vaccination with DR4-AL179 expands antigen-specific CD4+ tolerogenic T cells

We found that the administration of DR4-AL179 to Parker mice led to a reduction in the number of COL2-specific T cells (Figure 4A). The COL2-specific T cells from DR4-AL179-vaccinated mice were investigated for phenotypes earlier selected to be of importance for A^q-COL2 vaccination.[26] Clearly, the T cells from the DR4-AL179-vaccinated mice had an increased expression of cell markers associated with a tolerogenic phenotype as compared with the DR4-CLIP control group. We observed higher PD-1 expression after DR4-AL179 vaccination (Figure 4B). Similarly, CD73, folate receptor 4 (FR4) (Figure 4C) and VISTA (Figure 4D) were upregulated after vaccination with DR4-AL179. It has been reported that $CD73^{high}FR4^{high}$ T cells have the capacity to differentiate into either FOXP3-expressing Tregs or IL-10-producing CD49+LAG-3+ unconventional regulatory T cells (Tr1 cells) depending on the specific physiological context.[33–35] However, in this sense, we observed that vaccination with DR4-AL179 did not increase the FOXP3 conventional regulatory T cells (Figure 4E) but rather increased the non-classical regulatory T cells, the Tr1 population (Figure 4F).

VISTA, a member of the B7 family of immune inhibitory molecules, is known to negatively regulate immune responses, maintain peripheral tolerance, and control autoimmunity.[36] Our previous work in tolerogenic vaccination hypothesized that VISTA plays a crucial role in vaccination efficacy in A^q mice.[26] Literature suggests a higher expression of VISTA on regulatory T cells.[37] Similarly, we observed a higher VISTA expression on FOXP3 Tregs (Figure 4G) and in Tr1 T cells (Figure 4H), although no difference in FOXP3 expression after vaccination with DR4-AL179 was observed (Figure 4E). The gating strategy and a summary table of the flow cytometry analyses shown in Figure 4 are presented in Figure S5.

Although vaccination with DR4-$COL2_{259-273}$ failed to significantly ameliorate autoimmune arthritis in the CIA model (Figure 3B), it induced elevated expression of PD-1 and VISTA on COL2-specific T cells and increased the frequency of $CD73^{high}FR4^{high}$ T cells (Figure S6). Similar changes were observed following DR4-AL179 vaccination. However, the increase in VISTA expression and frequency of $CD73^{high}FR4^{high}$ T cells was less pronounced in DR4-$COL2_{259-273}$-vaccinated mice compared to those receiving DR4-AL179.

The role of VISTA in shaping vaccine efficacy

Building upon our findings in A^q mice, we extended our investigation of VISTA's role in vaccination efficacy to our humanized mouse model by conducting an *ex vivo* T cell suppression assay. Ten days post immunization, CD4+ T cells were enriched from the spleens of vaccinated mice and co-cultured with purified, CellTrace-labeled CD4+ T cells from naive Parker mice, which were activated with anti-CD3 and anti-CD28 antibodies. The CD4+ T cells from vaccinated mice were pretreated with anti-VISTA, anti-PD-1, or isotype control antibodies. Blocking VISTA resulted in a reduced suppressive effect compared to the isotype control, whereas blocking PD-1 had no significant impact (Figure 5A). The gating strategy of the suppression assay is presented in Figure S7.

Following the findings in the suppression assay, we further investigated VISTA's influence on vaccination efficacy by performing an adoptive transfer experiment. CD4+ T cells from vaccinated mice were pretreated with either anti-VISTA or isotype control antibodies and

Figure 2. The synthetic modified-COL2 peptide AL179 stands out as the best vaccine candidate

(A) Schematic design of the synthesized non-galactosylated COL2-peptides. In blue are peptide modifications on the phenylalanine residue at position 263 and, in orange, those on glycine residue at position 265. (B) Experimental procedure of the DTH model. Parker mice were immunized with COL2/CFA i.d. and 4 days later, mice were vaccinated s.c. with DR4-peptide complexes using either osmotic pumps or three injections. 8 DPI, mice were challenged i.d. in the ear with 10 μg of bCOL2 or 10 μg of acetic acid. Ear thickness was measured using a caliper at 24, 48, and 72 h post challenge. Mice were terminated 72 h post challenge. (C) DTH reactions (ear swelling) following treatment with DR4-COL2 complexes using osmotic pumps. Number of mice per group are indicated in parentheses. (D) Binding affinity of AL179 and $COL2_{259-273}$ peptides with the DRB1*04:01 molecule. Influenza hemagglutinin (HA) peptide ($HA_{306-318}$) was used as a positive control. Data are representative of three experiments. (E) Native-COL2-specific T-hybridoma (3H8) activation by DR4-COL2 complexes. T cell activation was assessed by measurement of the IL-2 secreted into the medium by ELISA. DR4-CLIP and A^q-galCOL2 complexes were used as negative controls. Data are representative of three experiments. (F) DTH reactions (ear swelling) following treatment with DR4-COL2 complexes using osmotic pumps or s.c. injections. Numbers of mice per group are indicated in parentheses. (G) Measurement of antigen-specific IL-17A T cell response against the COL2 protein assessed by ELISpot in inguinal lymph nodes 72 h after the DTH model. ConA was used as a positive control (signal exceeds 600 SFC/10^6 cells) while unstimulated cells (UNS) incubated in complete medium alone served as a negative control. The red line indicates the positive threshold determined using the distribution-free resampling (DFR(2×)) test, a standard method for ELISpot analysis. Representative ELISpot wells show antigen-specific IL-17A production by lymph node cells from vaccinated mice. (C–G) Results are expressed as mean ± SEM. Statistics were determined using a two-way ANOVA with Sidak's multiple-comparisons test. Control, DR4-CLIP.

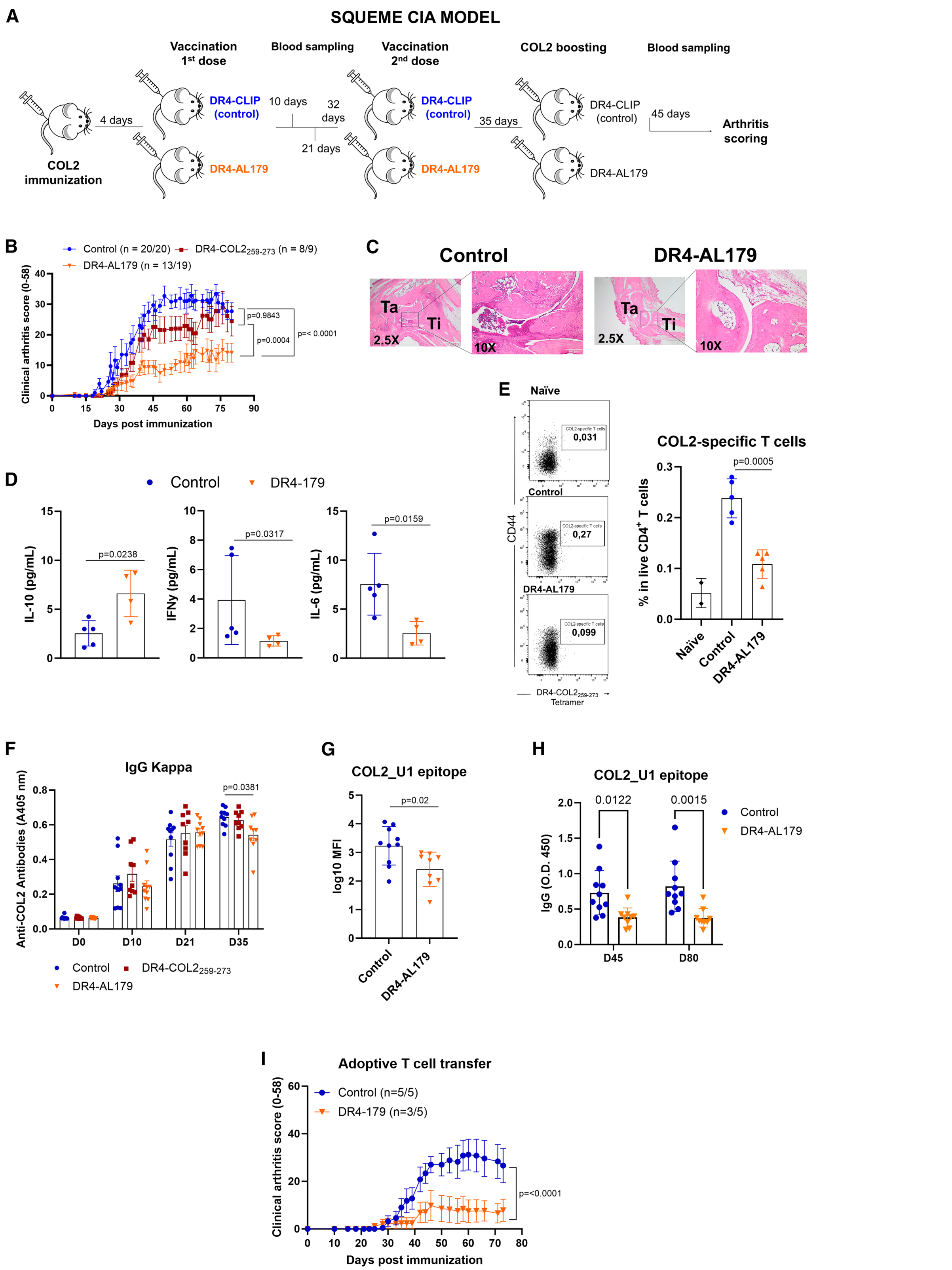

(legend on next page)

transferred into immunized recipient Parker mice. A control group received T cells from unvaccinated mice. The experimental scheme is depicted in Figure 5B. VISTA expression levels in different groups were assessed by flow cytometry before transfer (Figure 5C). Anti-VISTA treatment impaired the therapeutic capacity of T cells to ameliorate autoimmune arthritis, while T cells treated with the isotype control effectively suppressed disease progression (Figure 5D), consistent with findings from the *ex vivo* T cell-suppression assay (Figure 5A). Moreover, in peripheral blood 35 days post immunization (DPI), VISTA expression remained upregulated in the DR4-AL179 + isotype group compared to controls, whereas it was significantly downregulated in the DR4-AL179 + anti-VISTA group (Figure 5E). Histological analysis of joint sections confirmed disease severity, revealing immune cell infiltration and bone erosion (Figures 5E and S8A) in both DR4-CLIP and DR4-AL179 + anti-VISTA groups, accompanied by significant degradation of collagen fiber architecture (Figure S8B) compared to the DR4-AL179 + isotype control group.

Proteomic changes in T and B cells following adoptive T cell transfer from DR4-AL179 or DR4-CLIP-vaccinated mice

In the context of the adoptive transfer experiment, we analyzed the proteomic landscape of both CD4+ T cells and B cells isolated from the dLNs and spleens of recipient mice at the terminal time point. The objective was to identify specific proteomic shifts to characterize the downstream pathways.

An upregulation of PD-1 protein was observed in fluorescence-activated cell sorting (FACS)-sorted CD4+ T cells from DR4-AL179 + isotype-treated mice (Figures 6A and 6B). This finding is consistent with our previous observations in COL2-specific T cells analyzed by flow cytometry (Figure 4B).

To ensure the results in Figures 6A and 6B are attributed to COL2-specific T cells and not driven by nonspecific infection tolerance, we analyzed PD-1 expression in non-COL2 T cells (tetramer-negative fraction) using flow cytometry (Figure S9A).

In addition, in DR4-AL179 + isotype-treated mice, T cells displayed a downregulation of proteins indicating reduced T cell activation[38–40] but an upregulation of proteins involved in regulatory pathways[41–44] (Figures 6A and 6B). We validated the reduced expression of CD48 and THY1, proteins associated with T cell activation, by flow cytometry on COL2-specific T cells in DR4-AL179-vaccinated mice 15 DPI (Figure 6C). Notably, comparing the CD4+ T cell proteomic landscape between DR4-AL179 + isotype and DR4-AL179 + anti-VISTA-treated mice (Figures S9B and S9C) showed that VISTA blockage resulted in a proteomic profile similar to the control group (DR4-CLIP). Gene Ontology (GO) enrichment analysis of differentially expressed proteins from the proteomics data on CD4 T cells revealed that VISTA plays a significant role in most identified pathways (Figure S9D).

To understand whether transferred T cells affect B cell activation, we conducted mass spectrometry-based proteomics on FACS-sorted B cells from DR4-AL179 isotype-treated mice compared to controls. B cells in DR4-AL179 isotype-treated mice showed a downregulation of proteins related to B cell activation,[45–47] migration,[48,49] and antibody response, particularly IgG, IgM, and IgG1 isotypes. Conversely, proteins associated with tissue damage resolution in inflammatory arthritis[50] and negative regulation of nuclear factor (NF)-κB activation[51] were upregulated in DR4-AL179 isotype-treated mice compared to controls (Figures 6D and 6E). We validated the reduced secretion of anti-COL2 IgG and anti-COL2 IgG1 by ELISA from serum at different time points during the transfer experiment (Figures 6F and 6G). Reduced expression of CXCR5 was confirmed by flow cytometry in DR4-AL179-vaccinated mice 15 DPI compared to DR4-CLIP-vaccinated mice (Figure 6H). Additionally, proteins involved in B cell migration, such as ALCAM (CD166)[52] and C-C chemokine receptor type 7 (CCR7),[53] were also downregulated in DR4-AL179-vaccinated mice 15 DPI (Figure 6I). Finally, differences in the B cell proteomic landscape between DR4-AL179 + anti-VISTA and DR4-AL179 + isotype control mice (Figures S9E and S9F) showed upregulation of proteins related to B cell activation and migration, similar to the differences observed between DR4-AL179 + isotype-treated mice and the control group.

DISCUSSION

We have developed a novel tolerogenic vaccine for the prevention of autoimmune arthritis. Unlike previous formulations, this new

Figure 3. DR4-AL179 vaccination reduces arthritis severity and modulates immune responses in the CIA model

(A) Experimental procedure of the CIA model. Parker mice were immunized with COL2/CFA i.d. and at 4 and 32 DPI mice were vaccinated s.c. with DR4-peptide complexes. At 35 DPI, the mice were boosted with 50 μg of bCOL2 in IFA i.d. Blood samples were collected at 10, 21, 35, and 45 DPI and at the terminal point. (B) Arthritis severity was scored throughout the experiment. CIA in vaccinated mice as assessed by mean clinical score of arthritis severity and incidence of arthritis (number of arthritic mice/total number of mice). Data from two pooled experiments. (C) Representative histological examination by H&E staining of the tibia (Ti) and talus (Ta) from control and DR4-AL179-vaccinated mice. Cell infiltration and cartilage/bone destruction are higher in the control group. (D) Serum cytokine levels of IL-10, IFN-γ, and IL-6 after 20 DPI. (E) Vaccination with DR4-AL179 complexes reduces frequencies of COL2-specific T cells (CD44+ DR4-$COL2_{259\text{-}273}$ tetramer+) compared to DR4-CLIP (control) vaccinated mice at day 80 (terminal point) of the CIA model (B). Naive Parker mice (non-COL2 immunized) were used as a negative control. Representative gates are shown. (F) Anti-COL2 antibody levels of total IgG (anti-kappa) from vaccinated mice at different time points along CIA model measured by ELISA. (G) IgG antibodies binding to the triple-helical COL2 epitope U1 in sera collected from vaccinated mice at 45 DPI measured by bead-based flow immunoassay and quantified as log10-transformed mean fluorescence intensity (MFI). (H) IgG antibodies binding to the triple-helical COL2 epitope U1 in sera collected from vaccinated mice at 45 and 80 DPI measured by ELISA. (I) Clinical scoring of arthritis severity following the transfer of T cells from DR4-CLIP-vaccinated and DR4-AL179-vaccinated Parker mice into COL2-immunized recipient Parker mice. The numbers of arthritic mice/total number of mice are represented. Results are expressed as mean ± SEM. Each dot represents an individual mouse. Statistics in (B), (E), (F), and (H) experiments represented were determined using two-way ANOVA with Sidak's multiple-comparisons test, and in (D), (G), and (I) were determined with a two-tailed Mann-Whitney test. Control, DR4-CLIP; OD, optical density.

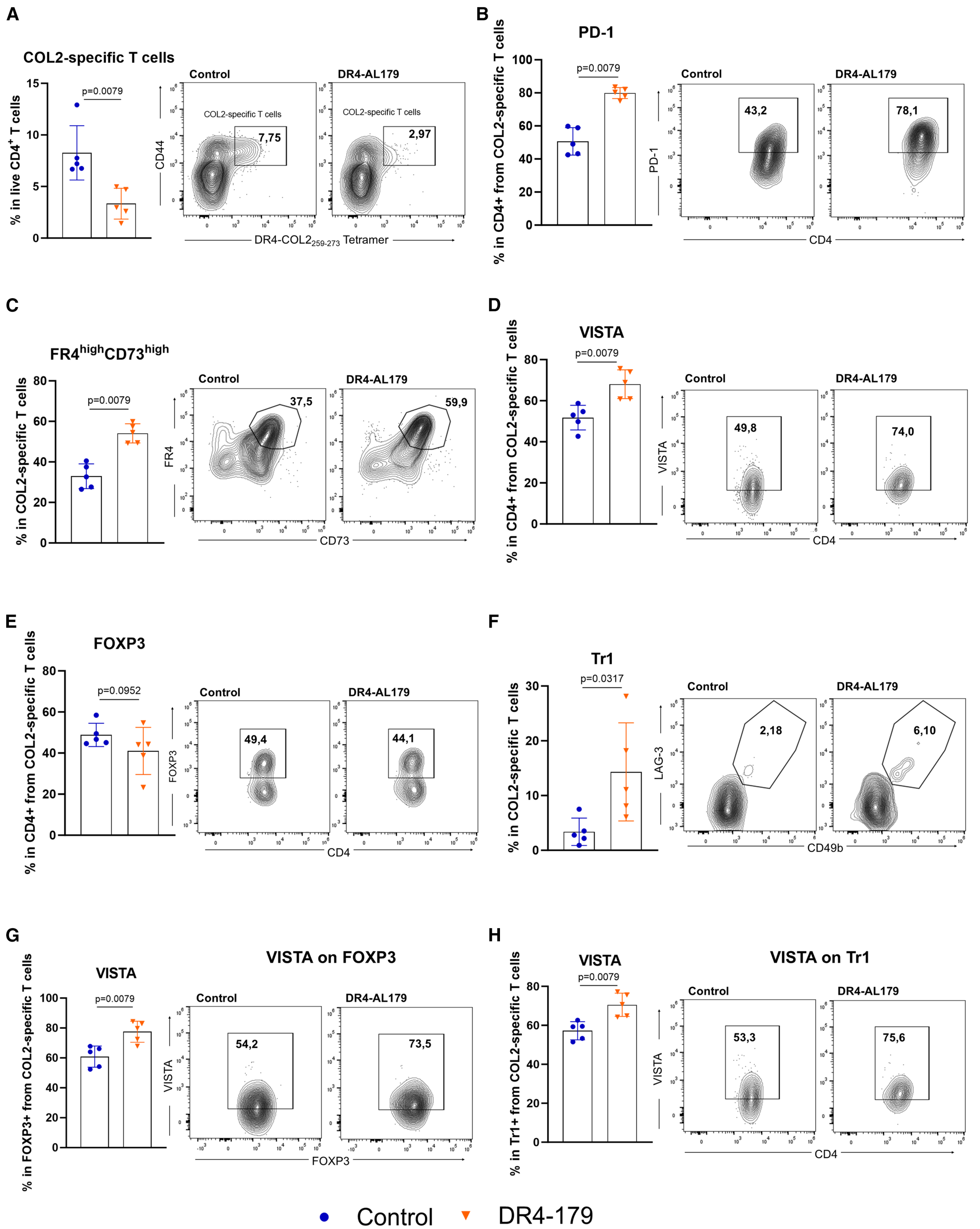

(legend on next page)

vaccine does not require galactosylation, a key structure that was crucial for the efficacy of the described $COL2_{259-273}$ peptide when bound to relevant MHCII molecules.[25,26] The new vaccine is based on a chemical modification of the glycine at position 265 and it induces protection against arthritis through activation of VISTA-positive-non-conventional regulatory T cells, similar to our previously described galactosylated $COL2_{259-273}$ vaccine in A^q mice.

Reactive T cells against the immunodominant $COL2_{259-273}$ peptide are present in the peripheral blood and synovial fluid of RA patients.[19,20,54] Similar to the A^q or DRB1*04:01-expressing mice, most of these T cells recognize the hydroxylation or galactosylation of the lysine residues within the peptide.[55] Interestingly, MHCII-COL2 complexes lacking galactosylation fail to reduce arthritis severity in mice, underscoring the importance of post-translational modifications in therapeutic efficacy.[25] In this study, we present a novel vaccine comprising a complex of a chemically modified $COL2_{259-273}$ peptide (AL179) with the RA-associated MHCII allele DRB1*04:01. By modifying the glycine residue at position 265, a key P3 position for TCR recognition,[56,57] we created a peptide that induced tolerance even without galactosylation. This substitution enhanced the peptide interaction with the TCR without compromising MHCII binding. These findings suggest that the tolerogenic capacity of naturally occurring regulatory peptides can be enhanced through targeted synthetic modifications.

To facilitate the translation of this vaccine to human clinical use, we evaluated its efficacy using our novel MHCII humanized mouse model. The model was specifically developed to overcome the artifacts observed in traditional MHCII transgenic mice, which arise due to defective or deficient expression of MHCII molecules. The vaccine was tested in DRB1*04:01-expressing mice, which have been shown to physiologically express the human MHCII molecule and exhibited allele-restricted immune responses and arthritis association, consistent with human RA.[14]

One of the critical challenges in MHCII-peptide-based therapies is the rapid clearance of these complexes from the body. In previous studies, this issue was mitigated using osmotic pumps implanted s.c. for continuous delivery.[26] In this study, we demonstrate that three s.c. administrations alone are equally effective, offering a more practical and scalable alternative to prepare the therapy for human application.

The AL179 peptide, when complexed with the RA-associated MHCII allele DRB1*04:01, significantly alleviated autoimmune arthritis and induced a robust tolerogenic response, reducing COL2-specific T cells and activating regulatory T cell populations. The therapeutic efficacy of our DR4-AL179 vaccine likely involves multiple complementary tolerogenic mechanisms. While our data strongly support the role of VISTA-expressing regulatory T cells, the observed upregulation of PD-1 and increase in $CD73^{high}FR4^{high}$ populations among COL2-specific T cells suggest potential contributions from T cell exhaustion and anergy pathways. These mechanisms may work in concert to establish robust immune tolerance, with VISTA enrichment serving as a key component of this multi-faceted response.

In our previous work, we hypothesized that VISTA plays a crucial role in vaccination efficacy in A^q mice by performing some *ex vivo* experiments.[26] Building on this finding, we confirmed its significant contribution through an adoptive T cell-transfer experiment. Our study demonstrated that blocking VISTA substantially impaired the capacity of transferred T cells to ameliorate autoimmune arthritis, highlighting it as a crucial pathway in the vaccine's mechanism of action. VISTA possesses distinct characteristics as a potent negative regulator of T cell function.[58,59] Its role as an immune checkpoint is highlighted by observations that *Vsir*-knockout mice exhibit exacerbated T cell-mediated immune pathology across various disease models, including graft-versus-host disease (GVHD), experimental autoimmune encephalomyelitis (EAE), and RA,[36,60] and developed spontaneous autoimmunity resembling systemic lupus erythematosus (SLE).[61] Consequently, VISTA deficiency led to the breakdown of self-tolerance and the emergence of inflammatory T cell responses.[30] Therefore, engaging VISTA post vaccination enhances an immunosuppressive phenotype crucial for augmenting T cell tolerance. This VISTA-mediated immunosuppressive phenotype may work synergistically with other tolerance mechanisms, including the induction of T cell anergy and deletion of autoreactive cells, to establish robust and lasting protection against autoimmune responses. Interestingly, the identification and characterization of this phenotype could allow us to follow and monitor the vaccination effect in humans. In conclusion, our study

Figure 4. DR4-AL179 vaccination enhances a tolerogenic phenotype in COL2-specific T cells

(A) Vaccination with DR4-AL179 complexes reduces frequencies of COL2-specific T (CD44+ DR4-$COL2_{259-273}$ tetramer+) cells after 15 DPI. Parker mice were immunized with COL2/CFA, and at 4 DPI mice were vaccinated with DR4–peptide complexes by three s.c. injections. 15 DPI splenocytes and dLN cells were recovered, stained with the PE-labeled DR4-$COL2_{259-273}$ tetramer, and enriched using anti-PE magnetic beads for immunophenotyping by flow cytometry. Representative flow cytometry plots depicting the gating strategy for DR4-$COL2_{259-273}$+/CD44+ (COL2-specific) cells in live CD4+ T cells. (B–D) Vaccination with the DR4-AL179 complexes increases the frequency of PD-1, CD73, FR4, and VISTA in COL2-specific T cells. Representative flow cytometry plots depicting the gating strategy for PD-1, CD73, FR4, and VISTA in COL2-specific T cells (CD44+ DR4-$COL2_{259-273}$ tetramer+). (E) Frequency of FOXP3+ in CD4+ from COL2-specific T cells. Representative flow cytometry plots depicting the gating strategy for FOXP3 in CD4+/CD44+/DR4-$COL2_{259-273}$T cells. (F) Frequency of Tr1 (LAG-3+/CD49+) in COL2-specific T cells. Representative flow cytometry plots depicting the gating strategy for LAG-3+/CD49+ (Tr1) in CD4+/CD44+/DR4-$COL2_{259-273}$T cells. (G) Frequency of VISTA+ in FOXP3+ from COL2-specific T cells. Representative flow cytometry plots depicting the gating strategy for VISTA+ in FOXP3+/DR4-$COL2_{259-273}$+/CD44+/CD4+ T cells. (H) Frequency of VISTA+ in Tr1 from COL2-specific T cells. Representative flow cytometry plots depicting the gating strategy for VISTA+ in LAG-3+/CD49+/DR4-$COL2_{259-273}$+/CD44+/CD4+ T cells. Results are expressed as mean ± SEM. Each dot represents an individual mouse. Statistics were determined with a two-tailed Mann-Whitney test. Control, DR4-CLIP.

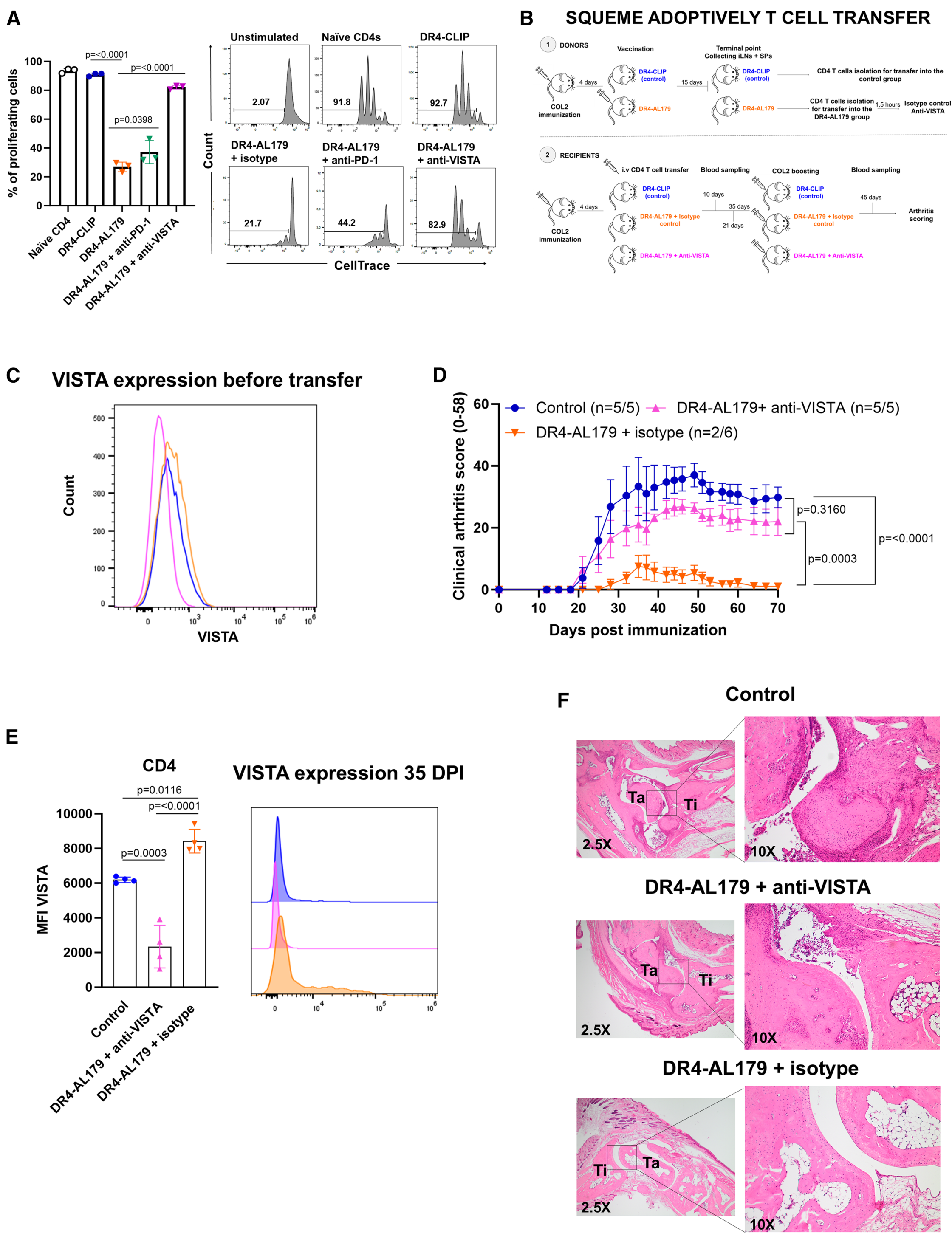

Figure 5. Impact of VISTA on the vaccine effectiveness

(A) CD4+ T cells from DR4-AL179-vaccinated Parker mice suppress anti-CD3/CD28-induced polyclonal T cell proliferation. Parker mice were immunized with COL2/CFA, and at 4 DPI mice were vaccinated with DR4–peptide complexes (DR4-CLIP or DR4-AL179) by 3 s.c. injections. 15 DPI splenocytes and dLN cells were recovered and CD4+ T cells were purified and co-cultured with purified violet CellTrace-labeled CD4+ T cells from naive (non-COL2-immunized) Parker mice. CD4+ T cells from DR4-AL179-vaccinated mice were incubated for 1 h at 4°C with 100 μg/mL anti-VISTA, anti-PD-1, or isotype control antibodies. Afterward, cells were washed and added to the naive Parker T cells. Flow cytometry was performed 3 days after stimulation with anti-CD3/CD28 antibodies. Quantification (left) and representative gating (right) are shown. (B)

(legend continued on next page)

demonstrates that the DR4-AL179 vaccine, with a unique synthetic modification of the non-glycosylated COL2 peptide, represents a promising therapeutic approach for treating autoimmune arthritis. The vaccine's ability to induce tolerance through multiple mechanisms, particularly the enrichment of VISTA-expressing regulatory T cells, suggests its potential for robust therapeutic efficacy. These findings suggest that precise synthetic modifications can augment the tolerogenic capacity of naturally occurring regulatory peptides. Since galactosylation of the peptide is no longer required, scaling up production for human therapy becomes more feasible, as it eliminates the challenges associated with ensuring the correct degree of galactosylation and purity. By deepening our knowledge of this strategy, we aim to develop more effective therapeutic approaches, potentially resulting in better patient outcomes for autoimmune diseases.

MATERIALS AND METHODS

Mice

Humanized mice (B6N.DRA.DRB1*04:01.hIi.*Cia9i*) with a knockin of the human MHCII allele DRB1*04:01 (Parker, in short), were generated as previously described,[14] and founders were provided by Vacara (Vacara.se)

In our experiments, 8- to 10-week-old male and female mice were used for phenotype and DTH experiments, while 12- to 14-week-old male and female mice were used for the CIA model. All experiments were performed with age- and sex-matched mice in a blinded fashion. Mice were kept under specific-pathogen-free (SPF) conditions in the animal facility of the Section for Medical Inflammation Research, Karolinska Institute, Stockholm, Sweden. Animals were housed in individually ventilated cages with wood shavings in a climate-controlled environment with a 14-h light-dark cycle and provided with standard chow and water *ad libitum*. All mice were healthy, with no effects on basic physiological parameters. The sample size for each experiment was determined following the reduce, refine, replace (3R) principles framework, ensuring the minimum required for statistical power while prioritizing ethical considerations. Experimental procedures were approved by the ethical committees in Stockholm, Sweden. Ethical permit numbers were 12923/18 and N134/13 (genotyping and serotyping), N35/16, and 2660/2021 (DTH, CIA).

Synthesis of COL2-peptides with and without glycosylation

The 10 glycopeptides and their counterparts without glycosylation were designed based on previous results[57] and synthesized and purified following the solid-phase methodology described elsewhere.[57,62] The purity of the glycopeptides and their counterparts was determined to be >95% according to analytical reversed-phase high-performance liquid chromatography (HPLC), and their identities were confirmed by MALDI-TOF mass spectrometry.

Vaccine constructs synthesis and purification

The sequence of the DRB1*04:01/hCLIPmut construct is shown in Figure S10. The native leader sequences of the alpha and beta chains were replaced by a *Drosophila* BiP protein signal sequence. The extracellular domains of DRB1*04:01 were truncated and the transmembrane regions were replaced by an acidic and basic zipper dimerization motif, respectively. At the C terminus of the alpha chain, a 15-amino acid AviTag peptide for site-specific biotinylation was inserted after the basic zipper, whereas a polyhistidine tag was inserted after the acidic zipper at the C terminus of the beta chain. For the DRB1*04:01/hCLIPmut construct, a low-affinity peptide hCLIPmut (PVSKARMATGALAQA) followed by a thrombin cleavage site was added to the N terminus of the beta chain.

The genes were synthesized at Eurofins with KpnI and XhoI restriction sites at the 5′ and 3′ ends. The synthesized genes were restriction enzymes digested using FastDigest enzymes (Thermo Fisher Scientific). The digested DNA fragments were cloned into a mammalian expression vector pcDNA3.4 (Life Technologies) that was digested using the same restriction enzymes. After sequence verification, the recombinant plasmids were co-transfected into Expi293F cells (Life Technologies) with FectoPRO DNA transfection reagent (Polyplus transfection). The supernatants were harvested 6 days post transfection. The recombinant protein was first captured using a 5-mL HisTrap Excel (Cytiva) affinity column followed by size-exclusion chromatography on Superdex 200 pg (Cytiva). The recombinant protein was purified as a single peak, concentrated and diafiltrated into PBS to be used as an active vaccine component.

For peptide replacement, thrombin (Novagen, 1 unit of thrombin to 1 mg of purified protein) and the desired COL2 peptide in excess was added to replace the covalently linked hCLIPmut peptide. The reaction mixture was incubated at ambient temperature overnight with slow end-to-end rotation followed by incubation at 4°C for 2 days. Cleaved peptide and additional excess desired peptide were removed by size-exclusion chromatography on a Superdex 200 pg column.

Experimental procedure of the adoptive T cell transfer. Donor Parker mice were immunized with COL2/CFA i.d. and, 4 days later, the mice were vaccinated s.c. with DR4-peptide complexes (DR4-CLIP or DR4-AL179). At 15 DPI, donor mice were terminated, and their dLNs and spleens were collected. CD4+ T cells were isolated using MACS. CD4+ T cells from DR4-AL179-vaccinated mice were divided into two groups and incubated for 1.5 h with either 100 μg/mL of isotype control or anti-VISTA antibodies. These CD4+ T cells were then transferred i.v. into the recipient mice, which had been immunized i.d. with 100 μg of bCOL2 in CFA 4 days prior. At 35 DPI, the recipient mice were boosted with 50 μg of bCOL2 in IFA i.d. Blood samples were collected at 10, 21, 35, and 45 DPI and at the terminal point. Arthritis severity was scored throughout the experiment. (C–F) Adoptive transfer of CD4+ T cells from DR4-AL179-vaccinated mice transferred into COL2-immunized recipient Parker mice. (C) The expression of VISTA in CD4+ T cells within the different groups was assessed by flow cytometry before transfer. (D) Arthritis clinical score in recipient mice after T cell transfer. The numbers of arthritic mice/total number of mice are shown. (E) The expression of VISTA in CD4+ T cells within the different groups was assessed by flow cytometry after 35 DPI in peripheral blood. (F) Representative histological examination by H&E staining of the tibia (Ti) and talus (Ta) assessment in recipient mice. Results are expressed as mean ± SEM. Each dot represents an individual mouse. Statistics were determined by using two-way ANOVA with Sidak's multiple-comparisons test. Control, DR4-CLIP.

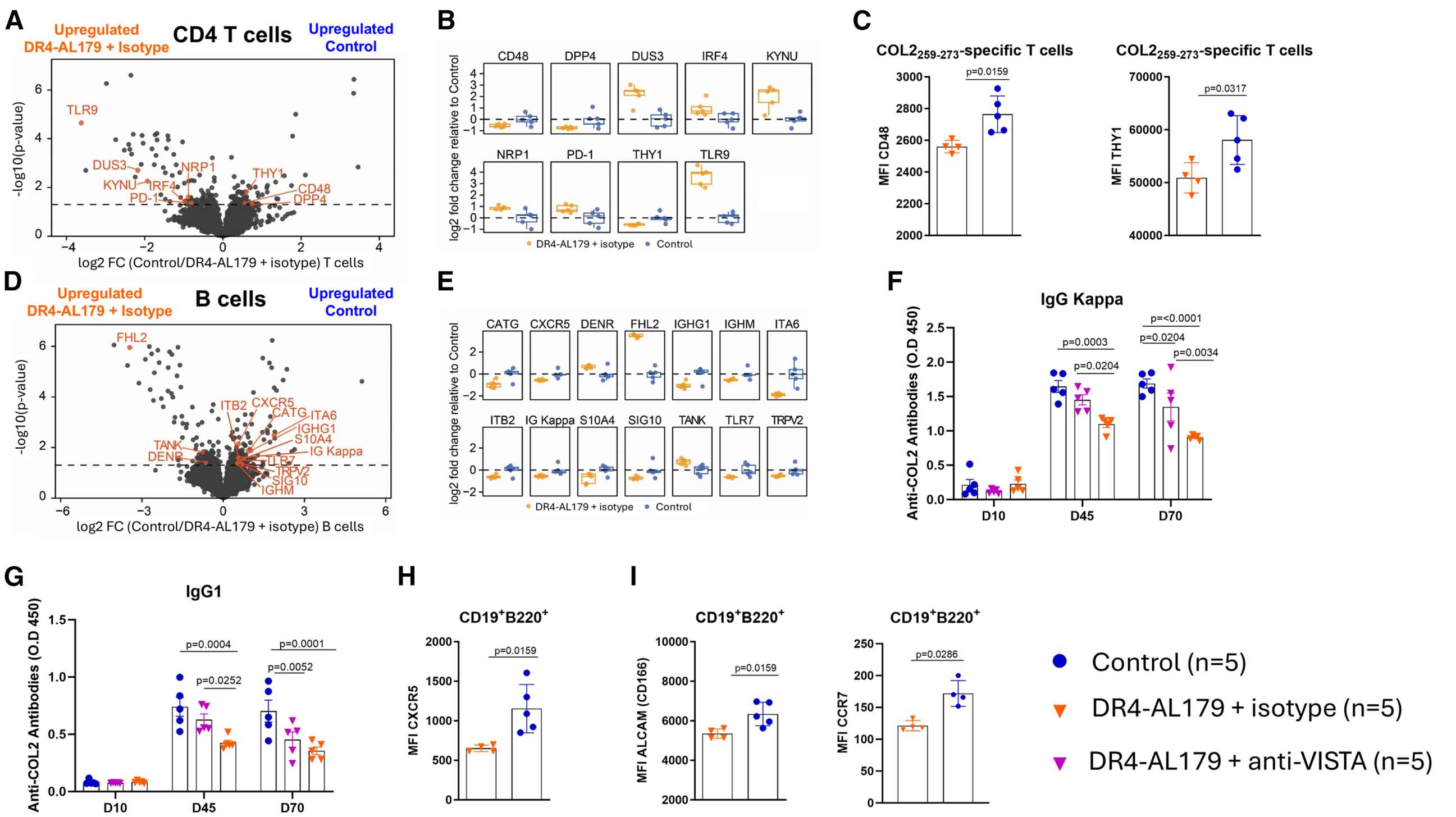

Figure 6. Impact of tolerogenic vaccination on T and B cell proteomic landscape

(A) Volcano plot comparing differently expressed proteins on sorted CD4+ T cells between DR4-AL179 isotype (left, n = 5) and DR4-CLIP (right, n = 5) adoptively transferred mice. (B) Boxplots showing selected differentially expressed proteins comparing sorted CD4+ T cells from DR4-AL179 isotype and DR4-CLIP (n = 5 each) adoptively transferred mice. The horizontal line in the boxplots represents the median, 25th, and 75th percentiles and the whiskers represent measurements to the 5th and 95th percentiles. (C) CD48 and THY1 MFI on COL2-specific T cells 15 DPI in COL2-immunized Parker mice. (D) Volcano plot comparing differently expressed proteins on B cells between DR4-AL179 isotype (left, n = 5) and DR4-CLIP (right, blue, n = 5) adoptively transferred mice. (E) Boxplots showing selected differentially expressed proteins comparing sorted B cells from DR4-AL179 isotype and DR4-CLIP (n = 5 each) adoptively transferred mice. The horizontal line in the boxplots represents the median, 25th, and 75th percentiles and the whiskers represent measurements to the 5th and 95th percentiles. (F- and G) Anti-COL2 antibody levels of total IgG (anti-kappa) and of IgG1, respectively, from adoptively transferred mice at different time points measured by ELISA. (H) CXCR5 MFI on B cells 15 DPI in COL2-immunized Parker mice. (I) ALCAM and CCR7 MFI on B cells 15 DPI in COL2-immunized Parker mice. Results are expressed as mean ± SEM. Each dot represents an individual mouse. Statistics in (C), (H), and (I) were determined with a two-tailed Mann-Whitney test and in (F) and (G) were determined by using two-way ANOVA with Sidak's multiple-comparisons test. Control, DR4-CLIP.

Peptide competitive-binding assay

For peptide competitive-binding assay, a 0.2 μM solution of purified DRB1*04:01-hCLIPmut complex and 6 μM Biotin-hCLIP peptide was incubated at room temperature (RT) for 48 h. Various concentrations of COL2 peptides and influenza hemagglutinin peptide ($HA_{306\text{-}318}$) as a positive control were added as competitors to Biotin-Ahx-hCLIP peptide. Bound peptides were separated from free peptides by immobilizing the DRB1*04:01 molecules on microtiter plates coated with the anti-human HLA-DR (L243, in-house) antibody and subsequently washed with PBS. The L243 antibody was adhered to the plate by an overnight incubation of a 2 μg/mL solution at 4°C. Bound biotinylated peptides were detected by incubations with streptavidin-europium followed by a chelating enhancement solution. Fluorescence was quantitated using a Synergy 2 plate reader (BioTek Instrument), and data are expressed as relative fluorescence units (RFU) measured. Each binding assay was performed in duplicate, and the data are representative of three experiments.

In vitro evaluation of T cell activation by glycopeptides

Spleen cells (APCs) prepared from naive Parker mice were resuspended in complete Dulbecco's modified Eagle's medium (DMEM) containing DMEM + GlutaMAX (Gibco), 5% heat-inactivated fetal bovine serum (FBS) (Gibco), 10 μM HEPES (Sigma), 100 μg/mL streptomycin sulfate (Sigma), 100 IU/mL penicillin C (Sigma), and 50 μM β-mercaptoethanol (Gibco). Splenocytes (5×10^5) were co-cultured with three different clones (mDR1.1, mDR17.2, and hDR11.2) of DR4-restricted T cell hybridoma cells specific for the galactosylated $COL2_{259\text{-}273}$ epitope (5×10^4) in a total volume of 200 μL in U-bottom 96-well plates. The different glycopeptides were titrated (0.15–37.5 μM), while the amounts of T-cell hybridoma and APCs were kept constant. As negative controls, T-cell hybridomas incubated with APCs in only medium were used. After 24 h cultured at 37°C, supernatants were collected to assess antigen presentation by measuring the IL-2 levels by ELISA. Establishment of T cell hybridoma clones and determination of antigen specificity was described previously.[19]

In vitro evaluation of T cell activation by MHCII-peptide complexes

Stimulation experiments of T-hybridoma cells were performed in 96-well plates with precoating of MHCII-peptide complexes at different concentrations of between 10 and 100 μg/mL in PBS overnight. The coated wells were then washed twice with sterile PBS. Subsequently, 1×10^5 murine T-hybridoma cells (3H8) specifically recognizing native COL2 in a DRB1*04:01-restricted manner were added in 200 μL of DMEM supplemented with 5% fetal calf serum (FCS), 100 IU/mL penicillin, and 100 μg/mL streptomycin. T cell activation by the MHCII-peptide complexes was assessed by IL-2 release into the medium (Sandwich IL-2 ELISA, BioLegend).

IL-2 ELISA

Flat 96-well plates (Maxisorp, Nunc) were coated overnight at 4°C with the IL-2 capture antibody (5 μg/mL JES6-IA12, in-house produced) in PBS. Supernatants from cell culture were added and plates were incubated for 2 h at RT before washing (PBS-Tween 0.05%) and adding the biotinylated detection antibody (2 μg/mL JES6-5H4, in-house produced) for 1 h at RT. Plates were washed and incubated for 30 min at RT with Eu-labeled streptavidin (PerkinElmer; 1:1,000) in buffer E (50 μM Tris-HCl, 0.9% [wt/vol] NaCl, 0.5% [wt/vol] BSA, 0.1% Tween 20, 2 mM EDTA). After washing, DELFIA Enhancement Solution (PerkinElmer) was added, and fluorescence was read at 620 nm (Synergy 2, BioTek).

Vaccination protocol

DR4-peptide complexes (100 μg) were administered using one of two methods: via micro-osmotic pumps (DURECT Corporation; ALZET Model 2001D, delivering 8 μL/h for a 1-day duration, reservoir capacity 200 μL) or through three s.c. injections administered every 4 h. For the placement of s.c. pumps, a minor incision was made to create an s.c. pocket where the micro-pumps were inserted with the flow moderator oriented away from the incision site. Post-surgical analgesia was provided by s.c. injection of buprenorphine (e.g., Temgesic; Indivior Europe) at a dosage of 0.2 mg/kg. Mice received vaccinations on both day 4 and day 32 post immunization.

DTH

Parker mice were sensitized by intradermal (i.d.) injection of 100 μg of bovine COL2[63] in 100 μL of a 1:1 emulsion with complete Freund's adjuvant (CFA; BD, Difco) and 10 mM acetic acid at the base of the tail. Day 8 after sensitization, the right ear was injected i.d. with 10 μL of COL2 in acetic acid (1 mg/mL) while the control left ear was injected with 10 μL of acetic acid in PBS. Ear swelling response was measured during 0, 24, 48, and 72 h after the challenge using a caliper. Ear swelling was calculated by subtracting the thickness of the acetic acid-injected ear from the thickness of the COL2-injected ear normalized to day 0 thickness.[64]

CIA

Parker mice were immunized with 100 μg of bovine COL2 in 100 μL of a 1:1 emulsion with CFA and 10 mM acetic acid (mixed by an emulsifier machine, BTB POWER-Kit)[65] injected i.d. at the base of the tail. Mice were challenged on day 35 with 50 μg of COL2 in 50 μL of incomplete Freund's adjuvant (IFA) (BD, Difco) emulsion injected i.d. The progression of arthritis was assessed by visually inspecting the paws employing a macroscopic scoring system. In this scoring system (0–58), visibly inflamed ankles or wrists were assigned five points each, while inflamed toes or fingers were accorded one point each. During the CIA disease course, blood samples were collected through the cheek-bleeding technique at different time points.

Adoptive T cell transfer

The transfer experiment depicted in Figure 3I was conducted using DR4-CLIP- and DR4-AL179-treated Parker donor male mice, which were sacrificed 15 DPI. Untouched CD4+ T cells were isolated from the spleens and lymph nodes using magnetic-activated cell sorting (MACS) in accordance with the manufacturer's instructions (Miltenyi Biotec, catalog no. 130-104-45). A total of 5×10^6 CD4+ T cells were adoptively transferred intravenously (i.v.) into COL2-immunized Parker mice 4 DPI. The transfer experiment shown in Figures 5C–5F, included a secondary step where CD4+ T cells from DR4-AL179-vaccinated donor mice were divided into two groups. These cells were incubated for 1.5 h with either 100 μg/mL of neutralizing anti-VISTA antibody (clone 13F3) or an anti-isotype control (all from Bio X Cell) before being transferred i.v. into COL2-immunized Parker mice 4 days post immunization.

The purity of the CD4+ T cells, as determined by flow cytometry, was greater than 85%.

ELISA and bead-based multiplex flow immunoassay for determination of anti-COL2 antibody titers and epitope specificity

Serum samples were used for the analysis of the COL2-specific IgG antibody response by ELISA previously described in Romero-Castillo et al.[14] or for the analysis of epitope specificity of the IgG antibody response using peptides derived from COL2 assayed on multiplex bead-based flow immunoassay on the Luminex platform, as described previously.[66]

Detection of anti-COL2 U1 epitope IgG antibodies by ELISA

Flat 96-well plates (Maxisorp, Nunc) were coated with Neutravidin (Thermo Fisher Scientific; 5 μg/mL in PBS) overnight at 4°C. The following day, plates were washed with PBS containing 0.05% Tween 20 and incubated with biotin-conjugated triple-helical COL2 peptide containing the U1 epitope (1 μg/mL in PBS)[67] for 30 min at RT. After blocking with 1% BSA in PBS for 1 h at RT, diluted serum samples (1:2,000) were added and incubated for 2 h at RT. Plates were then washed with PBS-Tween 0.05% and incubated with HRP-conjugated anti-mouse IgG detection antibody (1:4,000 dilution; Southern Biotech) for 1 h at RT. Following a final wash step, 50 μL of 3,3′,5,5′-tetramethylbenzidine (TMB) substrate (Sigma) was added to each well. The reaction was stopped by adding 50 μL of 0.3 M H_2SO_4, and absorbance was measured at 450 nm using a Synergy 2 plate reader (BioTek).

Cytokine and chemokine assay

Serum cytokines and chemokines within the CIA model were detected 15 days post immunization using a Mouse Cytokine Array/Chemokine Array 44-Plex (Eve Technologies, Calgary, AB, Canada).

Preparation of cell suspension from lymphoid organs

Spleens or inguinal lymph nodes were mashed using a 1-mL syringe plunger on a 40-μm cell strainer (Falcon). The cell suspension was washed once in PBS and centrifuged at 350 × *g* for 5 min at RT. For spleen, red blood cells (RBCs) were lysed in 1 mL of RBC lysis buffer for 1–2 min at RT (0.155 M NH_4Cl, 120 mM $NaHCO_3$, 100 μM EDTA). Thereafter, cells were washed in PBS and carefully transferred to a new 15-mL tube to get rid of debris. Cells were centrifuged and resuspended in 1–3 mL of DMEM (Gibco, Invitrogen) for counting in a Sysmex KX-21N cell counter.

Ex vivo T cell-suppression assays

Flat-bottom 96-well plates were pre-coated with anti-CD3 antibody (2 μg/mL, 500A2, BD Pharmingen) in PBS overnight at 4°C. CD4+ T cells were isolated from the spleens and lymph nodes of DR4-CLIP, DR4-AL179-vaccinated Parker mice and naive Parker mice using negative selection (Dynal Mouse CD4 Negative Isolation Kit, Thermo Fisher Scientific). The cells were resuspended in complete RPMI 1640 with GlutaMAX (Thermo Scientific) containing 50 μg/mL streptomycin sulfate (Sigma), 60 μg/mL penicillin C (Sigma), 10% heat-inactivated FBS (Thermo Scientific), 50 μM β-mercaptoethanol (Thermo Scientific), and 10 μM HEPES (Sigma).

The isolated CD4+ T cells from naive Parker mice were washed and labeled with the fluorescent dye CellTrace violet (Invitrogen) according to the manufacturer's protocol. Violet-labeled CD4+ T cells (1×10^5) from naive mice and 3×10^5 CD4+ T cells from treated mice were added to the pre-coated plates. The soluble form of hamster anti-mouse CD28 antibody (0.5 μg/mL, 37.51, BD) was also added to each well. Cells were incubated at 37°C in a 5% CO_2 atmosphere.

To investigate the role of immune checkpoint molecules in vaccine suppression, we blocked specific molecules by incubating CD4+ T cells from vaccinated mice for 1.5 h with 100 μg/mL of neutralizing anti-VISTA (clone 13F3), anti-PD-1 (clone RMP1-14), or an isotype control antibody (all from Bio X Cell) before being co-cultured with violet-labeled CD4+ T from naive mice. The dilution of CellTrace in responder cells was assessed 72 h later using flow cytometry.

ELISpot

For T cell recall assays, 1×10^6 lymph node cells per/well were plated in anti-IL17A (TC11-18H10.1, 5 μL, Mabtech)-coated plates (Merck Millipore, #MSIPS4W10), stimulated with denatured bCOL2 (30 μg/mL), and incubated for 48 h at 37°C. Positive controls consisted of lymph node cells stimulated with Concanavalin A (ConA; 1 μg/mL, Sigma), while negative controls comprised unstimulated cells in complete medium only. Bound IL-17A was detected with anti-IL17A (TC11-8H4, 1 μg/mL, Mabtech) followed by streptavidin-conjugated alkaline phosphatase. The spots were developed using the substrate BCIP/Nitroblue Tetrazolium (Sigma). Scanned wells (ImmunoScan) were analyzed with ImmunoSpot software (Cellular Technology).

Synthesis of DRB1*04:01-$COL2_{259\text{-}273}$ tetramer complexes for flow cytometry

For preparation of the DRB1*04:01-$COL2_{259\text{-}273}$ tetramer complexes, the proteins were transferred to a biotinylation buffer (20 mM Tris-HCl, 50 mM NaCl, pH 8.0) using a centrifuge device with molecular weight cut off (MWCO) of 10 kDa (Cytiva AB). Biotinylation using biotin-protein ligase was performed according to the manufacturer's instructions (Avidity, Denver, CO) and the reaction was carried out at 30°C for 2 h. Following biotinylation, thrombin (Novagen, 1 unit of thrombin to 1 mg of purified protein) and the $COL2_{259\text{-}273}$ peptide in excess were added to replace the covalently linked hCLIPmut peptide. The reaction mixture was incubated at ambient temperature overnight with slow end-to-end rotation followed by incubation at 4°C for 2 days. Free biotin, cleaved peptide, and additional excess desired peptide were removed by size-exclusion chromatography on a Superdex 200 pg column.

Flow cytometry and staining and enrichment of DR4-$COL2_{259\text{-}273}$ specific cells

Single-cell suspensions from spleens and inguinal lymph nodes were obtained as previously described above. All centrifugation steps were carried out at 350 × *g* for 5 min at RT. To block Fc receptors, the cells were incubated in 25 μL of FACS buffer (2% FCS in PBS) containing 10 μg of anti-CD16/CD32 monoclonal antibody (mAb) (2.4G2; in-house produced) in 96-well plates for 15 min at 4°C. Samples were washed with 150 μL of PBS and subsequently stained with the indicated antibodies diluted 1:200 or 1:300 in 50 μL of FACS buffer at 4°C for 20 min in the dark. Cells were washed once, fixed, and permeabilized for intracellular staining using BD Cytofix/Cytoperm (BD Biosciences) according to the manufacturer's instructions and stained with antibodies diluted at 1:200 in 50 μL of permeabilization buffer (BD Biosciences) for 30 min at RT.

DR4-$COL2_{259\text{-}273}$ tetramer was prepared by mixing the biotinylated MHCII-peptide complexes with streptavidin R-phycoerythrin (Thermo Fisher Scientific, Eugene, OR, USA) at 4:1 molar ratio.[68] To detect DR4-$COL2_{259\text{-}273}$-specific cells, splenocytes and inguinal lymph node cells were first incubated with an anti-CD16/CD32 mAb and 50 nM dasatinib (Santa Cruz Biotechnology, Santa Cruz, CA, USA) for 30 min at 37°C. Then the cells were stained with DR4-$COL2_{259\text{-}273}$ tetramer (20 μg/mL) in DMEM containing 5% FBS (Gibco, Invitrogen) in the presence of 50 nM dasatinib for 1 h at 37°C. Enrichment of DR4-$COL2_{259\text{-}273}$ tetramer-specific cells was performed using anti-R-Phycoerythrin magnetic beads (Miltenyi Biotec) according to the previously described protocol.[69] Subsequent surface and intranuclear staining were carried out as indicated above.

Antibodies

All the antibodies used in this study were purchased from BD Bioscience, BioLegend, Miltenyi, or Invitrogen. Anti-mouse antibodies and clones used were the following: anti-CD4 (RM4-5), anti-CD44 (IM7), anti-PD-1 (RMP1-30), anti-FR4 (eBio12A5), anti-CD73 (eBioTY/11.8), anti-VISTA (MIH64), anti-FOXP3 (FJK-16s), anti-CD49 (DX5), anti-LAG-3 (C9B7W), anti-B220 (RA3-6B2), anti-CD11b (M1/70), anti-IA/IE (M5/114.15.2), anti-CD19 (6D5), anti-CD48 (HM48-1), anti-THY1 (S20007C), anti-NRP1 (3E12), anti-CCR7 (4B12), anti-ALCAM (RRA270), anti-4RA (I015F8), anti-CXCR5 (L138D7), anti-TLR9 (S18025A), and anti-TLR7 (A94B10). Anti-human HLA-DR (L243) was also used. Live/dead fixable dyes for discrimination of dead cells were purchased from Thermo Fisher and three different formats were used: green, near-infrared, and violet.

Samples were acquired with Attune NxT flow cytometer (Thermo Fisher Scientific) and analyzed with FlowJo version 10.7.2.

Histology

At the end of the CIA and transfer experiments, mice were euthanized, and their skinless hind paws were collected. The hind paws were fixed in 10% phosphate-buffered formaldehyde for 15 days. Following fixation, the tissues were decalcified for 4–5 weeks in a decalcification buffer consisting of 10% EDTA, 7.5% polyvinylpyrrolidone, and 0.1 M Tris-HCl (pH 6.95).

After decalcification, the tissues were dehydrated in 70% ethanol and then embedded in paraffin. Tissue sections were subsequently stained with hematoxylin and eosin (H&E). The sectioning and H&E staining were performed by Histocore at the Karolinska Institutet, Sweden.

Proteomic sample preparation

At the terminal point of the transfer experiment, where CD4+ T cells from DR4-AL179 treated mice were incubated with either anti-VISTA or an isotype control before transfer into COL2-immunized recipient mice, both spleens and draining lymph nodes (dLNs) were subjected to FACS of both CD4+ T and B cells. Following FACS, the sorted cells were pelleted and stored at −20°C until proteomic sample preparation. Subsequently, sorted T and B cells were resuspended in 50 mM Tris pH 8.5 and 1% sodium deoxycholate, and the proteins were denatured at 95°C for 10 min. Subsequently, the protein amount was determined by bicinchoninic acid assay (BCA) (Thermo Fisher Scientific) following the manufacturer's protocol. Individual samples were reduced with 5 mM DTT for 1 h followed by alkylation with 15 mM iodoacetamide in the dark for another hour. Samples were then digested with LysC endopeptidase (an enzyme-to-protein ratio of 1:100 w/w) for 2 h, followed by digestion using the same amount of trypsin overnight. Samples were desalted by StageTip (Thermo Fisher Scientific) following the manufacturer's protocol. Cleaned samples were dried in a speed vac and stored at −80°C.

Mass spectrometry analysis

Samples were resuspended in buffer A (2% ACN, 0.1% FA) and injected into an UltiMate 3000 UPLC autosampler (Thermo Fisher Scientific), which was connected to an Orbitrap Fusion Lumos Tribrid mass spectrometer (Thermo Fisher Scientific). The peptides were loaded on a trap column (Acclaim PepMap 100 C18, 100 μm × 2 cm) and subsequently separated on a 50-cm-long C18 Easy spray column (Thermo Fisher Scientific). Chromatographic separation of the peptides was achieved by the following 105-min gradient: 4%–20% of solvent B (98% ACN and 0.1% FA) in 80 min, to 26% in 10 min, to 95% in 3 min where it was kept for another 3 min, before going to 4% in 1 min and held for 8 min. During the entire gradient, the mass spectrometer was operating in positive polarity. All mass spectra were acquired in profile mode using the Orbitrap analyzer. An acquisition cycle consisted of one survey mass spectrum acquired at a mass resolution of 120,000 from *m/z* 395 to 1,005, with an automated gain control (AGC) target of 1,000,000 charges and a maximal injection time of 150 ms. This was followed by 40 data independent acquisition (DIA) MS/MS events with variable isolation windows. The individual mass spectra were recorded at a resolution of 30,000 with 28% NCE; stepped normalized collision energy (NCE) was set as 2%, and maximal injection time was 54 ms. A library of MS/MS spectra was created with a pooled sample. The data acquisition was the same as for the individual samples with the exception that the DIA MS/MS spectra were recorded over a mass range of 120 *m/z* at a resolution of 60,000 with a maximum injection time of 54 ms and an overlap between MS/MS windows of 4 *m/z* units.

Bioinformatics and proteomic data analysis

Acquired.raw files were converted to mzML format by MSConvert (version 3.0.21258)[70] applying peak picking in the mass spectra with the vendor-provided algorithm (Thermo Fisher Scientific). The database search was performed in FragPipe (v18.0) as described in Yu et al.[71] using the mouse Swissprot database (17,248 entries). Trypsin with up to one missed cleavage was set as a digestion enzyme, and oxidation of methionine and acetylation of the N terminus were set as variable modifications. Carbamidomethylation of cysteine residues was set as a fixed modification. Peptide length was restricted to 7–50 amino acids, and molecular mass from 500 to 5000 Da. The resulting peptide-spectrum matches were adjusted to a 1% false discovery rate with Percolator[72] as part of the Philosopher toolkit (v4.4)[73] and converted to an MS/MS spectra library. DIA files were analyzed by DIA-NN 1.8.1.[74]

All further data processing was performed in R (version 4.2.2). Known contaminants and decoy proteins were excluded from further analysis. Only proteins quantified in three out of the five replicates in at least one sample group were considered. Protein abundances were normalized by variance stabilization normalization approach and missing values at random were imputed by k-nearest neighbor method, whereas missing values not at random were imputed by drawing values from a downshifted normal distribution.[75] All statistical comparisons were performed based on a two-tailed Student's t test with equal variances; the differences between the comparisons were reported as log2-scaled fold changes.

DATA AVAILABILITY

The data supporting the findings of this study are available within the paper (supplemental information). The mass spectrometry proteomics data have been deposited to the ProteomeXchange Consortium (http://proteomecentral.proteomexchange.org) via the PRIDE partner repository[76] with the dataset identifier PXD056818. All other data and the unique reagents used in this study are available from the corresponding author upon reasonable request.

ACKNOWLEDGMENTS

This work was supported by grants from Vetenskapsrådet (2024-02575), Novo Nordisk Fund (NNF24OC0090035), the Knut and Alice Wallenberg Foundation (2019.0059), and the Swedish Association against Rheumatism. L.R.-C. received support from the Karolinska Institutet foundation for Rheumatology Research (2020-02541, 2021-01145, 2022-02857, 2023-02645, 2024-03851), the Ulla and Gustaf af Uggla Foundation (2021-01015), the Åke Wiberg Stiftelse (M21-0148; M24-0077), the Lars Hiertas Minne Foundation (F02023-0284), the Reumatikerförbundet Association (R995628), and the Konung Gustaf V:s (SGI-2023-0993) and got the Margarita Salas grant from the Spanish Ministry of Universities through the European Union (NextGeneration EU). The humanized mice were provided by Vacara AB. We would like to thank the Histological Core Facility in Biomedicum, especially Emma Mondoc, for performing the histology shown in this manuscript. Finally, we would like to thank all KM-A technicians, especially Carlos Palestro and Martina Andersson, for taking care of the animals.

AUTHOR CONTRIBUTIONS

All authors were involved in drafting the article or revising it critically for important intellectual content, and all authors approved the final version to be published.

L.R.-C., V.U., O.S., H.B., J.K., R.A.Z., J.K., A.L., and R.H., designed research. L.R.-C., R.K.P., B.X., C.M.B., A.O.-C., K.Z., C.S., Z.X., H.L., P.S., L.C., A.K., C.L., and S.H. performed research. L.R.-C., R.K.P., C.M.B., C.G., and S.H. analyzed data. L.R.-C. and R.H. wrote the paper.

DECLARATION OF INTERESTS

R.H. is the founder of Vacara AB. Z.X. and O.S. are employees of Vacara AB.

SUPPLEMENTAL INFORMATION

Supplemental information can be found online at https://doi.org/10.1016/j.ymthe.2025.04.034.

REFERENCES

1. Zhao, M., Zhai, H., Li, H., Wei, F., Ma, H., Liu, Y., Li, W., and Wei, P. (2024). Age standardized incidence, prevalence, and mortality rates of autoimmune diseases in adolescents and young adults (15-39 years): an analysis based on the global burden of disease study 2021. BMC Public Health *24*, 1800. https://doi.org/10.1186/s12889-024-19290-3.
2. Gutierrez-Arcelus, M., Rich, S.S., and Raychaudhuri, S. (2016). Autoimmune diseases - connecting risk alleles with molecular traits of the immune system. Nat. Rev. Genet. *17*, 160–174. https://doi.org/10.1038/nrg.2015.33.
3. Cooper, G.S., Bynum, M.L.K., and Somers, E.C. (2009). Recent insights in the epidemiology of autoimmune diseases: improved prevalence estimates and understanding of clustering of diseases. J. Autoimmun. *33*, 197–207. https://doi.org/10.1016/j.jaut.2009.09.008.
4. Smolen, J.S., Aletaha, D., and McInnes, I.B. (2016). Rheumatoid arthritis. Lancet *388*, 2023–2038. https://doi.org/10.1016/S0140-6736(16)30173-8.
5. Sparks, J.A. (2019). Rheumatoid Arthritis. Ann. Intern. Med. *170*, ITC1–ITC16. https://doi.org/10.7326/AITC201901010.
6. Almutairi, K., Nossent, J., Preen, D., Keen, H., and Inderjeeth, C. (2021). The global prevalence of rheumatoid arthritis: a meta-analysis based on a systematic review. Rheumatol. Int. *41*, 863–877. https://doi.org/10.1007/s00296-020-04731-0.
7. Finckh, A., Gilbert, B., Hodkinson, B., Bae, S.C., Thomas, R., Deane, K.D., Alpizar-Rodriguez, D., and Lauper, K. (2022). Global epidemiology of rheumatoid arthritis. Nat. Rev. Rheumatol. *18*, 591–602. https://doi.org/10.1038/s41584-022-00827-y.
8. Smolen, J.S., Aletaha, D., Barton, A., Burmester, G.R., Emery, P., Firestein, G.S., Kavanaugh, A., McInnes, I.B., Solomon, D.H., Strand, V., et al. (2018). Rheumatoid arthritis. Nat. Rev. Dis. Primers *4*, 18001. https://doi.org/10.1038/nrdp.2018.1.
9. Okada, Y., Wu, D., Trynka, G., Raj, T., Terao, C., Ikari, K., Kochi, Y., Ohmura, K., Suzuki, A., Yoshida, S., et al. (2014). Genetics of rheumatoid arthritis contributes to biology and drug discovery. Nature *506*, 376–381. https://doi.org/10.1038/nature12873.
10. Raychaudhuri, S., Sandor, C., Stahl, E.A., Freudenberg, J., Lee, H.S., Jia, X., Alfredsson, L., Padyukov, L., Klareskog, L., Worthington, J., et al. (2012). Five amino acids in three HLA proteins explain most of the association between MHC and seropositive rheumatoid arthritis. Nat. Genet. *44*, 291–296. https://doi.org/10.1038/ng.1076.
11. Aoun, M., Coelho, A., Kramer, A., Saxena, A., Sabatier, P., Beusch, C.M., Lonnblom, E., Geng, M., Do, N.N., Xu, Z., et al. (2023). Antigen-presenting autoreactive B cells activate regulatory T cells and suppress autoimmune arthritis in mice. J. Exp. Med. *220*, e20230101. https://doi.org/10.1084/jem.20230101.
12. Burkhardt, H., Sehnert, B., Bockermann, R., Engström, A., Kalden, J.R., and Holmdahl, R. (2005). Humoral immune response to citrullinated collagen type II determinants in early rheumatoid arthritis. Eur. J. Immunol. *35*, 1643–1652. https://doi.org/10.1002/eji.200526000.
13. Holmdahl, R., Andersson, M., Goldschmidt, T.J., Gustafsson, K., Jansson, L., and Mo, J.A. (1990). Type II collagen autoimmunity in animals and provocations leading to arthritis. Immunol. Rev. *118*, 193–232. https://doi.org/10.1111/j.1600-065x.1990.tb00817.x.
14. Romero-Castillo, L., Li, T., Do, N.N., Sareila, O., Xu, B., Hennings, V., Xu, Z., Svensson, C., Oliveira-Coelho, A., Sener, Z., et al. (2024). Human MHC Class II and Invariant Chain Knock-in Mice Mimic Rheumatoid Arthritis with Allele Restriction in Immune Response and Arthritis Association. Adv. Sci. *11*, e2401513. https://doi.org/10.1002/advs.202401513.
15. Trentham, D.E., Townes, A.S., and Kang, A.H. (1977). Autoimmunity to type II collagen an experimental model of arthritis. J. Exp. Med. *146*, 857–868. https://doi.org/10.1084/jem.146.3.857.
16. Andersson, E.C., Hansen, B.E., Jacobsen, H., Madsen, L.S., Andersen, C.B., Engberg, J., Rothbard, J.B., McDevitt, G.S., Malmström, V., Holmdahl, R., et al. (1998). Definition of MHC and T cell receptor contacts in the HLA-DR4restricted immunodominant epitope in type II collagen and characterization of collagen-induced arthritis in HLA-DR4 and human CD4 transgenic mice. Proc. Natl. Acad. Sci. USA *95*, 7574–7579. https://doi.org/10.1073/pnas.95.13.7574.
17. Rosloniec, E.F., Brand, D.D., Myers, L.K., Esaki, Y., Whittington, K.B., Zaller, D.M., Woods, A., Stuart, J.M., and Kang, A.H. (1998). Induction of autoimmune arthritis in HLA-DR4 (DRB1*0401) transgenic mice by immunization with human and bovine type II collagen. J. Immunol. *160*, 2573–2578.
18. Yu, X., Mai, Y., Wei, Y., Yu, N., Gao, T., and Yang, J. (2023). Therapeutic potential of tolerance-based peptide vaccines in autoimmune diseases. Int. Immunopharmacol. *116*, 109740. https://doi.org/10.1016/j.intimp.2023.109740.
19. Backlund, J., Carlsen, S., Hoger, T., Holm, B., Fugger, L., Kihlberg, J., Burkhardt, H., and Holmdahl, R. (2002). Predominant selection of T cells specific for the glycosylated collagen type II epitope (263-270) in humanized transgenic mice and in rheumatoid arthritis. Proc. Natl. Acad. Sci. USA *99*, 9960–9965. https://doi.org/10.1073/pnas.132254199.
20. Ge, C., Weisse, S., Xu, B., Dobritzsch, D., Viljanen, J., Kihlberg, J., Do, N.N., Schneider, N., Lanig, H., Holmdahl, R., and Burkhardt, H. (2022). Key interactions in the trimolecular complex consisting of the rheumatoid arthritis-associated DRB1*04:01 molecule, the major glycosylated collagen II peptide and the T-cell receptor. Ann. Rheum. Dis. *81*, 480–489. https://doi.org/10.1136/annrheumdis-2021-220500.
21. Freedman, M.S., Bar-Or, A., Oger, J., Traboulsee, A., Patry, D., Young, C., Olsson, T., Li, D., Hartung, H.P., Krantz, M., et al. (2011). A phase III study evaluating the efficacy and safety of MBP8298 in secondary progressive MS. Neurology *77*, 1551–1560. https://doi.org/10.1212/WNL.0b013e318233b240.
22. Warren, K.G., Catz, I., Ferenczi, L.Z., and Krantz, M.J. (2006). Intravenous synthetic peptide MBP8298 delayed disease progression in an HLA Class II-defined cohort of patients with progressive multiple sclerosis: results of a 24-month double-blind

placebo-controlled clinical trial and 5 years of follow-up treatment. Eur. J. Neurol. *13*, 887–895. https://doi.org/10.1111/j.1468-1331.2006.01533.x.

23. Capini, C., Jaturanpinyo, M., Chang, H.I., Mutalik, S., McNally, A., Street, S., Steptoe, R., O'Sullivan, B., Davies, N., and Thomas, R. (2009). Antigen-specific suppression of inflammatory arthritis using liposomes. J. Immunol. *182*, 3556–3565. https://doi.org/10.4049/jimmunol.0802972.

24. Prasad, S., Neef, T., Xu, D., Podojil, J.R., Getts, D.R., Shea, L.D., and Miller, S.D. (2018). Tolerogenic Ag-PLG nanoparticles induce tregs to suppress activated diabetogenic CD4 and CD8 T cells. J. Autoimmun. *89*, 112–124. https://doi.org/10.1016/j.jaut.2017.12.010.

25. Dzhambazov, B., Nandakumar, K.S., Kihlberg, J., Fugger, L., Holmdahl, R., and Vestberg, M. (2006). Therapeutic vaccination of active arthritis with a glycosylated collagen type II peptide in complex with MHC class II molecules. J. Immunol. *176*, 1525–1533. https://doi.org/10.4049/jimmunol.176.3.1525.

26. Urbonaviciute, V., Romero-Castillo, L., Xu, B., Luo, H., Schneider, N., Weisse, S., Do, N.N., Oliveira-Coelho, A., Fernandez Lahore, G., Li, T., et al. (2023). Therapy targeting antigen-specific T cells by a peptide-based tolerizing vaccine against autoimmune arthritis. Proc. Natl. Acad. Sci. USA *120*, e2218668120. https://doi.org/10.1073/pnas.2218668120.

27. Marek-Trzonkowska, N., Mysliwiec, M., Dobyszuk, A., Grabowska, M., Techmanska, I., Juscinska, J., Wujtewicz, M.A., Witkowski, P., Mlynarski, W., Balcerska, A., et al. (2012). Administration of CD4+CD25highCD127- regulatory T cells preserves beta-cell function in type 1 diabetes in children. Diabetes Care *35*, 1817–1820. https://doi.org/10.2337/dc12-0038.

28. Papenfuss, T.L., Powell, N.D., McClain, M.A., Bedarf, A., Singh, A., Gienapp, I.E., Shawler, T., and Whitacre, C.C. (2011). Estriol generates tolerogenic dendritic cells in vivo that protect against autoimmunity. J. Immunol. *186*, 3346–3355. https://doi.org/10.4049/jimmunol.1001322.

29. Tabansky, I., Keskin, D.B., Watts, D., Petzold, C., Funaro, M., Sands, W., Wright, P., Yunis, E.J., Najjar, S., Diamond, B., et al. (2018). Targeting DEC-205(-)DCIR2(+) dendritic cells promotes immunological tolerance in proteolipid protein-induced experimental autoimmune encephalomyelitis. Mol. Med. *24*, 17. https://doi.org/10.1186/s10020-018-0017-6.

30. ElTanbouly, M.A., Zhao, Y., Nowak, E., Li, J., Schaafsma, E., Le Mercier, I., Ceeraz, S., Lines, J.L., Peng, C., Carriere, C., et al. (2020). VISTA is a checkpoint regulator for naive T cell quiescence and peripheral tolerance. Science *367*, eaay0524. https://doi.org/10.1126/science.aay0524.

31. Snir, O., Bäcklund, J., Boström, J., Andersson, I., Kihlberg, J., Buckner, J.H., Klareskog, L., Holmdahl, R., and Malmström, V. (2012). Multifunctional T cell reactivity with native and glycosylated type II collagen in rheumatoid arthritis. Arthritis Rheum. *64*, 2482–2488. https://doi.org/10.1002/art.34459.

32. Nandakumar, K.S., Bajtner, E., Hill, L., Böhm, B., Rowley, M.J., Burkhardt, H., and Holmdahl, R. (2008). Arthritogenic antibodies specific for a major type II collagen triple-helical epitope bind and destabilize cartilage independent of inflammation. Arthritis Rheum. *58*, 184–196. https://doi.org/10.1002/art.23049.

33. Kalekar, L.A., Schmiel, S.E., Nandiwada, S.L., Lam, W.Y., Barsness, L.O., Zhang, N., Stritesky, G.L., Malhotra, D., Pauken, K.E., Linehan, J.L., et al. (2016). CD4(+) T cell anergy prevents autoimmunity and generates regulatory T cell precursors. Nat. Immunol. *17*, 304–314. https://doi.org/10.1038/ni.3331.

34. Martinez, R.J., Zhang, N., Thomas, S.R., Nandiwada, S.L., Jenkins, M.K., Binstadt, B. A., and Mueller, D.L. (2012). Arthritogenic self-reactive CD4+ T cells acquire an FR4hiCD73hi anergic state in the presence of Foxp3+ regulatory T cells. J. Immunol. *188*, 170–181. https://doi.org/10.4049/jimmunol.1101311.

35. Thomann, A.S., Schneider, T., Cyran, L., Eckert, I.N., Kerstan, A., and Lutz, M.B. (2021). Conversion of Anergic T Cells Into Foxp3(-) IL-10(+) Regulatory T Cells by a Second Antigen Stimulus In Vivo. Front. Immunol. *12*, 704578. https://doi.org/10.3389/fimmu.2021.704578.

36. Zheng, M., Zhang, Z., Yu, L., Wang, Z., Dong, Y., Tong, A., and Yang, H. (2023). Immune-checkpoint protein VISTA in allergic, autoimmune disease and transplant rejection. Front. Immunol. *14*, 1194421. https://doi.org/10.3389/fimmu.2023.1194421.

37. Wang, L., Rubinstein, R., Lines, J.L., Wasiuk, A., Ahonen, C., Guo, Y., Lu, L.F., Gondek, D., Wang, Y., Fava, R.A., et al. (2011). VISTA, a novel mouse Ig superfamily ligand that negatively regulates T cell responses. J. Exp. Med. *208*, 577–592. https://doi.org/10.1084/jem.20100619.

38. Beissert, S., He, H.T., Hueber, A.O., Lellouch, A.C., Metze, D., Mehling, A., Luger, T. A., Schwarz, T., and Grabbe, S. (1998). Impaired cutaneous immune responses in Thy-1-deficient mice. J. Immunol. *161*, 5296–5302.

39. Gonzalez-Cabrero, J., Wise, C.J., Latchman, Y., Freeman, G.J., Sharpe, A.H., and Reiser, H. (1999). CD48-deficient mice have a pronounced defect in CD4(+) T cell activation. Proc. Natl. Acad. Sci. USA *96*, 1019–1023. https://doi.org/10.1073/pnas.96.3.1019.

40. Lambeir, A.M., Durinx, C., Scharpé, S., and De Meester, I. (2003). Dipeptidyl-peptidase IV from bench to bedside: an update on structural properties, functions, and clinical aspects of the enzyme DPP IV. Crit. Rev. Clin. Lab. Sci. *40*, 209–294. https://doi.org/10.1080/713609354.

41. Alvisi, G., Brummelman, J., Puccio, S., Mazza, E.M., Tomada, E.P., Losurdo, A., Zanon, V., Peano, C., Colombo, F.S., Scarpa, A., et al. (2020). IRF4 instructs effector Treg differentiation and immune suppression in human cancer. J. Clin. Invest. *130*, 3137–3150. https://doi.org/10.1172/JCI130426.

42. Sun, F., Yue, T.T., Yang, C.L., Wang, F.X., Luo, J.H., Rong, S.J., Zhang, M., Guo, Y., Xiong, F., and Wang, C.Y. (2021). The MAPK dual specific phosphatase (DUSP) proteins: A versatile wrestler in T cell functionality. Int. Immunopharmacol. *98*, 107906. https://doi.org/10.1016/j.intimp.2021.107906.

43. Urry, Z., Xystrakis, E., Richards, D.F., McDonald, J., Sattar, Z., Cousins, D.J., Corrigan, C.J., Hickman, E., Brown, Z., and Hawrylowicz, C.M. (2009). Ligation of TLR9 induced on human IL-10-secreting Tregs by 1alpha,25-dihydroxyvitamin D3 abrogates regulatory function. J. Clin. Invest. *119*, 387–398. https://doi.org/10.1172/JCI32354.

44. Welz, B., Bikker, R., Junemann, J., Christmann, M., Neumann, K., Weber, M., Hoffmeister, L., Preuß, K., Pich, A., Huber, R., and Brand, K. (2019). Proteome and Phosphoproteome Analysis in TNF Long Term-Exposed Primary Human Monocytes. Int. J. Mol. Sci. *20*, 1241. https://doi.org/10.3390/ijms20051241.

45. Gao, S., Zhu, H., Zuo, X., and Luo, H. (2018). Cathepsin G and Its Role in Inflammation and Autoimmune Diseases. Arch. Rheumatol. *33*, 498–504. https://doi.org/10.5606/ArchRheumatol.2018.6595.

46. Giltiay, N.V., Chappell, C.P., Sun, X., Kolhatkar, N., Teal, T.H., Wiedeman, A.E., Kim, J., Tanaka, L., Buechler, M.B., Hamerman, J.A., et al. (2013). Overexpression of TLR7 promotes cell-intrinsic expansion and autoantibody production by transitional T1 B cells. J. Exp. Med. *210*, 2773–2789. https://doi.org/10.1084/jem.20122798.

47. Li, C., Zhao, M., Liu, X., Li, Y., Xu, B., Zhou, L., Sun, X., Sun, W., Kang, N., Ji, Z., et al. (2024). Ion channel TRPV2 is critical in enhancing B cell activation and function. J. Exp. Med. *221*, e20221042. https://doi.org/10.1084/jem.20221042.

48. Henneken, M., Dörner, T., Burmester, G.R., and Berek, C. (2005). Differential expression of chemokine receptors on peripheral blood B cells from patients with rheumatoid arthritis and systemic lupus erythematosus. Arthritis Res. Ther. *7*, R1001–R1013. https://doi.org/10.1186/ar1776.

49. Sharapova, T.N., Romanova, E.A., Sashchenko, L.P., and Yashin, D.V. (2018). Tag7-Mts1 Complex Induces Lymphocytes Migration via CCR5 and CXCR3 Receptors. Acta Naturae *10*, 115–120.

50. Wixler, V., Cromme, C., Retser, E., Meyer, L.H., Smyth, N., Mühlenberg, K., Korb-Pap, A., Koers-Wunrau, C., Sotsios, Y., Bassel-Duby, R., et al. (2015). FHL2 regulates the resolution of tissue damage in chronic inflammatory arthritis. Ann. Rheum. Dis. *74*, 2216–2223. https://doi.org/10.1136/annrheumdis-2013-205061.

51. Wang, W., Huang, X., Xin, H.B., Fu, M., Xue, A., and Wu, Z.H. (2015). TRAF Family Member-associated NF-kappaB Activator (TANK) Inhibits Genotoxic Nuclear Factor kappaB Activation by Facilitating Deubiquitinase USP10-dependent Deubiquitination of TRAF6 Ligase. J. Biol. Chem. *290*, 13372–13385. https://doi.org/10.1074/jbc.M115.643767.

52. Michel, L., Grasmuck, C., Charabati, M., Lécuyer, M.A., Zandee, S., Dhaeze, T., Alvarez, J.I., Li, R., Larouche, S., Bourbonnière, L., et al. (2019). Activated leukocyte cell adhesion molecule regulates B lymphocyte migration across central nervous system barriers. Sci. Transl. Med. *11*, eaaw0475. https://doi.org/10.1126/scitranslmed.aaw0475.

53. Gatto, D., Wood, K., and Brink, R. (2011). EBI2 operates independently of but in cooperation with CXCR5 and CCR7 to direct B cell migration and organization in follicles and the germinal center. J. Immunol. *187*, 4621–4628. https://doi.org/10.4049/jimmunol.1101542.

54. Lindh, I., Snir, O., Lönnblom, E., Uysal, H., Andersson, I., Nandakumar, K.S., Vierboom, M., t Hart, B., Malmström, V., and Holmdahl, R. (2014). Type II collagen antibody response is enriched in the synovial fluid of rheumatoid joints and directed to the same major epitopes as in collagen induced arthritis in primates and mice. Arthritis Res. Ther. *16*, R143. https://doi.org/10.1186/ar4605.

55. Backlund, J., Treschow, A., Bockermann, R., Holm, B., Holm, L., Issazadeh-Navikas, S., Kihlberg, J., and Holmdahl, R. (2002). Glycosylation of type II collagen is of major importance for T cell tolerance and pathology in collagen-induced arthritis. Eur. J. Immunol. *32*, 3776–3784. https://doi.org/10.1002/1521-4141(200212)32:12<3776::AID-IMMU3776>3.0.CO;2-A.

56. Jones, E.Y., Fugger, L., Strominger, J.L., and Siebold, C. (2006). MHC class II proteins and disease: a structural perspective. Nat. Rev. Immunol. *6*, 271–282. https://doi.org/10.1038/nri1805.

57. Lindgren, C., Tyagi, M., Viljanen, J., Toms, J., Ge, C., Zhang, N., Holmdahl, R., Kihlberg, J., and Linusson, A. (2018). Dynamics Determine Signaling in a Multicomponent System Associated with Rheumatoid Arthritis. J. Med. Chem. *61*, 4774–4790. https://doi.org/10.1021/acs.jmedchem.7b01880.

58. Mehta, N., Maddineni, S., Mathews, I.I., Andres Parra Sperberg, R., Huang, P.S., and Cochran, J.R. (2019). Structure and Functional Binding Epitope of V-domain Ig Suppressor of T Cell Activation. Cell Rep. *28*, 2509–2516.e5. https://doi.org/10.1016/j.celrep.2019.07.073.

59. Lines, J.L., Pantazi, E., Mak, J., Sempere, L.F., Wang, L., O'Connell, S., Ceeraz, S., Suriawinata, A.A., Yan, S., Ernstoff, M.S., and Noelle, R. (2014). VISTA is an immune checkpoint molecule for human T cells. Cancer Res. *74*, 1924–1932. https://doi.org/10.1158/0008-5472.CAN-13-1504.

60. Liu, J., Yuan, Y., Chen, W., Putra, J., Suriawinata, A.A., Schenk, A.D., Miller, H.E., Guleria, I., Barth, R.J., Huang, Y.H., and Wang, L. (2015). Immune-checkpoint proteins VISTA and PD-1 nonredundantly regulate murine T-cell responses. Proc. Natl. Acad. Sci. USA *112*, 6682–6687. https://doi.org/10.1073/pnas.1420370112.

61. Han, X., Vesely, M.D., Yang, W., Sanmamed, M.F., Badri, T., Alawa, J., López-Giráldez, F., Gaule, P., Lee, S.W., Zhang, J.P., et al. (2019). PD-1H (VISTA)-mediated suppression of autoimmunity in systemic and cutaneous lupus erythematosus. Sci. Transl. Med. *11*, eaax1159. https://doi.org/10.1126/scitranslmed.aax1159.

62. Broddefalk, J., Forsgren, M., Sethson, I., and Kihlberg, J. (1999). Preparation of a Diglycosylated Hydroxylysine Building Block Used in Solid-Phase Synthesis of a Glycopeptide from Type II Collagen. J. Org. Chem. *64*, 8948–8953. https://doi.org/10.1021/jo990853d.

63. Miller, E.J. (1972). Structural studies on cartilage collagen employing limited cleavage and solubilization with pepsin. Biochemistry *11*, 4903–4909. https://doi.org/10.1021/bi00776a005.

64. James, J., Chen, Y., Hernandez, C.M., Forster, F., Dagnell, M., Cheng, Q., Saei, A.A., Gharibi, H., Lahore, G.F., Åstrand, A., et al. (2022). Redox regulation of PTPN22 affects the severity of T-cell-dependent autoimmune inflammation. Elife *11*, e74549. https://doi.org/10.7554/eLife.74549.

65. Topping, L.M., Romero-Castillo, L., Urbonaviciute, V., Bolinsson, H., Clanchy, F.I., Holmdahl, R., Bäckström, B.T., and Williams, R.O. (2022). Standardization of Antigen-Emulsion Preparations for the Induction of Autoimmune Disease Models. Front. Immunol. *13*, 892251. https://doi.org/10.3389/fimmu.2022.892251.

66. Tong, D., Lönnblom, E., Yau, A.C.Y., Nandakumar, K.S., Liang, B., Ge, C., Viljanen, J., Li, L., Bălan, M., Klareskog, L., et al. (2018). A Shared Epitope of Collagen Type XI and Type II Is Recognized by Pathogenic Antibodies in Mice and Humans with Arthritis. Front. Immunol. *9*, 451. https://doi.org/10.3389/fimmu.2018.00451.

67. Viljanen, J., Lönnblom, E., Ge, C., Yang, J., Cheng, L., Aldi, S., Cai, W., Kastbom, A., Sjöwall, C., Gjertsson, I., et al. (2020). Synthesis of an Array of Triple-Helical Peptides from Type II Collagen for Multiplex Analysis of Autoantibodies in Rheumatoid Arthritis. ACS Chem. Biol. *15*, 2605–2615. https://doi.org/10.1021/acschembio.0c00680.

68. Radu, C.G., Ober, B.T., Colantonio, L., Qadri, A., and Ward, E.S. (1998). Expression and characterization of recombinant soluble peptide: I-A complexes associated with murine experimental autoimmune diseases. J. Immunol. *160*, 5915–5921.

69. Kotov, D.I., and Jenkins, M.K. (2019). Peptide:MHCII Tetramer-Based Cell Enrichment for the Study of Epitope-Specific CD4(+) T Cells. Curr. Protoc. Immunol. *125*, e75. https://doi.org/10.1002/cpim.75.

70. Chambers, M.C., Maclean, B., Burke, R., Amodei, D., Ruderman, D.L., Neumann, S., Gatto, L., Fischer, B., Pratt, B., Egertson, J., et al. (2012). A cross-platform toolkit for mass spectrometry and proteomics. Nat. Biotechnol. *30*, 918–920. https://doi.org/10.1038/nbt.2377.

71. Yu, F., Teo, G.C., Kong, A.T., Fröhlich, K., Li, G.X., Demichev, V., and Nesvizhskii, A.I. (2023). Analysis of DIA proteomics data using MSFragger-DIA and FragPipe computational platform. Nat. Commun. *14*, 4154. https://doi.org/10.1038/s41467-023-39869-5.

72. Kall, L., Canterbury, J.D., Weston, J., Noble, W.S., and MacCoss, M.J. (2007). Semi-supervised learning for peptide identification from shotgun proteomics datasets. Nat. Methods *4*, 923–925. https://doi.org/10.1038/nmeth1113.

73. da Veiga Leprevost, F., Haynes, S.E., Avtonomov, D.M., Chang, H.Y., Shanmugam, A.K., Mellacheruvu, D., Kong, A.T., and Nesvizhskii, A.I. (2020). Philosopher: a versatile toolkit for shotgun proteomics data analysis. Nat. Methods *17*, 869–870. https://doi.org/10.1038/s41592-020-0912-y.

74. Demichev, V., Messner, C.B., Vernardis, S.I., Lilley, K.S., and Ralser, M. (2020). DIA-NN: neural networks and interference correction enable deep proteome coverage in high throughput. Nat. Methods *17*, 41–44. https://doi.org/10.1038/s41592-019-0638-x.

75. Lazar, C., Gatto, L., Ferro, M., Bruley, C., and Burger, T. (2016). Accounting for the Multiple Natures of Missing Values in Label-Free Quantitative Proteomics Data Sets to Compare Imputation Strategies. J. Proteome Res. *15*, 1116–1125. https://doi.org/10.1021/acs.jproteome.5b00981.

76. Perez-Riverol, Y., Csordas, A., Bai, J., Bernal-Llinares, M., Hewapathirana, S., Kundu, D.J., Inuganti, A., Griss, J., Mayer, G., Eisenacher, M., et al. (2019). The PRIDE database and related tools and resources in 2019: improving support for quantification data. Nucleic Acids Res. *47*, D442–D450. https://doi.org/10.1093/nar/gky1106.

YMTHE, Volume *33*

Supplemental Information

Tolerogenic antigen-specific vaccine induces VISTA-enriched regulatory T cells and protects against arthritis in DRB1*04:01 mice

Laura Romero-Castillo, Rajan Kumar Pandey, Bingze Xu, Christian M. Beusch, Ana Oliveira-Coelho, Kejsi Zeqiraj, Carolin Svensson, Zhongwei Xu, Huqiao Luo, Outi Sareila, Pierre Sabatier, Changrong Ge, Lei Cheng, Vilma Urbonaviciute, Alexander Krämer, Cecilia Lindgren, Sabrina Haag, Johan Viljanen, Roman A. Zubarev, Jan Kihlberg, Anna Linusson, Harald Burkhardt, and Rikard Holmdahl

FIGURES

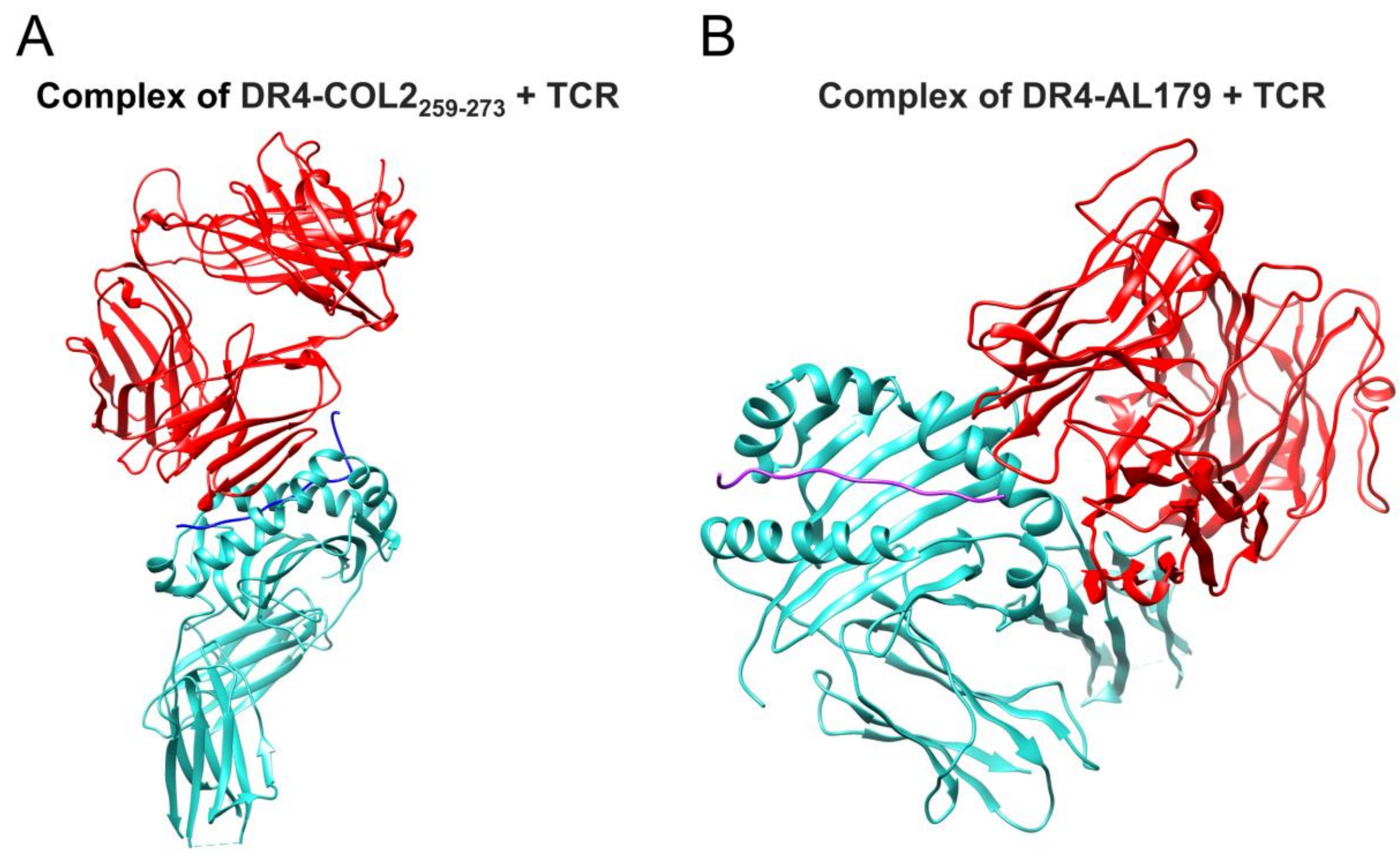

C

DR4-COL2$_{259-273}$ and TCR		DR4-AL179 and TCR	
COL2$_{259-273}$ and TCR	DR4-COL2$_{259-273}$ and TCR	AL179 and TCR	DR4-AL179 and TCR
Ala261-αSer52<br>Phe263-αThr54<br>Gly265- αThr54	βGln70-αSer61<br>αGlu55-αTyr49<br>αGln57-βGlu101	Ile2-βLeu97	βAla73-βPhe75<br>βThr77-βAsn31<br>αArg50-αThr54<br>αArg50-αLys55<br>βGlu87-βThr3<br>βTyr83-αTyr49<br>βTyr96-αSer78<br>βSer179-αGln79<br>βThr181-αGlu16<br>βThr181-αLys170<br>βLys184-αMet16

Fig. S1. Molecular docking analysis of protein-protein interactions between MHC class II-peptide complexes and T cell receptor. The crystal structures of DRB1*04:01 and T-cell receptor were retrieved from the Protein Data Bank (PDB). PyMOL mutagenesis tool was used to mutate the amino acid residues and create DRB1*04:01-COL2$_{259-273}$ and DRB1*04:01-AL179. The HDOCK docking server was used for protein-protein docking and to determine the binding interactions. (**A**) Three-dimensional representation of the docked complex between DR4-COL2$_{259-273}$ (shown in cyan and blue, respectively) and TCR (PDB ID:4UDT; shown in red). (**B**)

Three-dimensional representation of the docked complex between DR4-AL179 (shown in cyan and purple, respectively) and TCR (shown in red). (**C**) Detailed analysis of molecular interactions showing the specific amino acid residues involved in hydrogen bond formation between different MHCII-peptide complexes and TCR. α and β denote the respective chains of either MHCII (left column) or TCR (right column). Interacting residues forming the H-bonds were determined using PyMOL. The higher number of hydrogen bonds observed in the DR4-AL179/TCR complex indicates stronger binding affinity compared to the DR4-COL2$_{259\text{-}273}$/TCR complex.

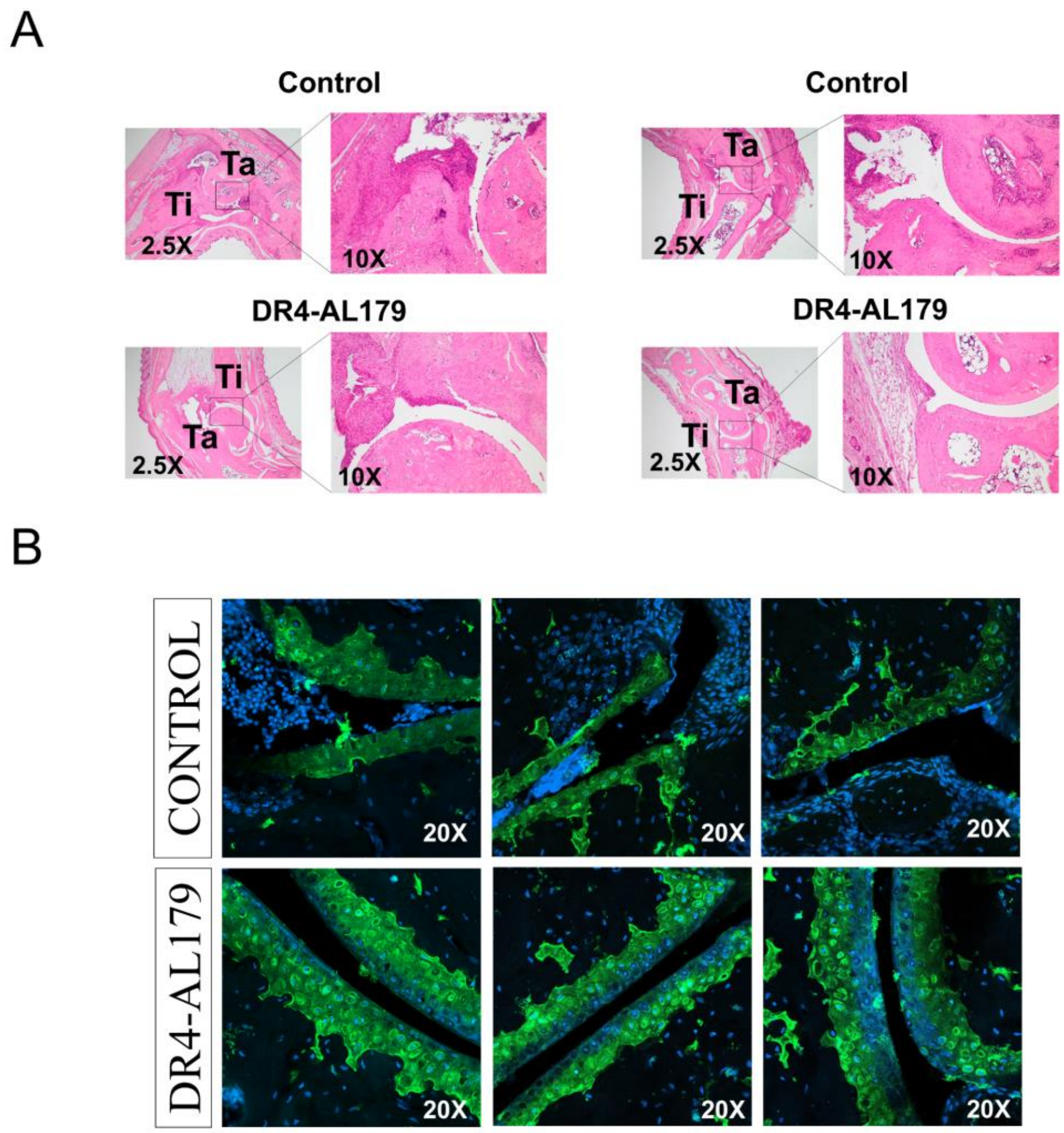

Fig. S2. Histological staining of joint tissues from DR4-CLIP (Control) and DR4-AL179 vaccinated mice in the CIA model corresponding to Figure 3E. (**A**) Hematoxylin and eosin (H&E) staining of the tibia (Ti) and talus (Ta). Control mice exhibited increased inflammatory cell infiltration and cartilage/bone erosion compared to DR4-AL179 vaccinated mice. (**B**) Immunofluorescence staining of COL2 in articular cartilage. DR4-AL179 vaccinated mice demonstrated enhanced preservation of COL2 fibrillar structure compared to the control group.

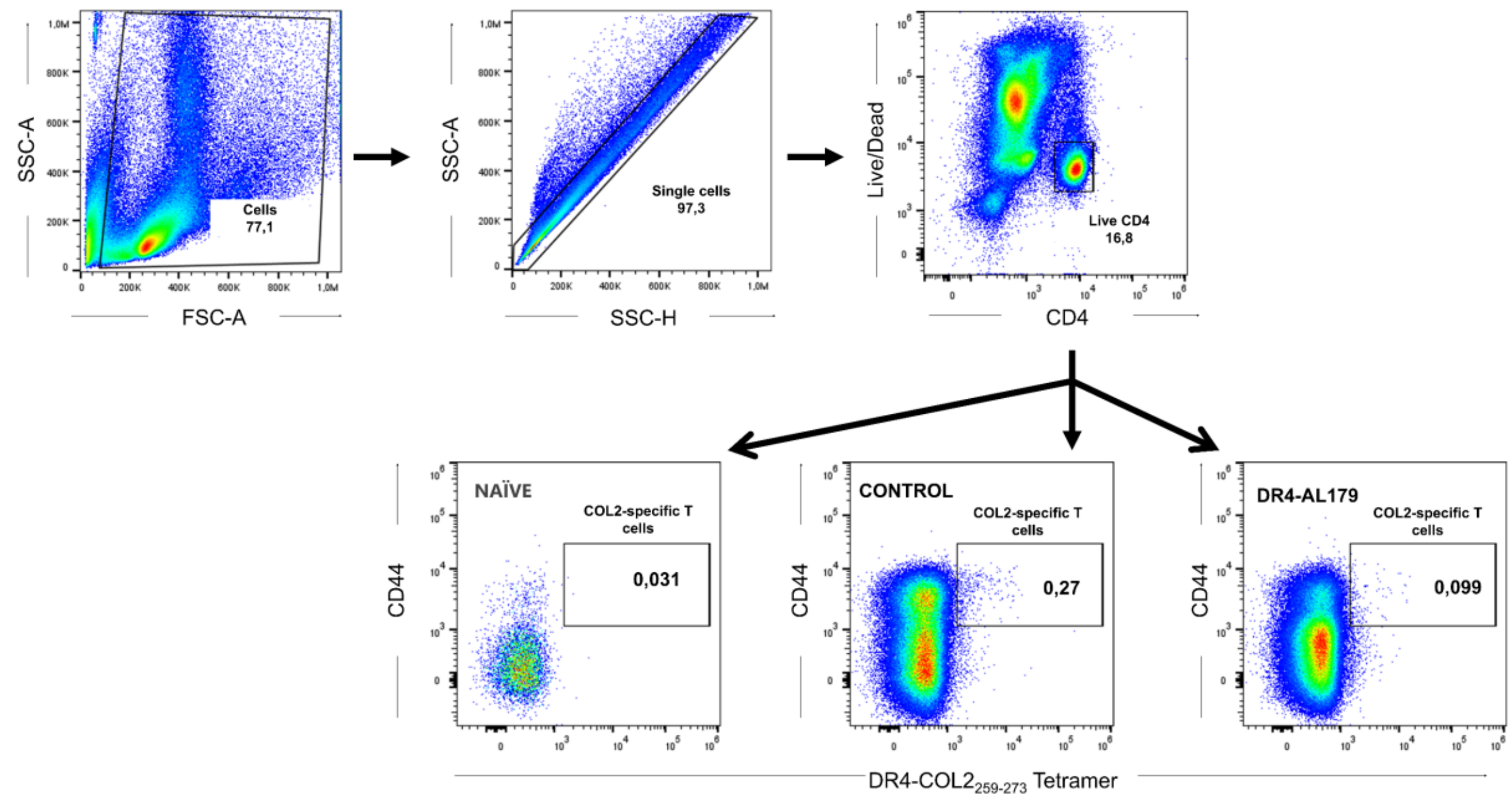

Fig. S3. Flow cytometry gating strategy for identification of COL2-specific T cells corresponding to Figure 3E. Representative gating scheme showing the sequential identification of COL2-specific T cells (CD44+ DR4-COL2$_{259\text{-}273}$ tetramer+) in spleen at day 80 of CIA model. Gates are shown for three experimental groups: mice vaccinated with DR4-CLIP (Control), mice vaccinated with DR4-AL179, and naïve (non-immunized) Parker mice. These gating strategies correspond to the data presented in Figure 3E.

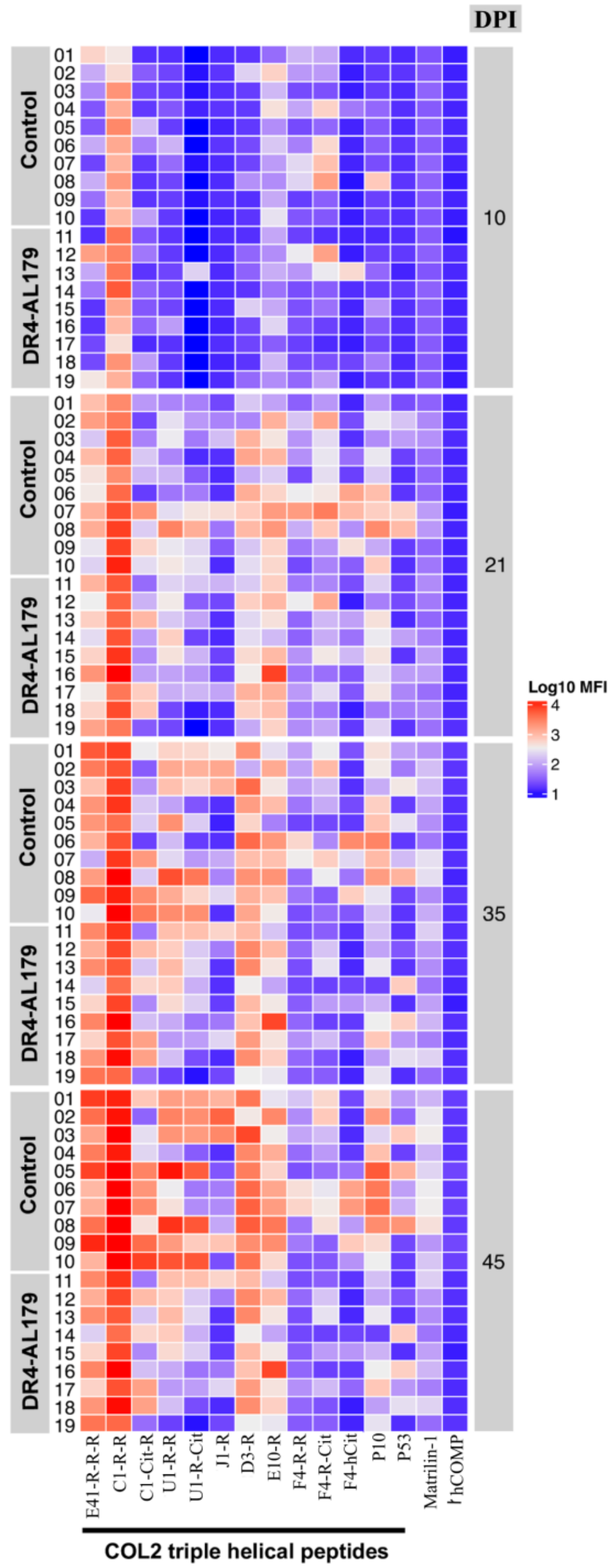

Fig. S4: Serum IgG antibody response to triple helical COL2 peptides in vaccinated mice. Parker mice were immunized with 100 µg of bCOL2 on day 0, treated with either DR4-CLIP (Control) or DR4-AL179 on days 4 and 32 postimmunization, and boosted on day 35 with 50 µg of bCOL2. Sera were collected at different days post immunization (DPI) during the CIA experiment. The antibody response to triple helical COL2 peptides or to matrilin-1 and hCOMP proteins (human cartilage oligomeric matrix protein) was detected by

bead-based multiplex flow immunoassay. Log10-transformed MFI values are presented as a heatmap. R: arginine. Cit: citrulline. hCit: homocitrulline. MFI: median fluorescence intensity.

A

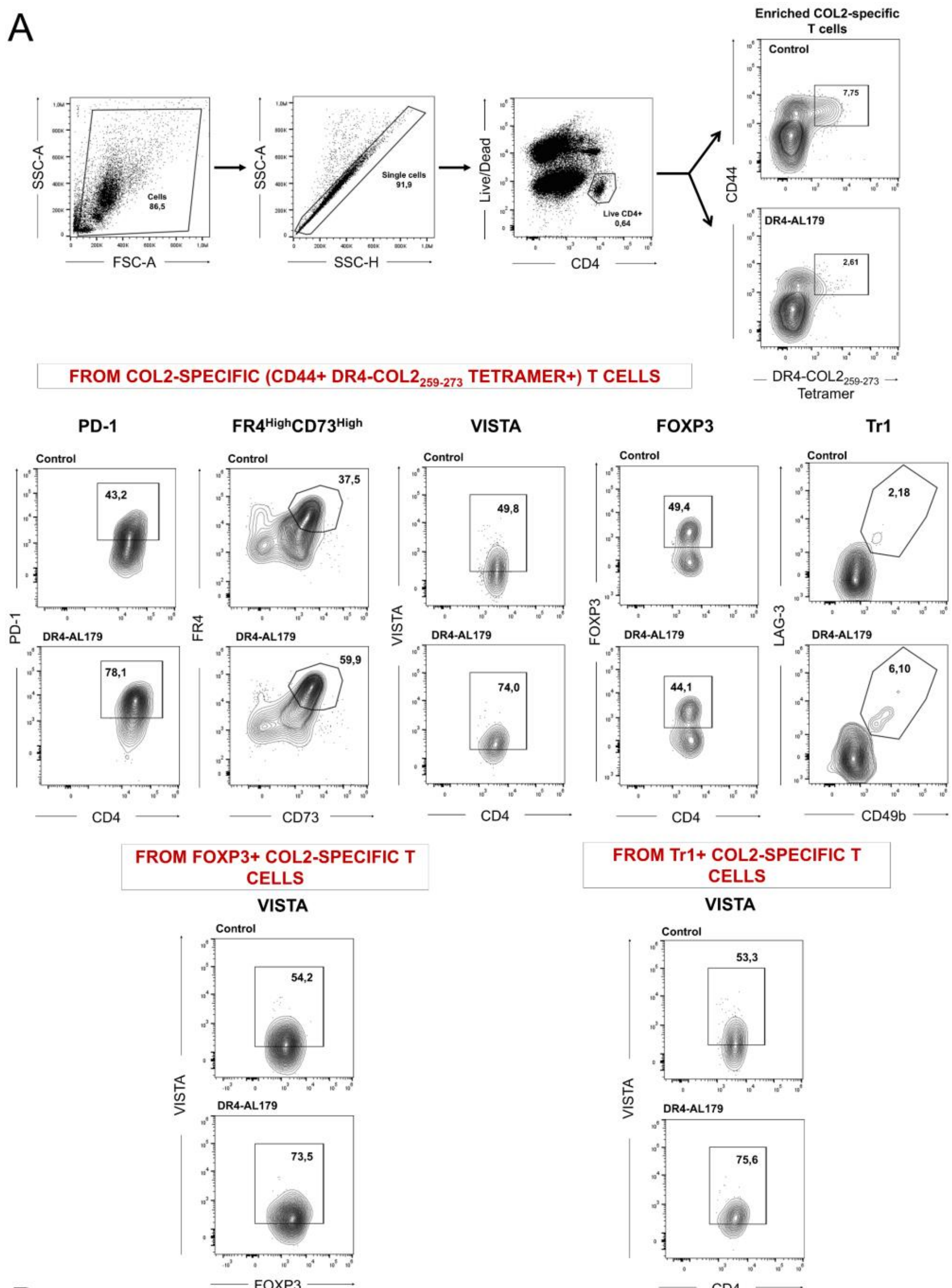

B

			Live CD4+ T cells	*COL2-specific cells*						
**Mouse**	**Treatment group**	**Live CD4 (%)**	**COL2-specific T cells (%)**	**PD-1 (%)**	**CD73highFR4high T cells (%)**	**VISTA (%)**	**FOXP3 (%)**	**Tr1 (CD49+ LAG-3+) cells (%)**	**VISTA (%) in FOXP3+ cells**	**VISTA (%) in Tr1 cells**
1	DR4-CLIP	0,64	12,9	58,9	37,5	42,7	49,7	7,52	37,2	51,5
2	DR4-CLIP	0,143	7,75	43,2	32,6	49,8	45,1	2,18	41,4	53,3
3	DR4-CLIP	0,81	6,76	42,5	26,5	54,7	58,4	0,9	48,4	59,3
4	DR4-CLIP	0,97	6,68	49	40,5	53	44,6	3,53	47,2	59,5
5	DR4-CLIP	1,24	7,27	59,8	26,6	58,7	46,7	2,78	52,1	62,6
6	DR4-AL179	0,62	4,98	78,1	50,9	60,9	54,7	28,2	53,9	64,9
7	DR4-AL179	0,67	2,97	75,1	49,3	68,9	44,1	6,1	69	70,7
8	DR4-AL179	1,22	1,43	82,1	57,4	74	38,8	8,2	69,9	75,6
9	DR4-AL179	1,03	2,61	83,5	49,4	60,8	44,3	8,1	56,4	64,2
10	DR4-AL179	0,45	4,42	80,4	54,5	75,6	23,3	11,1	70,3	77,2

Fig. S5. Flow cytometry gating strategy and quantitative analysis of immune cell markers corresponding to Figure 4. (**A**) Detailed gating strategy showing the analysis of the enriched COL2-specific T cells from

Control (DR4-CLIP) and DR4-AL179 vaccinated Parker mice. The sequential gating shows: lymphocytes from total cells, single cells, live CD4+ cells and COL2-specific T cells (defined by CD44+ DR4-COL2$_{259\text{-}273}$ tetramer). Within the COL2-specific T cell population, subsequent analyses show gating strategies for: PD-1+, FR4^{high}CD73^{high} T cells, VISTA+, FOXP3+ cells, and Tr1 cells. Additional analyses show VISTA+ on both FOXP3+ and Tr1 cells within the COL2-specific population. (**B**) Comprehensive table summarizing the percentages of each analyzed cell population per individual mouse as presented in Figure 4.

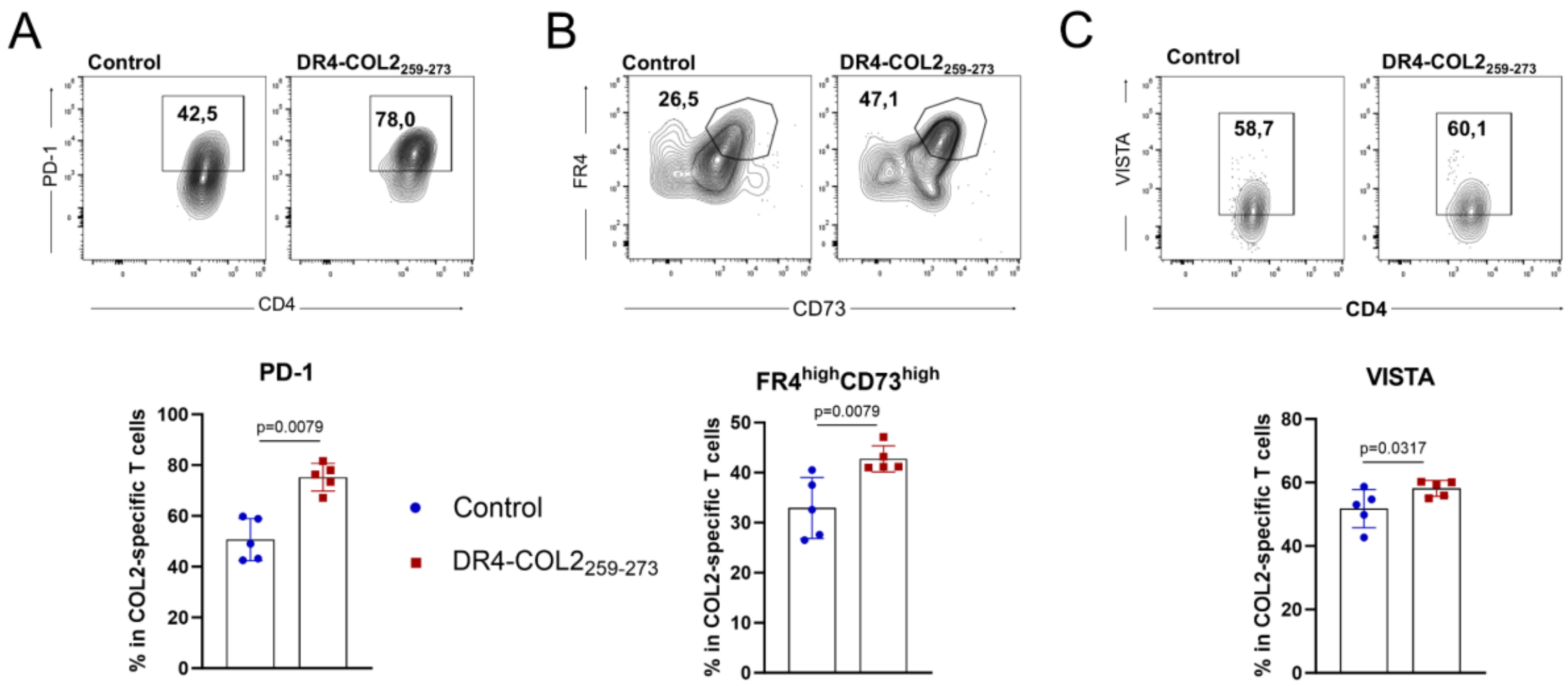

Fig. S6: Tolerogenic phenotype on COL2-specific T cells after DR4-COL2$_{259-273}$ treatment. Parker mice were immunized with COL2/CFA, at 4 DPI mice were vaccinated with either DR4-CLIP or DR4-COL2$_{259-273}$ complexes by 3 s.c injections. 15 DPI splenocytes and dLN cells were recovered, stained with the PE-labeled COL2-DR4 tetramer and enriched using anti-PE magnetic beads for immunophenotyping. (**A** to **C**) Vaccination with the DR4-COL2$_{259-273}$ complexes increases the frequency of PD-1, CD73, FR4, and VISTA among COL2-specific (DR4-COL2$_{259-273}$+/CD44+) CD4+ T cells. Representative flow cytometry plots depicting the gating strategy for PD-1, CD73, FR4 and VISTA in DR4-COL2$_{259-273}$+/CD44+/CD4+ T cells. Results are expressed as mean ± SEM. Each dot represents an individual mouse. Statistics were determined with a two-tailed Mann-Whitney test. (Control: DR4-CLIP; DPI: days post-immunization; s.c.: subcutaneously; dLN: draining lymph nodes).

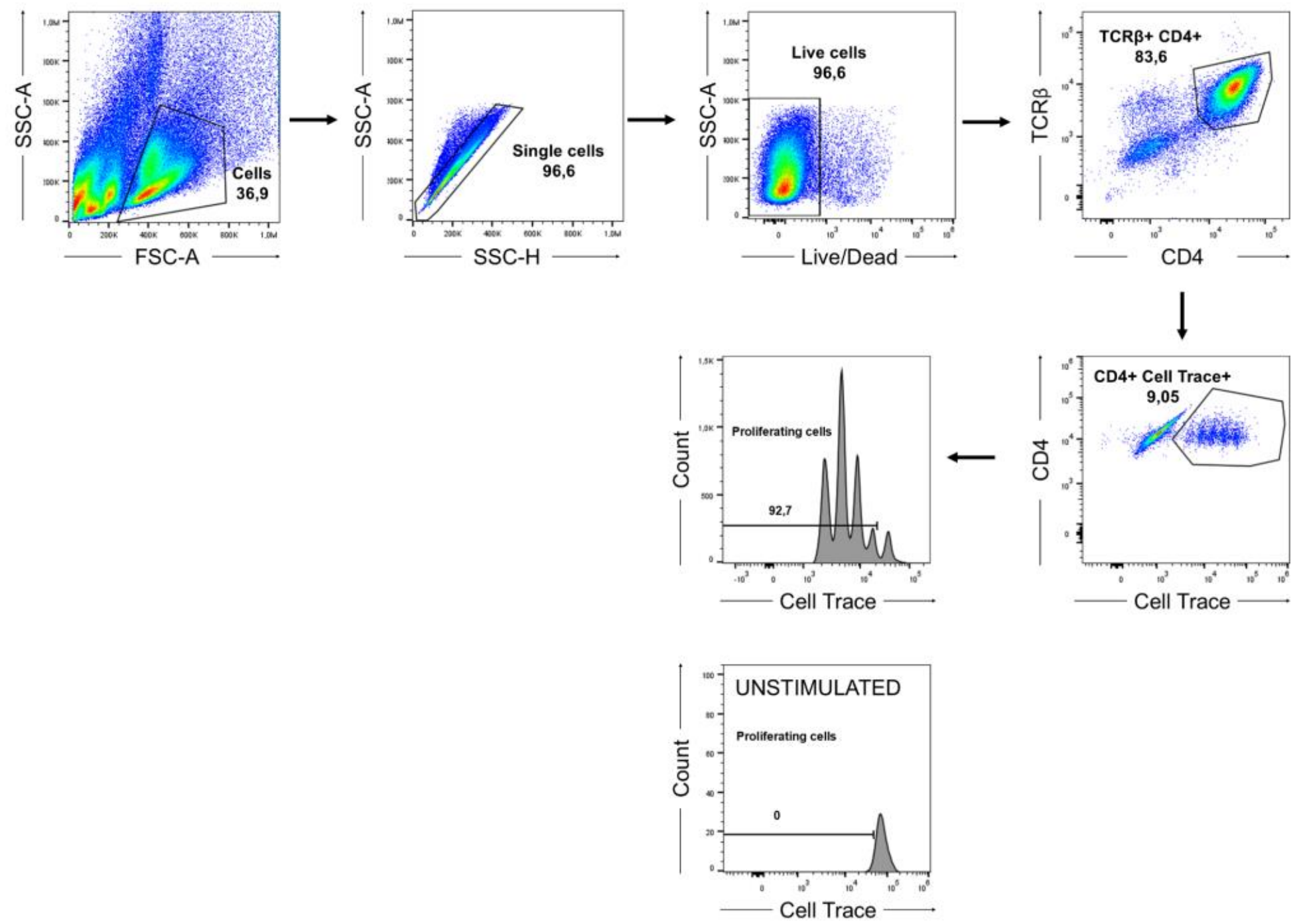

Fig. S7. Gating strategy for analysing proliferation of cell trace-labelled CD4+ T cells from naïve Parker mice in co-culture with CD4 T cells from vaccinated Parker mice corresponding to Figure 5A. Representative gating scheme showing the sequential identification of: lymphocytes, single cells, live cells, TCRβ+ CD4+ T cells, discrimination between cell trace-labelled CD4+ T cells (from naïve Parker mice) and unlabelled CD4+ T cells (from DR4-CLIP or DR4-AL179 vaccinated mice), and proliferating cells within the cell trace-positive population using unstimulated controls as reference.

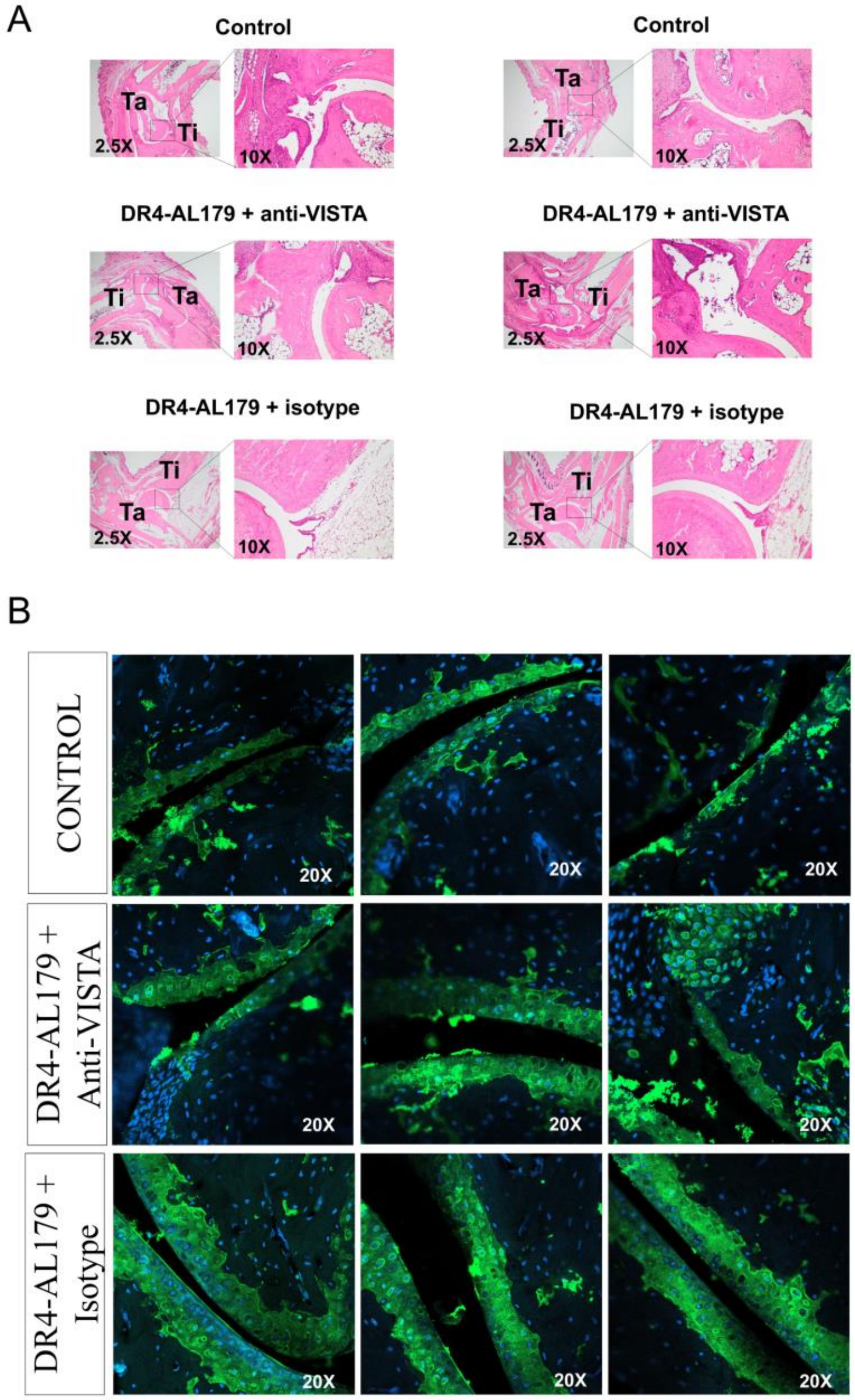

Fig. S8. Histological staining of joint tissues from DR4-CLIP (control), DR4-AL179 + Anti-VISTA and DR4-AL179 + isotype control-treated mice from the adoptive T cell transfer experiment corresponding to Figure 5D. (**A**) Hematoxylin and eosin (H&E) staining of the tibia (Ti) and talus (Ta). Control and DR4-179 + Anti-VISTA groups exhibited increased inflammatory cell infiltration and cartilage/bone erosion compared to DR4-AL179 + isotype control treated mice. (**B**) Immunofluorescence staining COL2 in articular cartilage. DR4-AL179 + isotype control treated mice demonstrated enhanced preservation of COL2 fibrillar structure compared to the rest of the groups.

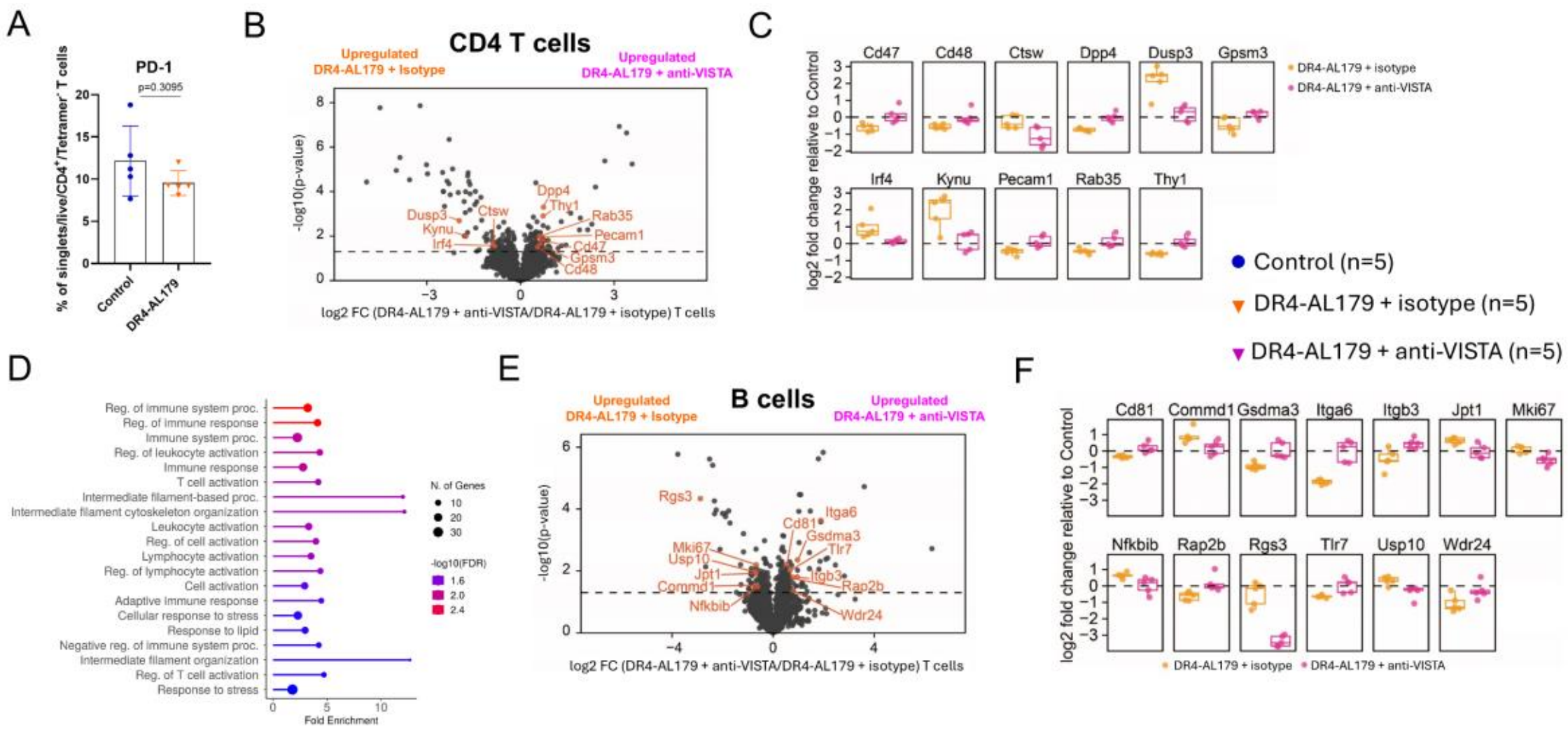

Fig. S9: T and B cell proteomic landscape. (**A**) PD-1 expression by flow cytometry after vaccination with DR4-CLIP or DR4-179 complexes. Gated in live CD4+ DR4-COL2$_{259\text{-}273}$ tetramer- cells. (**B**) Volcano plot comparing differently expressed proteins on FACS sorted CD4+ T cells between DR4-AL179 + isotype (left, orange, n = 5) and DR4-179 + anti-VISTA (right, pink, n=5) adoptively transferred mice. (**C**) Boxplots showing selected differentially expressed proteins comparing sorted CD4+ T cells from DR4-AL179 + isotype and DR4-179 + anti-VISTA (n=5 each) adoptively transferred mice. The horizontal line in the boxplots represents the median, 25th, and 75th percentiles and the whiskers represent measurements to the 5th and 95th percentiles. (**D**) Barplot illustrates the gene enrichment analysis. Each bar represents an enriched GO term. The length of the bar indicates the fold enrichment, while the associated p-value and the number of enriched genes is displayed for each term terms with an FDR < 0.05 were considered significantly enriched. (**E**) Volcano plot comparing differently expressed proteins on FACS sorted B cells between DR4-AL179 + isotype (left, orange, n = 5) and DR4-179 + anti-VISTA (right, pink, n=5) adoptively transferred mice. (**F**) Boxplots showing selected differentially expressed proteins comparing sorted B cells from DR4-AL179 + isotype and DR4-179 + anti-VISTA (n=5 each) adoptively transferred mice. The horizontal line in the boxplots represents the median, 25th, and 75th percentiles and the whiskers represent measurements to the 5th and 95th percentiles. FDR-False discovery rate. (Control: DR4-CLIP).

A

DRA_0401_BirA

MKLCILLAVVAFVGLSLGIKEEHVIIQAEFYLNPDQSGEFMFDFDGDEIFHVDMAKKETVWRLEEFGRFASFEAQGALANIAVDKANLEIMTK
RSNYTPITNVPPEVTVLTNSPVELREPNVLICFIDKFTPPVVNVTWLRNGKPVTTGVSETVFLPREDHLFRKFHYLPFLPSTEDVYDCRVEH
WGLDEPLLKHWEFDASGGGENLYFQGGGGSLTDTLQAETDQLEDEKSALQTEIANLLKEKEKLEFILAAHGGGGSGLNDIFEAQKIEWHE

DRB1_0401_hCLIPmut

MKLCILLAVVAFVGLSLGSAWSHPQFEK**PVSKARMATGALAQA**SGGGS**LVPRGS**GGGGSGDTRPRFLEQVKHECHFFNGTERVRFLDRY
FYHQEEYVRFDSDVGEYRAVTELGRPDAEYWNSQKDLLEQKRAAVDTYCRHNYGVGESFTVQRRVYPEVTVYPAKTQPLQHHNLLVCSV
NGFYPGSIEVRWFRNGQEEKTGVVSTGLIQNGDWTFQTLVMLETVPRSGEVYTCQVEHPSLTSPLTVEWRARSGGGENLYFQGGGGSRI
ARLEEKVKTLKAQNSELASTANMLREQVAQLKQKVMNHHHHHHH

B

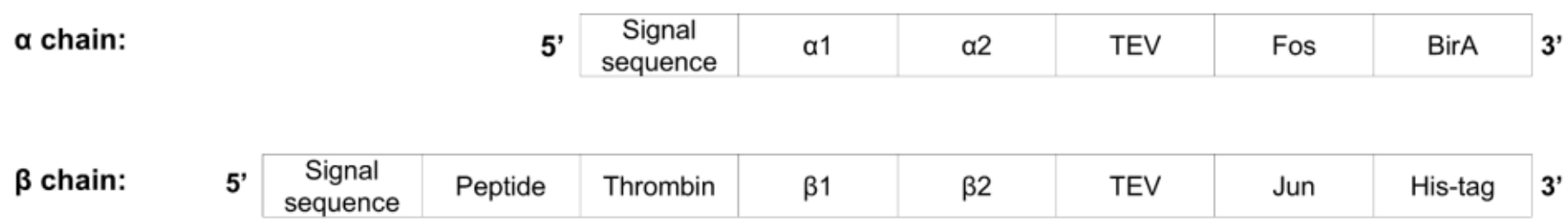

Fig. S10: Design of the DR4-peptide constructs. (**A**) Amino acid sequences of the α chain and the β chain, respectively. The text highlighted in yellow is the signal peptide; the red text indicates the covalently linked mutated human CLIP peptide; the LVPRGS text highlighted in green is a thrombin cleavage site for cleaving off the covalently linked peptide; the text highlighted in magenta indicates the dimerization motif Fos and Jun. (**B**) Features of the construct: signal peptide; C-terminal His-tag for purification; BirA tag for *in vitro* biotinylation; leucine zipper dimerization motif to form alpha and beta chains dimerization; tobacco etch virus (TEV) protease cleavage site for crystallization purposes; thrombin cleavage site (TCS) for exchange to desired peptide.

MATERIAL AND METHODS.

Molecular docking and analyzing protein-protein interactions

The crystal structures of DRB1*04:01 in complex with mutated $COL2_{259\text{-}273}$ (Sequence: AGFAGEQGPAGEP; PDB ID: 7NZF) and T-cell receptor (PDB ID: 4UDT) were retrieved from Protein Data Bank (https://www.rcsb.org/)[1,2] and the PyMOL mutagenesis tool was used to mutate the amino acid residues and create DRB1*04:01-$COL2_{259\text{-}273}$ and DRB1*04:01-AL179, ensuring structural and functional relevance. The mutated files were exported in PDB file format, maintaining conformational fidelity.

The HDOCK server[3] was used to perform protein-protein docking between DRB1*04:01-$COL2_{259\text{-}273}$ and T-cell receptor, as well as DRB1*04:01-AL179 and T cell receptor. The HDOCK server uses a computational algorithm employing a robust scoring function to predict complex formation with high accuracy. We conducted an in-depth analysis of docking scores, utilizing visualization criteria to select the most suitable model thus optimizing the predictive reliability of the docking outcomes. The PyMOL precise measurement tool was used to quantify the spatial distance between key residues involved in protein-protein interactions, ensuring meticulous characterization of binding interfaces.

Immunofluorescence staining of COL2 in joint tissue.

Paraffin-embedded joint sections were baked at 60 °C for 30 min, dewaxed using xylene, and rehydrated using gradient ethanol and finally distilled water. Masked antigens were then retrieved using 1 g pepsin (P7000, Sigma-Aldrich) dissolved in 250 ml 0.01 N HCl at 37 °C for 10 min. After antigen retrieval, slides were incubated with the primary antibody M2139

(IgG2b, Vacara AB, 10 μg/mL) at 4 °C overnight. Finally, AF488-conjugated goat anti-mouse IgG2b antibody (A-21141, ThermoFisher Scientific, 1:100, 37 °C, 1 h) was used to stain the bound IgG2b. Nuclear staining was performed using Hoechst 33342 (Invitrogen) for 10 min at room temperature. Images were acquired using an LSM800 confocal microscope (Zeiss).

Gene Enrichment Analysis

To identify biological pathways and processes associated with the differentially expressed genes, we performed gene enrichment analysis using the ShinyGO[4]. The 68 upregulated and 38 downregulated genes from the differential proteomics analysis were first converted to Ensembl gene IDs using the GRCm38 mouse genome build.

Gene Ontology (GO) Biological Process terms were used as the gene set database for the enrichment analysis. To assess whether the *Vsir* gene partners with other differentially expressed genes in certain pathways, we included *Vsir* in the gene list for the enrichment analysis.

Enrichment p-values were calculated using the hypergeometric test. To correct for multiple hypothesis testing, the Benjamini-Hochberg method was used to calculate false discovery rates (FDR). The fold enrichment for each GO term was defined as the ratio of the percentage of genes associated with that term in the query gene list compared to the percentage among all background genes.

Results of molecular docking

The protein model was obtained from PDB and mutated to create both DR4-COL2$_{259\text{-}273}$ and DR4-AL179. A total of two amino acids (GI) were added at the start of mutated COL2$_{259\text{-}273}$. Additionally, Ala61, Ala64, and Ala70 were replaced with lysine (K) to make DR4-COL2$_{259\text{-}273}$ (GIAGFKGEQGPKGEP). Later this protein model was modified at G265 to create DR4-AL179.

The docking of DR4-COL2$_{259\text{-}273}$ and TCR came out with docked models showing various H-bonds and one salt bridge. The H-bond between COL2$_{259\text{-}273}$ and TCR was Ala261-αSer52, Phe263-αThr54, and Gly265- αThr54. Moreover, the H-bond between MHCII- COL2$_{259\text{-}273}$ and TCR was βGln70-αSer61, αGlu55-αTyr49, αGln57-βGlu101. While the salt bridge was formed between αGln57-βTyr103 (Fig. S1a).

The H-bonds and the salt bridge between DR4-AL179 and TCR were also determined. The H-bond between AL179 and TCR was Ile2-βLeu97. The bonding between MHCII-AL179 and TCR were βAla73-βPhe75, βThr77-βAsn31, αArg50-αThr54, αArg50-αLys55, βGlu87-βThr3, βTyr83-αTyr49, βTyr96-αSer78, βSer179-αGln79, βThr181-αGlu16, βThr181-αLys170, βLys184-αMet167. While the salt bridge formed is β176Glu-αArg165 (Fig. S1b).

The higher number of hydrogen bonds formation between DR4-AL179 with TCR (Fig. S1c) reflects the higher binding affinity and stabilization of this complex formation compared to DR4-COL2$_{259\text{-}273}$.

REFERENCES

1. Rodstrom, K.E., Regenthal, P., and Lindkvist-Petersson, K. (2015). Structure of Staphylococcal Enterotoxin E in Complex with TCR Defines the Role of TCR Loop

Positioning in Superantigen Recognition. PLoS One *10*, e0131988. 10.1371/journal.pone.0131988.

2. Ge, C., Weisse, S., Xu, B., Dobritzsch, D., Viljanen, J., Kihlberg, J., Do, N.N., Schneider, N., Lanig, H., Holmdahl, R., and Burkhardt, H. (2022). Key interactions in the trimolecular complex consisting of the rheumatoid arthritis-associated DRB1*04:01 molecule, the major glycosylated collagen II peptide and the T-cell receptor. Ann Rheum Dis *81*, 480-489. 10.1136/annrheumdis-2021-220500.

3. Yan, Y., Tao, H., He, J., and Huang, S.Y. (2020). The HDOCK server for integrated protein-protein docking. Nat Protoc *15*, 1829-1852. 10.1038/s41596-020-0312-x.

4. Ge, S.X., Jung, D., and Yao, R. (2020). ShinyGO: a graphical gene-set enrichment tool for animals and plants. Bioinformatics *36*, 2628-2629. 10.1093/bioinformatics/btz931.
